# Metabolic programs drive function of therapeutic NK cells in hypoxic tumor environments

**DOI:** 10.1126/sciadv.adn1849

**Published:** 2024-10-30

**Authors:** Philippa R. Kennedy, Upasana Sunil Arvindam, Shee Kwan Phung, Brianna Ettestad, Xueyang Feng, Yunmin Li, Quinlan M. Kile, Peter Hinderlie, Melissa Khaw, Rih-Sheng Huang, Marissa Kaufman, Patrycja Puchalska, Amanda Russell, Jonah Butler, Lucas Abbott, Paul McClure, Xianghua Luo, Quynhanh T. Lu, Bruce R. Blazar, Peter A. Crawford, James Lim, Jeffrey S. Miller, Martin Felices

**Affiliations:** ^1^ Division of Hematology, Oncology, and Transplantation, Department of Medicine, University of Minnesota, Minneapolis, MN, USA.; ^2^ Xcell Biosciences, San Francisco, CA, USA.; ^3^ Division of Molecular Medicine, Department of Medicine, University of Minnesota, Minneapolis, MN, USA.; ^4^ Division of Biostatistics and Health Data Science, School of Public Health, University of Minnesota, Minneapolis, MN, USA.; ^5^ Division of Pediatric Blood and Marrow Transplantation and Cellular Therapy, Department of Medicine, University of Minnesota, Minneapolis, MN, USA.

## Abstract

Limited oxygen (hypoxia) in solid tumors poses a challenge to successful immunotherapy with natural killer (NK) cells. NK cells have impaired cytotoxicity when cultured in hypoxia (1% oxygen) but not physiologic (>5%) or atmospheric oxygen (20%). We found that changes to cytotoxicity were regulated at the transcriptional level and accompanied by metabolic dysregulation. Dosing with interleukin-15 (IL-15) enhanced NK cell cytotoxicity in hypoxia, but preactivation with feeder cells bearing IL-21 and 4-1BBL was even better. Preactivation resulted in less perturbed metabolism in hypoxia; greater resistance to oxidative stress; and no hypoxia-induced loss of transcription factors (T-bet and Eomes), activating receptors, adhesion molecules (CD2), and cytotoxic proteins (TRAIL and FasL). There remained a deficit in CD122/IL-2Rβ when exposed to hypoxia, which affected IL-15 signaling. However, tri-specific killer engager molecules that deliver IL-15 in the context of anti-CD16/FcγRIII were able to bypass this deficit, enhancing cytotoxicity of both fresh and preactivated NK cells in hypoxia.

## Teaser

Activation strategies alter the metabolic profile of human NK cells to overcome defects driven by hypoxic tumor microenvironments.

## INTRODUCTION

Natural killer (NK) cells are innate cytotoxic cells that can eliminate cancer cells without prior sensitization. They have great potential for cancer immunotherapy because they recognize tumor cells through an array of germline encoded receptors, including Fc receptor CD16, which drives antibody-dependent cellular cytotoxicity (ADCC); and they activate broad immune responses through secretion of cytokines (*1*, *2*). Notably, NK cell–based therapies have limited side effects (*3*–*5*). There are two main areas of NK cell–based immunotherapy: treatments that enhance endogenous NK cell function, such as the addition of cytokines, monoclonal antibodies, checkpoint inhibitors and NK cell engagers (*6*–*8*); and adoptive transfer of primary NK cells or expanded NK cells to make hundreds of doses as an “off the shelf” therapeutic product (*9*–*11*). These immunotherapies have shown promise in hematological malignancies, but their efficacy in solid tumors is limited (*12*). The solid tumor microenvironment (TME) has a wide variety of immunosuppressive mechanisms (*13*), but a key challenge is the lack of oxygen, or “hypoxia,” driven by several tumor-related factors (*14*). The severity of oxygen deprivation varies within and across tumor types, with glioblastoma, head and neck cancer, and pancreatic cancer often displaying severely hypoxic regions (*15*, *16*). While hypoxia may directly affect tumor cells, influencing stemness, metastasis, and progression across a range of cancer types (*17*–*20*), here, we focus on the direct effects of hypoxia on the NK cells themselves.

Hypoxia within the TME can be acute or chronic (*21*). Clinically, NK cells can be found circulating in patients after adoptive transfer for up to 3 weeks (*4*, *11*, *22*). Cytokine administration and repeat dosing are designed to increase circulating NK cell numbers. Previous studies on the interaction of hypoxia and interleukin-2 (IL-2)/IL-15 on NK cell function relied on high doses of cytokine that can alter metabolic requirements (*23*–*25*). Nevertheless, NK cells in hypoxia have consistently demonstrated reduced cytotoxicity when assays are performed during hypoxia (*23*, *26*), after overnight exposure (*23*, *25*), or longer intervals (*27*, *28*). This has been linked to reduced activating receptor expression and subtle defects in cytotoxic proteins (*23*, *27*). More recently, a failure to kill in hypoxia was linked to fragmented mitochondria (*28*), suggesting that metabolism plays a key role in maintaining NK cell functionality. In mice, genetic modification of hypoxia-inducible factor 1-alpha (*HIF1A*), to improve NK cell activity in hypoxia, has shown a variety of effects, positive and negative, depending on the model system used (*29*–*31*).

For T cells, hypoxia is a strong driver of exhaustion (*32*–*36*). Mitochondrial fragmentation, loss of mitochondrial biogenesis, an accompanying deficit in adenosine triphosphate (ATP) production, and the increased susceptibility to reactive oxygen species (ROS) all contribute to this impaired immunologic state. For these effector cells, activation within the hypoxic environment appears to further compound exhaustion (*34*).

Eukaryotic cells generate ATP from adenosine diphosphate (ADP) through glycolysis and oxidative phosphorylation. In oxidative phosphorylation, oxygen is required to accept electrons from the electron transport chain and generate a proton gradient. In doing so, ROS are generated. An imbalance in ROS can contribute to an impaired functional state on immune cells. Where the lack of availability of oxygen limits oxidative phosphorylation, cells must rely on other metabolic processes to generate ATP. How much NK cells rely on these two metabolic processes for cytotoxicity and cytokine production likely depends on their mode of activation.

Stimulating NK cells with IL-2 or IL-15 (*37*, *38*), IL-12/IL-18/IL-15 (*39*), or IL-21/41-BBL bearing feeder cells for adoptive transfer (*40*) increases glycolysis and oxidative phosphorylation. Blocking oxidative phosphorylation in primary NK cells prevents the enhanced release of cytotoxic granules that normally follows cytokine stimulation (*37*). Blocking oxidative phosphorylation in IL-21/4-1BBL–activated NK cells has been reported to have no impact on cytotoxicity (*41*) or to strongly inhibit cytotoxicity if focusing on a subset of the NK cells that lack inhibitory receptors that bind self human leukocyte antigens (*40*). Metabolic requirements can differ for interferon-γ (IFN-γ) production depending on whether the stimulus for release was a cytokine (IL-12/IL-18) or an activating receptor (NK1.1) (*42*). In this study, we comprehensively examined the cytotoxic, proliferative, proteomic, transcriptomic, and metabolic state of NK cells over 7 days in hypoxia to determine which elements of these cells contribute to functional deficits to determine optimal therapeutic strategies to enhance NK cell immunotherapy efficacy to treat cancer.

## RESULTS

### Hypoxia diminishes NK cell proliferation

Healthy donor NK cells were incubated in different oxygen (O_2_) and pressure conditions for up to 7 days to model levels found in arterial blood (12% O_2_, 2 psi) or bone marrow (5% O_2_, 0.6 psi) (*43*) or within the TME (1% O_2_) at low or high pressure (0.3 or 2 psi) (*44*–*46*). Cells were then removed from the incubators, and assays were performed to assess NK cell proliferation, cytotoxicity, gene expression, and metabolism. NK cells incubated in a conventional 5% CO_2_ incubator (20% O_2_) served as a control (“normoxia”). Our initial focus was to identify conditions that would allow us to investigate NK cell function over time under conditions expected in the TME. We found that once the culture media was replaced on day 4 to avoid acidification in 1% O_2_, there were no significant differences in NK cell viability across conditions as measured by annexin V and live/dead staining by flow cytometry (fig. S1A). Since oxygen and pressure both varied in this model, we asked whether pressure had an independent effect on NK cell proliferation and cytotoxicity in hypoxia at low or high pressure. There was essentially no impact of pressure on NK cell viability and proliferation or on cytolysis of tumor cells [discussed in Materials and Methods and in fig. S1 (B to D)]. Given these negative results, subsequent figures are presented with the focus on oxygen as the driving biological variable.

**Fig. 1.**
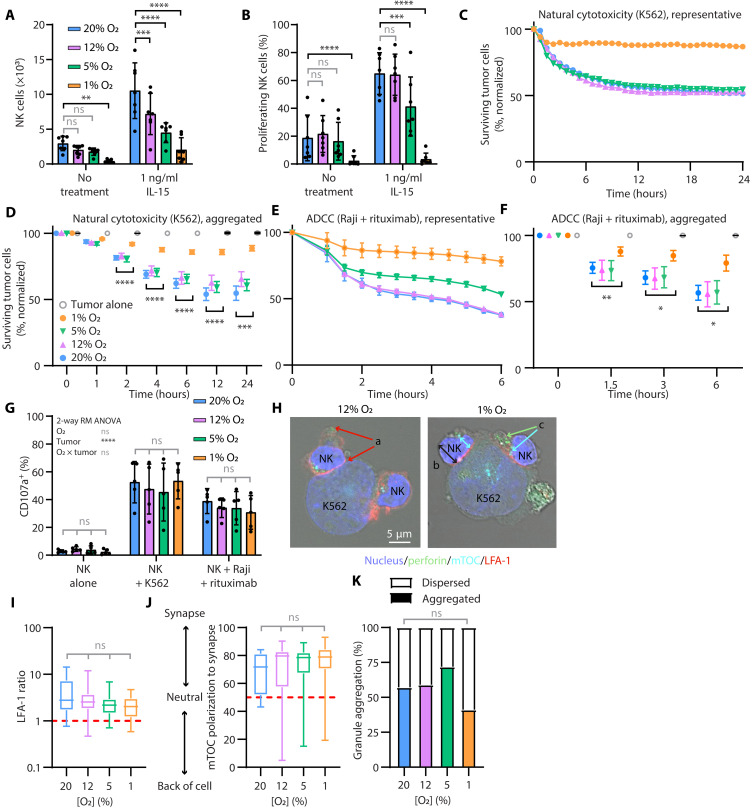
Hypoxia diminishes NK cell proliferation and cytotoxicity but not granule release. PBMCs (**A** and **B**) or pbNK cells (**C** to **K**) cells were cultured under 20% O_2_, 12% O_2_ + 2 psi, 5% O_2_ + 0.6 psi, or 1% O_2_ + 2 psi and examined on day 7. (A) Live, CD56^+^ CD3^−^ NK cells and (B) proliferating NK cells (%) were analyzed by flow cytometry (*n* = 7) with two-way repeated measures (RM) Analysis of Variance (ANOVA) and post hoc Dunnett’s comparisons (horizontal bars). [(C) to (F)] Live cell imaging assessed [(C) and (D)] natural cytotoxicity (*n* = 10) and [(E) and (F)] ADCC (*n* = 7). Nuclight Red^+^ tumor cell survival was normalized to growth of tumor cells without NK cells. [(C) and (E)] Representative single donors are shown alongside [(D) and (F)] quantification of multiple donors (two-way RM ANOVA, post hoc Dunnett’s comparisons comparing each condition within a time point to 20% O_2_, indicated by horizontal bars if *P* ≤ 0.05). [(G) to (K)] The cytotoxic process was examined through (G) quantification of granule release (*n* = 5, analyzed by two-way RM ANOVA) by flow cytometry after 5 hours and [(H) to (K)] polarization of the cytotoxic machinery to the synapse. [(H) to (K)] NK cells incubated with K562 for 5 min at 37°C, 20% O_2_, were fixed and stained for LFA-1, the mTOC, perforin, and nuclei. An example of confocal assessment of synapse formation is shown in (H). Scale bar, 5 μm. Cells were scored for “a” enrichment of LFA-1 at the synapse compared to the back of the cell, “b” polarization of the mTOC toward the synapse, and “c” aggregation of perforin granules around the mTOC; quantified in (I) to (K), *n* = 2, minimum 14 cells per condition; [(I) and (J)] compared by Kruskal-Wallis test (horizontal bars); (K) analyzed by Fisher Exact test comparing each condition with 20% O_2_, but *P* > 0.05 for all. Vertical bars show mean and SD [(A), (B), and (G)] or SEM [(C) to (F)] or median, range, and interquartile range (IQR) [(I) to (K)]. ns, not significant. **P ≤ 0.01, ***P ≤ 0.001, and ****P ≤ 0.0001.

To evaluate how oxygen levels affect proliferation, NK cells were CellTrace labeled, and dilution of the dye was evaluated at day 7. Oxygen had a titratable effect on proliferation and NK cell numbers, with the least proliferation and fewest NK cells obtained from the 1% O_2_ condition (Fig. 1, A and B). It should be noted that the severe hypoxia condition with higher pressure was selected to represent the TME going forward (*44*–*46*), since it best mimics the challenges to overcome in the hostile TME. To account for cell interactions, proliferation assays were performed in the presence or absence of other peripheral blood mononuclear cells (PBMCs), which might influence the response of NK cells to hypoxia over 7 days (fig. S2, A and B). There were greater numbers of NK cells achieving six or seven divisions when cultured with PBMCs compared to when enriched NK cells were isolated and placed alone in normoxia (fig. S2A). This is consistent with the capacity of monocytes and dendritic cells to enhance proliferation in part by the trans-presentation of IL-15 through the IL-15 receptor alpha expressed on these cell types (*47*). In contrast to normoxia, coculture with PBMCs did not enhance proliferation of NK cells in severe hypoxia (1% O_2_), suggesting that other immune cells in blood are not enough to rescue this dysfunction.

To determine whether T cells themselves might be influencing the NK cell proliferative response to severe hypoxia, T cells were enriched and added back into culture with autologous donor NK cells and then subjected to normoxia or severe hypoxia for 7 days (fig. S2, C and D). In normoxia and hypoxia, NK cells divided less when T cells were present, possibly competing for IL-15. This suggests that T cells themselves are not responsible for the enhanced NK cell proliferation induced by PBMCs in normoxia but could be partially responsible for limiting proliferation when PBMCs are present with NK cells in severe hypoxia.

### Cytokine production is maintained in severe hypoxia

NK cells can secrete cytokines to activate a wider immune response. We therefore asked whether secretion of the inflammatory cytokine IFN-γ was maintained in hypoxia over time. We performed these studies with both enriched NK cells and NK cells cultured with PBMCs (fig. S3). K562 (leukemia cell line) were selected as targets for examining natural cytotoxicity by NK cells, and Raji (B cell malignancy cell line) combined with the therapeutic antibody rituximab were selected to investigate ADCC. For enriched NK cells alone (cultured for 3 or 7 days), oxygen had no overall impact on production of IFN-γ when challenged with K562 or Raji with rituximab (fig. S3, A and C). When cultured with PBMCs, NK cell secretion of IFN-γ in response to these tumor cells was influenced by oxygen availability. After NK cells were cultured with PBMCs for 3 days, normoxic NK cells produced more IFN-γ than hypoxic NK cells when triggered through natural cytotoxicity, but not ADCC (fig. S3B). After NK cells were cultured with PBMCs for 7 days, higher oxygen negatively affected cytokine production (fig. S3D). Cytokine production declined with cell division in all cases (fig. S3E), so in severe hypoxia the lack of cell division sustained cytokine production over time (fig. S3D). These studies show that oxygen content has a time-dependent effect on IFN-γ production influenced by interaction with other immune cells found in blood emphasizing the balance of many variables involved in cytokine production.

### Hypoxia diminishes NK cell cytotoxicity

We used a live cell imaging assay to follow cytolysis of tumor cells through a nuclear fluorescent reporter in K562 and Raji cells and an indicator of apoptosis (Caspase-3/7 Green). We determined that a ratio of 2:1 was sensitive to differences in natural cytotoxicity and ADCC imparted by oxygen content on NK cells (fig. S4). NK cells incubated at oxygen concentrations of 5% or higher controlled tumor cells to a similar extent, but after exposure to severe hypoxia, both natural cytotoxicity and ADCC were significantly impaired (Fig. 1, C to F). Thus, killing was more sensitive to severe hypoxia than cytokine production. Considering this and the apparent importance of cytotoxicity for tumor control, we focused our investigations on the origin of the cytotoxic deficit.

### Cytotoxic granule release is sustained, despite the deficit in killing

There are several steps that lead to efficient NK cell cytotoxicity, including adhesion through integrins, aggregation of cytotoxic granules around the microtubule organizing center (mTOC), polarization of the mTOC to the synapse, followed by degranulation (*48*). To determine how different oxygen levels affect NK cell degranulation, we measured the accumulation of granule protein CD107a at the NK cell surface when challenged with K562 or Raji cells with rituximab. Unexpectedly, there were no differences in the extent of degranulation after NK cells were incubated in the different oxygen conditions (Fig. 1G). In line with this, we confirmed by imaging that there were no differences in the recruitment of integrin LFA-1 to the synapse, polarization of the mTOC to the synapse, or perforin-bearing granule aggregation around the mTOC (Fig. 1, H to K). Nor were there any differences in the frequency with which NK cells formed conjugates with tumor cells (fig. S5). NK cell cytotoxicity started to decrease as early as 3 days under hypoxic conditions with lower natural cytotoxicity and ADCC (fig. S6). There was a decrease in degranulation after 3 days in 1% O_2_ compared to 20% O_2_ when triggered by ADCC (fig. S7A). Examining the impact of PBMCs on degranulation (fig. S7), there was a more substantial impairment in degranulation for both natural cytotoxicity and ADCC on day 3 of culture in hypoxia compared to normoxia, but these differences disappeared by day 7 (fig. S7, A to D), again highlighting the time dependence of this effect. Analyzing the impact of cell divisions on degranulation, considerable degranulation still occurred after more than one division (fig. S7E). Throughout all these assays, degranulation in the context of tumor cells was significantly higher than unstimulated controls, suggesting that whatever was causing the failure to kill tumors cells (Fig. 1, C to F) was not a failure to release cytotoxic granules and the mechanistic defect must be at another level.

### Cytotoxicity in hypoxia is impaired against solid tumor cell lines

**Fig. 2.**
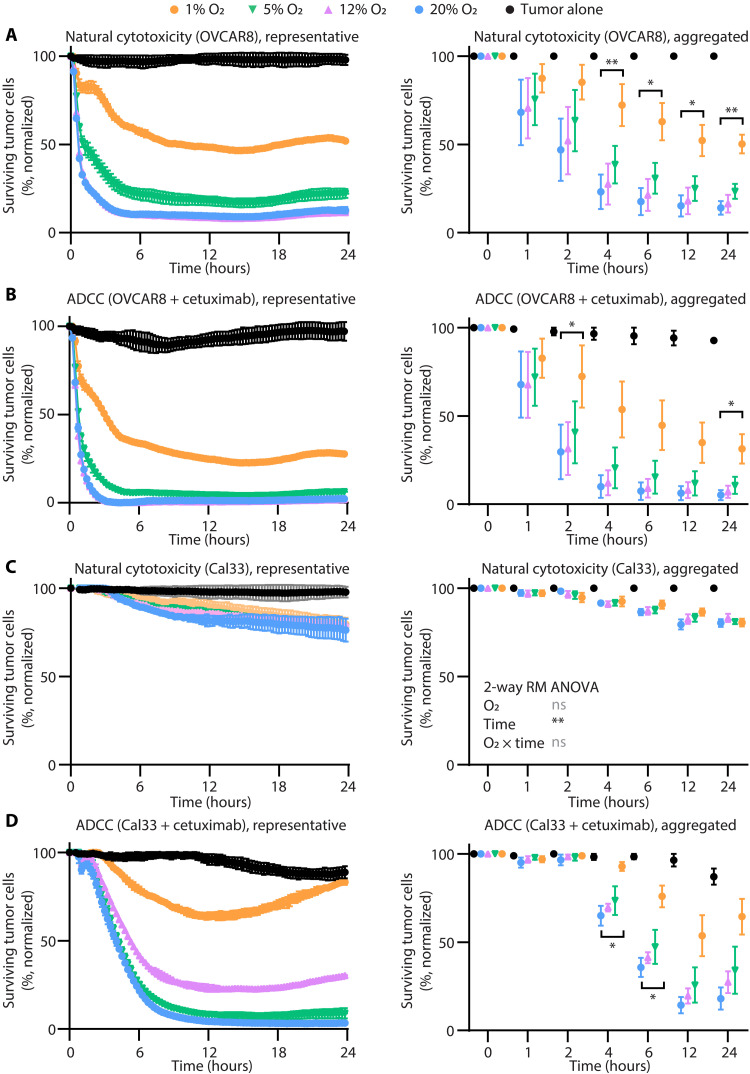
Severe hypoxia diminishes NK cell cytotoxicity of solid tumor lines. NK cells were enriched from blood and cultured (under 20% O_2_ with atmospheric pressure, 12% O_2_ + 2 psi, 5% O_2_ + 0.6 psi, or 1% O_2_ + 2 psi) for 7 days and then examined for their ability to kill solid tumor lines by natural cytotoxicity or ADCC with cetuximab. Impedance was used as a measure of solid tumor cell survival, for natural cytotoxicity-sensitive ovarian cancer line, OVCAR8 (**A** and **B**), and natural cytotoxicity-resistant head and neck cancer line, Cal33 (**C** and **D**), normalized to the growth of tumor cells alone without cetuximab. The effector:target cell ratio (4:1) was selected for greatest sensitivity to differences between groups, and the dose of cetuximab was selected for each cell line to have the minimal direct impact on tumor cell growth alone but maximal ADCC. In representative graphs, conditions were measured in duplicate, and vertical bars show SD. In aggregate graphs, data from four independent donors are shown, and vertical bars show SEM, analyzed by two-way RM ANOVA. If O_2_ failed to affect the result, then this is reported within the graph. If O_2_ affected the result (*P* ≤ 0.05), then a post hoc Dunnett’s multiple comparisons test was applied, comparing all conditions to 20% O_2_ within each time point, indicated by horizontal bars for any significant result, *P* ≤ 0.05. not significant, *P* > 0.05; *P ≤ 0.05; and **P ≤ 0.01.

Since solid tumors experience the most severely hypoxic conditions, we examined whether the deficit in NK cell cytotoxicity was broadly applicable to a range of malignancies (Fig. 2 and fig. S8). We selected cell lines of tumor types known to have TMEs characterized by severe hypoxia like head and neck cancer (Cal33), glioblastoma (LN229), and cancers that can have more variable oxygen availability like ovarian cancer (OVCAR8) and colorectal cancer (HT29). Cal33 is extremely resistant to natural cytotoxicity, making it a good model for understanding ADCC. We used cetuximab to mediate ADCC, since all these lines express epidermal growth factor receptor. In comparison to Cal33, OVCAR8 is more sensitive to natural cytotoxicity, so that the addition of cetuximab adds little to the overall control of OVCAR8 by NK cells. Normalizing to the growth of tumor cells alone, it was apparent for all tumor types and stimulations where robust cytotoxicity was observed in normoxia that cytotoxicity was significantly diminished in severe hypoxia. As with our blood cancer models of natural cytotoxicity and ADCC, there was no statistical difference in killing by NK cells cultured in 20, 12, or 5% oxygen. Overall, these data suggest that the models of natural cytotoxicity and ADCC reveal a marked deficit in NK cell biology in severe hypoxia that is applicable across a wide range of solid tumor types, adding to the definitiveness of this finding with multiple models.

### Hypoxia reduces cytotoxic proteins, receptors, and transcription factors

**Fig. 3.**
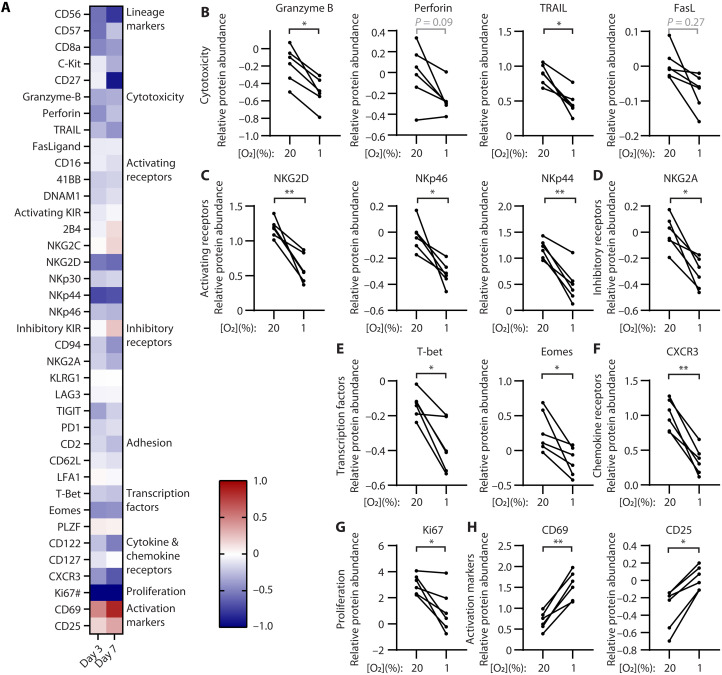
Severe hypoxia decreases NK cell receptors, cytotoxic proteins, and transcription factors but increases activation markers. NK cells were exposed to 20% or 1% oxygen for 3 to 7 days and analyzed by CyTOF. (**A** to **H**) Differential expression analysis reveals the change in protein abundance on NK cell under severe hypoxia (1% O2 + 2 psi), relative to normoxia (20% O_2_), as quantified in Astrolabe Diagnostics software. Positive fold changes indicate up-regulation in hypoxia. In (A), each square is the average value for all donors after 3 days or 7 days in culture; in (B) to (H), each line represents a donor after 7 days in culture. Differential expression analysis is adjusted for multiple testing (*n* = 6). *P ≤ 0.05, **P ≤ 0.01, and *P* > 0.05 are listed in full. In (A), # indicates that the values for Ki67 are beyond the range of the listed scale (−2.66 on day 3; −2.03 on day 7).

If the toxic payload of NK cell granules was diminished in hypoxia, that could explain the disconnect between successful granule release but impaired cytotoxicity. We examined the abundance of pore-forming protein, perforin; lytic protein, granzyme B; and death receptors, tumor necrosis factor-related apoptosis-inducing ligand (TRAIL) and FasL; by time-of-flight mass cytometry (CyTOF). We included 38 antibodies against activating and inhibitory receptors, cytokine receptors, transcription factors, and activation markers to examine important cellular processes. We applied viSNE, a t-distributed stochastic neighbor-embedding algorithm, to organize NK cell subsets into “islands” (CD56^bright^ immature cells, CD57^+^ mature cells, and Ki67^+^ proliferating cells) that can be seen in the key to fig. S9. The Ki67^+^ subset was absent in 1% O_2_, but for both mature and immature NK cells, perforin decreased when NK cells were incubated in decreasing oxygen conditions over time. Activating receptor NKG2D that recognizes stress ligands on tumor cells decreased with oxygen concentration but not necessarily with time (3 and 7 days in culture induced more NKG2D than 1 day in culture). For statistical analysis, we focused on severe hypoxia (1% O_2_) and normoxia (20% O_2_) after 3 or 7 days in culture (Fig. 3). Across our panel, most were decreased in abundance in severe hypoxia compared to normoxia (Fig. 3A, blue). There was a decrease in cytotoxic proteins (Fig. 3B): granzyme B and TRAIL, with a similar trend seen for FasL and perforin, but the latter did not reach statistical significance. This suggests that the process of granule release was not impaired, but the contents of those NK cell granules were less cytotoxic in hypoxia. There was an accompanying decrease in the abundance of activating receptors (NKG2D, NKp44, and NKp46; Fig. 3C), inhibitory receptors (NKG2A and CD94; *P* = 0.013 for CD94; Fig. 3, A and D), transcription factors (Tbet and Eomes; Fig. 3E), and chemokine receptor CXCR3 (Fig. 3F) and a decrease in proliferation marker, Ki67 (Fig. 3G). NK cells require activating receptors to recognize tumor cells; they require inhibitory receptors to tune their cytotoxic function in a process known as “licensing” or “education” (*49*–*51*), and many of the cytotoxic processes are controlled by the transcription factors T-bet and Eomes (*52*–*54*), so the loss of these factors likely impairs NK cell cytotoxicity. Opposing this were the activation markers, CD69 and CD25 (Fig. 3H), that were reproducibly overexpressed in hypoxia. This observation suggests that hypoxia is inducing a broad stress response. Together with the defect in cytotoxic proteins, activating receptors, and key transcription factors, the stress response could be contributing to the impaired cytotoxicity seen in hypoxia.

We next asked whether we could observe changes in specific NK cell subsets upon exposure to hypoxia considering stages 4A (CD56^bright^), 5 (CD56^dim^), and 6 (CD57+) as the primary NK cells found in peripheral blood (*55*, *56*). In addition, stage 5 is often subdivided into immature (NKG2A^+^ KIR^−^) and mature cells (NKG2A^−^ KIR^+^) (*57*), and adaptive NK cells (NKG2C^+^ CD57^+^) are a subset of stage 6 cells that are found in high frequency in individuals who are serum positive for cytomegalovirus. Using the CyTOF panel, we evaluated the CD56^bright^ populations (CD62L^+^, CD16^−^, CD57^−^, KIR^−^), CD56^dim^ immature populations (CD62L^−^ CD57^−^ KIR^−^ NKG2A^+^), CD56^dim^ mature populations (CD62L^−^ CD57^−^ KIR^+^ NKG2A^−^), late-stage cells (CD57^+^ NKG2C^−^), and adaptive cells (CD57^+^ NKG2C^+^). Hypoxia had no significant effects on the proportions of NK cell populations (fig. S10A). Phenotypic comparison of NK cells in hypoxia compared to normoxia revealed no differences within NK cell subsets compared to the bulk NK cell population (fig. S11A). There were decreases in Ki67 and granzyme B and concomitant increases in CD25 and CD69 in all subsets. Overall, it was clear that there were no substantial changes in individual NK cell subsets upon culture in hypoxia, and the impact was global and not dependent on maturational stage.

### Shifts in gene expression underpin the loss of function in hypoxia

**Fig. 4.**
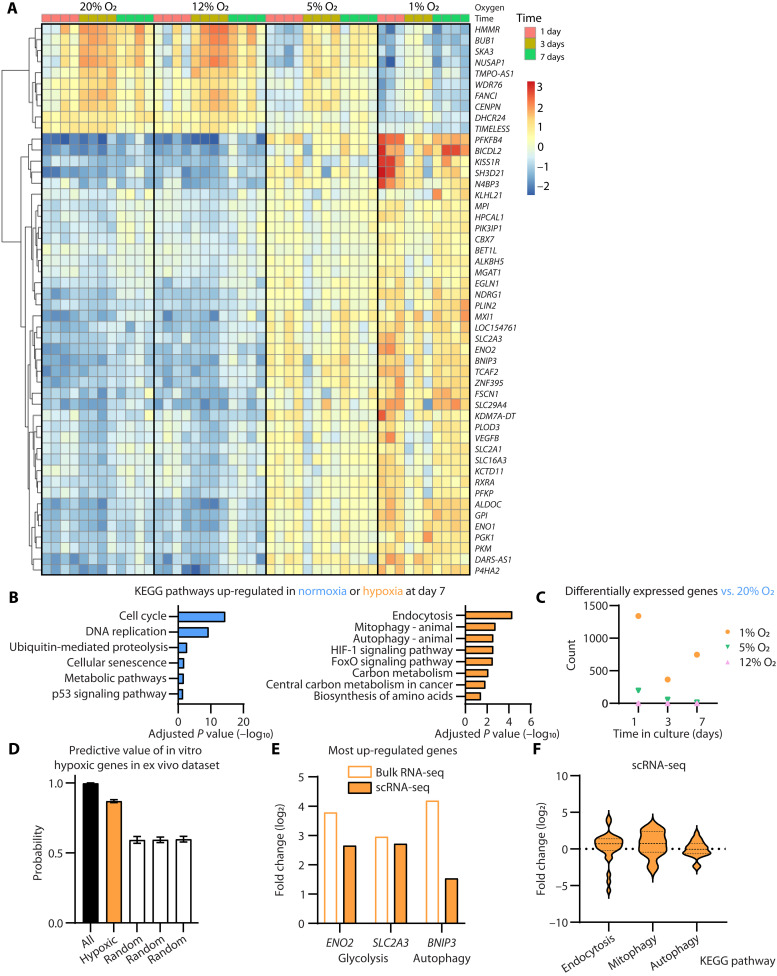
Gene expression signatures steadily change with hypoxia and are found in tumor-infiltrating NK cells from glioblastoma patients. (**A** to **C**) Enriched NK cells were cultured under 20% O_2_, 12% O_2_ + 2 psi, 5% O_2_ + 0.6 psi, or 1% O_2_ + 2 psi and harvested after 1, 3, or 7 days for RNA-seq analysis. (A) Heatmaps show the relative change in the 50 most differentially expressed genes (DEGs) across oxygen conditions and time points. Each column of small squares represents a donor. (B) Bar graphs plot the Kyoto Encyclopedia of Genes and Genomes (KEGG) pathways up-regulated with increasing oxygen concentration (blue) or up-regulated with decreasing oxygen concentration (orange) at day 7. (C) The number of DEGs in comparison to the 20% oxygen condition is highlighted for the different time and oxygen culture conditions. (**D** to **F**) Genes and pathways up-regulated in hypoxia identified by bulk RNA-seq are compared to signatures within the NK cell population of scRNA-seq datasets from patients with glioblastoma (GBM) or healthy human blood. (D) The probability of separating GBM from blood scRNA-seq data using all detected genes (>4000, positive control), a random selection of genes (six genes, negative control), or the genes shared between these ex vivo sets and the in vitro hypoxic dataset of 50 most DEGs (six genes) was calculated using a machine learning method using logistic classifier. Bars show the mean and SD. (E) The relative enrichment in the hypoxic (bulk RNA-seq) or GBM (scRNA-seq) datasets is plotted relative to normoxia (bulk RNA-seq) or blood (scRNA-seq) for three genes important in glycolysis or autophagy. (F) Gene enrichment within GBM samples relative to blood samples is plotted for genes within three KEGG pathways (endocytosis, mitophagy, and autophagy), identified as differentially regulated in our in vitro RNA-seq dataset (B).

To assess the consequences of oxygen concentration on the transcriptomic and signaling responses of NK cells, we performed an RNA sequencing (RNA-seq) analysis of NK cells incubated under different oxygen conditions for 1, 3, or 7 days. The 50 most differentially altered genes across time points and oxygen concentrations revealed different patterns of activation with time. Many of the genes in this set were strongly up-regulated at 24 hours after exposure to hypoxia and continued to show differential expression 7 days into exposure (Fig. 4A). We observed changes to pathways related to proliferation, DNA repair, and cellular metabolism. By day 7 under normoxia, there was a clear up-regulation of pathways related to DNA replication and cell cycle, which were absent in hypoxia (Fig. 4B). The mitotic checkpoint, *BUB1*, typified this expression pattern. It was up-regulated in normoxia by day 3, but not in hypoxia (Fig. 4A). In hypoxia, there was an up-regulation of pathways related to membrane and organelle recycling: endocytosis, mitophagy, autophagy; energy usage: carbon metabolism; and transcription factors: HIF-1α and forkhead box O (FOXO; Fig. 4B and fig. S12). Endocytosis is a process that internalizes exogenous material by budding membrane. In doing so, it removes membrane proteins from the cell surface, which could contribute to the loss of NK cell surface receptors observed by CyTOF (Fig. 3). Autophagy involves the recycling of cellular components within the lysosome, and mitophagy is the removal of mitochondria. These active processes suggest that NK cells are not passive in hypoxia but actively remodeling their cellular processes. In the presence of oxygen, HIF-1α is posttranslationally modified by oxygen-dependent proteases, rapidly targeting it for degradation. In the absence of oxygen, HIF-1α is stable and regulates the expression of genes that allow cells to adapt to low oxygen conditions (*19*). Proteomic regulation of HIF-1α resulted in strong up-regulation of HIF-1α–regulated genes, such as the glycolytic enzyme *PFKFB4* at 24 hours (Fig. 4A), and there is persistent up-regulation of the HIF-1α pathway after 7 days (fig. S12).

Chemokine receptors are an important factor for homing of NK cells. Our data indicate that at early time points (fig. S13A) some chemokine receptors are unregulated in hypoxia (*CCR7* and *CXCR4*), but at later time points (fig. S13B), several chemokine receptors get down regulated (*CCR1*, *CCR2*, *CCR5*, and *CXCR6*). While these changes were strongest in severe hypoxia, decreases are also seen at 5% oxygen, indicating that migration could be affected in other compartments like the bone marrow at this intermediate oxygen level.

When we looked at the total number of differentially regulated genes at day 7, we found that there was almost no difference between the standard incubator condition (20% O_2_) and the 12% O_2_ condition (2 total). There were few differences when NK cells were incubated under 5% O_2_ (<300 total), but the biggest differences were observed when NK cells were incubated under the 1% O_2_ (>2000 total; Fig. 4C). We had observed no effect of pressure on proliferation and cytotoxicity at 1% O_2_ (fig. S1), which was supported by a lack of difference in gene expression when NK cells were exposed to these conditions (<30 total). These results demonstrate two things with regard to oxygen content. First, there is a remodeling of the transcriptome when NK cells are exposed to hypoxia that is detectable at 24 hours and compounds over 7 days. Second, the largest differences are between normoxia (20% O_2_) and severe hypoxia (1% O_2_). Given the physiologic conditions throughout the body’s tissues, it is logical to conclude that these differences occur along an oxygen continuum to affect NK cell function in the microenvironment where they home.

### Hypoxic gene expression signatures are found in glioblastoma-infiltrating NK cells

Having identified pathways that are regulated in NK cells by hypoxia, we set out to determine whether these pathways would be present in NK cells extracted from human tumors. Glioblastoma is known to be hypoxic compared to peripheral blood (*15*, *58*, *59*). We mined previously published single-cell RNA-seq (scRNA-seq) datasets from peripheral blood of healthy donors (GSE130430) (*60*) and excised glioblastoma tissue (GSE147275) (*61*) for this purpose. NK cells were identified as previously described (*60*, *62*), and genes were ranked according to their expression level within each tissue. The published scRNA-seq datasets had a high level of gene dropout compared to our bulk RNA-seq datasets. When we examined the 50 most differentially regulated genes within our RNA-seq data (Fig. 4A), only eight genes could be found within the published scRNA-seq datasets, and only six could be compared for fold change between glioblastoma and blood (Fig. 4D). Nevertheless, a machine learning method using logistic classifier was able to separate blood NK cells from glioblastoma NK cells with a confidence of >0.85 (±0.01 SD) using these six genes, compared to a confidence of nearly 1 if using all detected genes (>4000; positive control) or a confidence of <0.6 (±0.02 SD) when six genes were selected at random from this list (negative control). This confirms the clinical applicability of the hypoxic gene panel we generated in vitro. The relative enrichment of three of these genes, which relate to important pathways up-regulated in hypoxia according to our RNA-seq data, is highlighted for all datasets in Fig. 4E. These include glycolytic enzyme enolase 2 (*ENO2*), glucose transporter GLUT3 (*SLC2A3*)—both part of the glycolytic pathway—and Bcl2-interacting protein 3 (*BNIP3*)—a mitochondrial protein involved in mitophagy and prolonged NK cell responses (*63*). When considering the top three pathways up-regulated in hypoxia in our RNA-seq dataset (endocytosis, mitophagy, and autophagy), genes in the top two pathways (endocytosis and mitophagy) are enriched within the glioblastoma dataset compared to blood (Fig. 4F), but autophagy genes are normally distributed across both samples. These data suggest that pathways identified in hypoxia in vitro are relevant to NK cell function in tumors in vivo.

### NK cells switch to glycolysis and have less available ATP under hypoxia

**Fig. 5.**
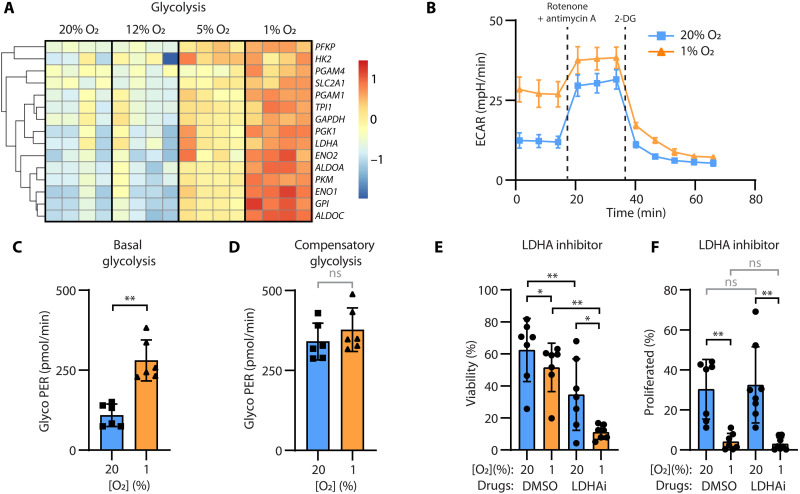
NK cells rely on glycolysis in hypoxia. (**A**) A heatmap shows the relative expression of genes in the glycolysis pathway (KEGG) after NK cells were cultured under 20% O_2_, 12% O_2_ + 2 psi, 5% O_2_ + 0.6 psi, or 1% O_2_ + 2 psi for 7 days. Each column of squares represents a donor. (**B**) Extracellular acidification rate (ECAR) of NK cells, as an indicator of aerobic glycolysis, was measured after addition of rotenone/antimycin A and 2-deoxy-d-glucose (2-DG) (representative kinetic graph, measured in triplicate). The calculated (**C**) basal and (**D**) compensatory glycolysis (rate of glycolysis following addition of Rot/AA) are plotted for six donors (paired *t* test). (**E** and **F**) NK cells were treated with 1 μM NCI-737 or dimethyl sulfoxide (DMSO) during 7-day culture. The proportion of (E) live cells and (F) proliferating cells, as indicated by dilution of CellTrace dye, was analyzed by flow cytometry (live, single, CD56^+^ CD3^−^ cells; *n* = 6; two-way RM ANOVA with Dunnett’s multiple comparisons; not significant, *P* > 0.05; *P ≤ 0.05; and **P ≤ 0.01). All bars show the mean and SD. PER, protein efflux rate; LDHAi, LDHA inhibitor.

Metabolism within a solid tumor is different from that of the surrounding tissue. In tumor cells, the result of HIF-1α activation in hypoxia is to shift energy production by increasing glycolysis and decreasing mitochondrial function (*64*). Our RNA-seq data revealed that by 7 days, NK cells in hypoxia had up-regulated genes involved in glycolysis (Fig. 5A). To confirm the increase in glycolysis, we conducted a glycolytic rate assay using a Seahorse Analyzer (Fig. 5B). NK cells incubated under hypoxia for 7 days had a higher rate of glycolysis at rest (basal; Fig. 5C) compared to normoxic NK cells at rest. The maximal capacity for glycolysis was similar for normoxic and severely hypoxic cells when mitochondria were inhibited, forcing the cells to meet all energy demands through glycolysis (compensatory; Fig. 5D). The final product of glycolysis is pyruvate. Under hypoxia, tumor cells do not use pyruvate in the mitochondria but convert it to lactate by lactate dehydrogenase (LDHA). To assess the role of LDHA in NK cells incubated under hypoxia, we added the LDHA inhibitor NCI-737 (1 μM) during the 7-day incubation. NK cell viability decreased more robustly when NK cells were treated with the LDHA inhibitor in severe hypoxia compared to normoxia (Fig. 5E), and no impact was seen on NK cell proliferation (Fig. 5F). These data are consistent with the notion that NK cells increase glycolysis under hypoxia. Unlike many cancer cell types, glycolysis supports NK cell survival but not proliferation.

**Fig. 6.**
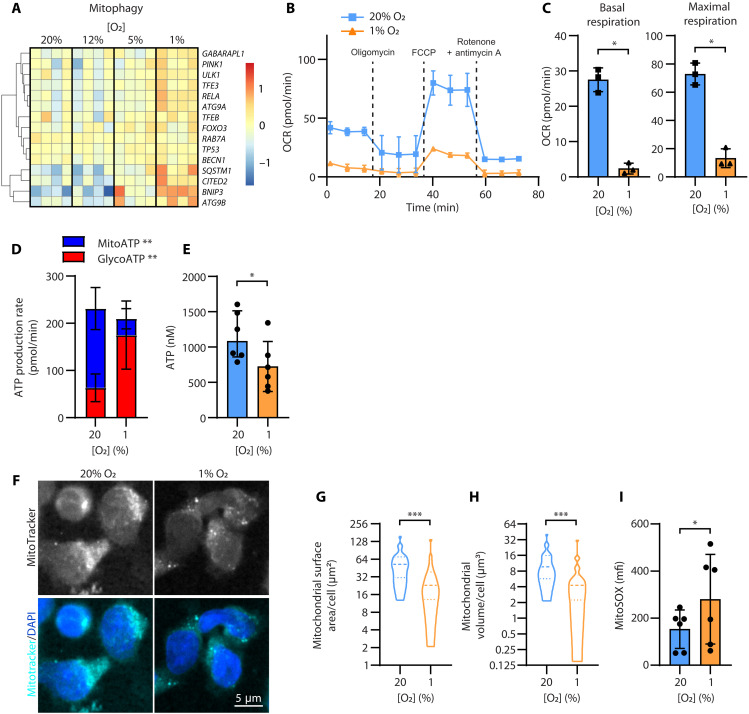
NK cells reduce mitochondrial function in hypoxia, leading to less ATP, but still considerable ROS. (**A**) A heatmap shows the relative expression of genes involved in mitophagy (KEGG) after enriched NK cells were cultured under 20% O_2_, 12% O_2_ + 2 psi, 5% O_2_ + 0.6 psi, or 1% O_2_ + 2 psi. (**B**) Oxygen consumption rate (OCR) of NK cells, as an indicator of oxidative phosphorylation, was measured after addition of oligomycin, carbonyl cyanide *p*-trifluoromethoxyphenylhydrazone (FCCP), and rotenone/antimycin A (one representative kinetic graph of three, measured in triplicate, bars shows mean and SD). (**C**) The basal and maximal respiration (following addition of FCCP) are plotted for all donors (paired *t* test). (**D**) The rate of ATP generation via glycolysis (glycoATP) or oxidative phosphorylation (mitoATP) was measured after treatment with oligomycin and rotenone/antimycin A (*n* = 7, two-way RM ANOVA comparison of 20% O_2_ versus 1% O_2_ for glycoATP or mitoATP). (**E**) The amount of ATP in NK cells was measured using a luciferase assay (*n* = 6); Wilcoxon signed rank test. (**F** to **H**) After 7 days in 20 or 1% O_2_, NK cells were stained for mitochondria (MitoTracker far red) and nuclei [4′,6-diamidino-2-phenylindole (DAPI)]. (F) Representative sum intensity z-projection images of mitochondria or mitochondria overlaid on nuclei are shown. Scale bar, 5 μm. Violin plots show quantification of mitochondrial surface area (G) and volume (H), as modeled in Imaris software. *n* = 2 donors, 20% O_2_ (48 cells), 1% O_2_ (40 cells); Mann-Whitney test. (**I**) ROS were measured by mitoSOX staining measured by flow cytometry (*n* = 6; Wilcoxon signed rank test). Bars show the mean and SD; *P ≤ 0.05, **P ≤ 0.01, and ***P ≤ 0.001.

Our RNA-seq data also revealed an increase in expression of genes relating to mitophagy in NK cells incubated under hypoxia (Fig. 6A). Oxidative phosphorylation occurs across mitochondrial membranes, and so a loss of mitochondria will affect this metabolic process. We observed a decrease in oxidative phosphorylation in severe hypoxia compared to normoxia as measured by oxygen consumption rate (Fig. 6, B and C). Thus, the relative contributions of glycolysis and oxidative phosphorylation differed for NK cells incubated in severe hypoxia or normoxia, with more ATP generated by glycolysis in hypoxia and less generated by oxidative phosphorylation relative to normoxia (Fig. 6D). Nevertheless, the overall rate of ATP production was not different for NK cells cultured in normoxia or hypoxia (Fig. 6D, sum height of bars). The available ATP within the cell was lower for NK cells cultured in hypoxia relative to normoxia (Fig. 6E). This suggests that the energy demands of hypoxic NK cells at rest are greater than for normoxic NK cells, since they generate the same amount of energy, but severely hypoxic NK cells have lower ATP reserves compared to normoxic NK cells.

Severe hypoxia was associated with a significant decrease in NK cell mitochondrial surface area and volume as measured by microscopy (Fig. 6, F to H). In addition to reducing the capacity for oxidative phosphorylation, loss of mitochondria can affect the generation of ROS. We observed a greater amount of ROS in NK cells cultured in severe hypoxia compared to normoxia at rest (Fig. 6I) presumably arising from dysfunctional mitochondria in this altered metabolic state. Overall, these results show that NK cell metabolism is altered under hypoxia, and while some aspects are similar to the metabolic shift seen in tumor cells, there are substantive differences that affect NK cell function and survival in the hypoxic TME. Considering how these variables change with different therapeutic interventions is of translational importance to maximize antitumor activity in vivo.

### Activation of NK cells with IL-15 or feeder cells enhances cytotoxicity under hypoxia

Having identified loss of granzyme B, greater reliance on glycolysis, lower ATP availability, and higher ROS at rest as features of severely hypoxic NK cells, we asked whether activation strategies being used therapeutically could overcome these limitations. A challenge for NK cell–based immunotherapy in solid tumors is cell numbers and persistence. IL-15 can enhance survival and proliferation, but we had selected a low dose of IL-15 (1 ng/ml) that induced minimal proliferation for our investigations of hypoxia. A higher pharmacologic dose of IL-15 (10 ng/ml) that induced robust proliferation in normoxia was selected to assess proliferation and cytotoxicity of peripheral blood NK (pbNK) cells in hypoxia. Another method of therapeutically enhancing NK cell function is by adoptive transfer, using feeder-expanded NK (eNK) cells to generate sufficient numbers. To replicate this process, NK cells were expanded in normoxia by culturing them with IL-2 and K562 feeder cells expressing membrane-bound IL-21 and 4-1BBL. The resulting product was frozen, and we evaluated whether these eNK cells, thawed and incubated in normoxia or hypoxia for 7 days to mimic exposure to the TME posttransfer, would show improved cytotoxicity under hypoxia.

**Fig. 7.**
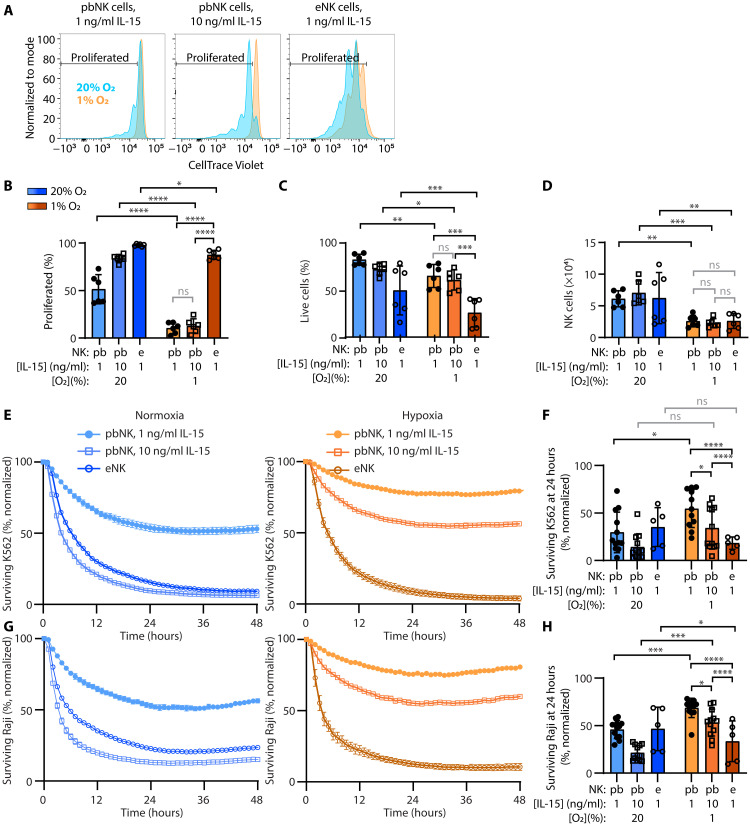
Preactivation with IL-15 or IL-2 plus feeder cells overcomes diminished cytotoxicity, but not diminished proliferation, in hypoxia. As a model of endogenous NK cell activation, pbNK cells were cultured in normoxia (20% O_2_) or hypoxia (1% O_2_) as before [IL-15 (1 ng/ml)] or with a higher dose of IL-15 (10 ng/ml) for 7 days. As a model of adoptive NK cell transfer, NK cells expanded with feeder cells (eNK) were thawed and incubated in 20 or 1% O_2_ for 7 days. (**A** to **D**) For proliferation assays, enriched NK cells were labeled with CellTrace Violet before culture. (A) Representative histograms show CellTrace dye dilution within live single NK cells (live/dead^−^, annexin V^−^, singlets, CD56^+^, CD3^−^) analyzed by flow cytometry. (B) The proportion of NK cells that proliferated [marked in (A)]. (C) The proportion of NK cells that were alive (live/dead^−^ annexin V^−^). (D) The relative number of NK cells per condition was quantified. For (A) to (D), *n* = 6, analyzed by two-way RM ANOVA with Tukey’s and Sidak’s multiple comparisons tests. (**E** to **H**) Graphs show cytotoxicity analysis of pbNK cells (*n* = 11) treated with 1 or 10 ng/ml or eNK cells (*n* = 5), killing K562 cells [(E) and (F)], or Raji cells with rituximab treatment [(G) and (H)] as in Fig. 1. Killing by representative donors over time are displayed in (E) and (G) (bars show mean and SEM of triplicate measurements). Quantification of surviving tumor cells at 24 hours after the addition of NK cells are shown in (F) and (H) (bars show the mean and SD; analyzed by mixed effects analysis with Sidak’s multiple comparisons test; not significant, *P* > 0.05; *P ≤ 0.05; **P ≤ 0.01; ***P ≤ 0.001; and ****P ≤ 0.0001.

We focused on the differences induced by 20 and 1% O_2_ after 7 days, because these conditions impaired NK cell function and phenotype most profoundly. Neither treatment was capable of driving increases in cell proliferation and numbers within the TME (Fig. 7, A to D). IL-15 (10 ng/ml dose) induced almost no proliferation in hypoxia, despite inducing greater proliferation in normoxia (Fig. 7, A and B). In contrast, eNK cells were capable of proliferating in hypoxia, but this came at a cost: Fewer cells survived of these proliferating cells (Fig. 7, A to C) resulting in similar NK cell numbers at the end of 7 days in hypoxia (Fig. 7D). In all contexts, we observed lower viability in severe hypoxia compared to normoxia, but since killing assays were adjusted for viable cells at the start of the assay, any deficits in killing would not arise solely from a decrease in viability under hypoxic conditions.

Examining cytotoxicity of K562 or Raji and rituximab revealed pbNK cells treated with IL-15 (10 ng/ml), and eNK cells were more cytolytic in severe hypoxia than pbNK cells treated with IL-15 (1 ng/ml), with eNK cells being the most cytolytic overall. Compared to their counterparts in normoxia, pbNK cells treated with IL-15 (10 ng/ml) showed no effect of oxygen on natural cytotoxicity, but ADCC was worse in severe hypoxia. In comparison, eNK cells showed no effect of oxygen on natural cytotoxicity, but their ADCC was superior in severe hypoxia compared to normoxia (Fig. 7, E to H). To determine the kinetics of these interventions within the setting of hypoxia, we evaluated cytolytic activity at three earlier time points, day 0 (baseline), day 1, and day 3 (fig. S14, A to E). For both natural cytotoxicity and ADCC of solid tumor lines, our findings indicated that high-dose IL-15 and NK cell expansion improved cytolysis relative to less activated pbNK cells at all time points. Cytolysis was maximal in hypoxia on day 1, after initial priming with IL-15, and there was a decline in all responses in severe hypoxia from day 1 to day 3. eNK cells always had superior cytotoxicity to pbNK cells treated with IL-15 (10 ng/ml) in severe hypoxia, but this was not true of normoxia. Together, these data highlight the complex interactions of NK cell source and activation with oxygen over time. A consistent conclusion was the strong function of eNK cells in severe hypoxia.

Modifying adoptively transferred cells to express or secrete intrinsic IL-15 is a clinical approach being taken with induced pluripotent stem cell–derived NK (iNK) cells (*65*) and cord blood–derived NK cells (*66*). To determine whether this intrinsic expression of IL-15 would achieve a similar benefit to exogenous IL-15 (10 ng/ml), we made use of iNK cells with or without a fusion protein of IL-15 receptor α and IL-15, which is tethered to the iNK cell membrane. These research lines of iNK cells were unmodified apart from the IL-15 fusion protein (IL-15FP). Low-dose IL-15 was added to support survival of all iNK cells and maintain consistency between groups but failed to induce proliferation in normoxia and hypoxia. iNK with IL-15FP proliferated in normoxia, but proliferation was impaired in severe hypoxia (fig. S15, A and B). Upon examining natural cytotoxicity of iNK cells with or without IL-15FP (fig. S15, C and D), we found similar results to pbNK cells treated with a high dose of IL-15, with the IL-15FP inducing better iNK cytotoxicity in both normoxic and hypoxic conditions. These data show that both exogenous IL-15 and intrinsic IL-15 enhance NK cell killing in severe hypoxia, but severe hypoxia limits proliferation in both cases.

### IL-15 prevents the loss of granzyme B, but proteomic deficits remain

To examine how IL-15 (10 ng/ml) improved NK cell function in hypoxia, we compared these cells by CyTOF to the previously analyzed pbNK cells treated with IL-15 (1 ng/ml; fig. S16). Many of the key proteins that had been down-regulated in severe hypoxia at the lower dose of cytokine remained low in severe hypoxia with the higher cytokine dose, including cytotoxic proteins (TRAIL and FasLigand) and transcription factors (Eomes). The cytotoxic protein granzyme B and transcription factor T-bet were notable in that they had been suppressed in hypoxia with low dose of IL-15 but were no longer suppressed relative to normoxia when NK cells were stimulated with high dose of IL-15. A drop in CD122/IL-2Rβ suggests that a higher dose of IL-15 may be ineffective in stimulating NK cells in hypoxia, because signaling is compromised by the lack of this component within the IL-15 receptor complex. Activation markers CD69 and CD25 remained high with high dose of IL-15 in hypoxia, suggesting that enhanced function of NK cells in hypoxia with IL-15 (10 ng/ml) was not a result of reversing this activated phenotype. Similar to what we saw previously with 1 ng/ml IL-15 (figs. S10A and S11A), separating these NK cells according to known developmental subsets revealed no change in subset frequencies when cultured in normoxia or hypoxia (fig. S10B) and similar patterns in protein abundance were seen across subsets compared to bulk NK cells (fig. S11B). There was maintenance of granzyme B in all subsets and relative increases in CD25 and CD69 in hypoxia in all subsets. An increased abundance of NKG2D and Eomes in hypoxia was present for adaptive pbNK cells treated with IL-15 (1 ng/ml). This was also apparent for pbNK cells treated with the higher dose of IL-15 for both the small adaptive population and larger CD57^+^ population. This suggests that CD57^+^ NK cells might not be as susceptible to suppression by hypoxia as less mature subsets. While stimulation of NK cells with high-dose IL-15 enhances cytotoxicity in severe hypoxia, there is still opportunity to enhance their function further.

### Preactivation with feeder cells retains key proteins in hypoxia

**Fig. 8.**
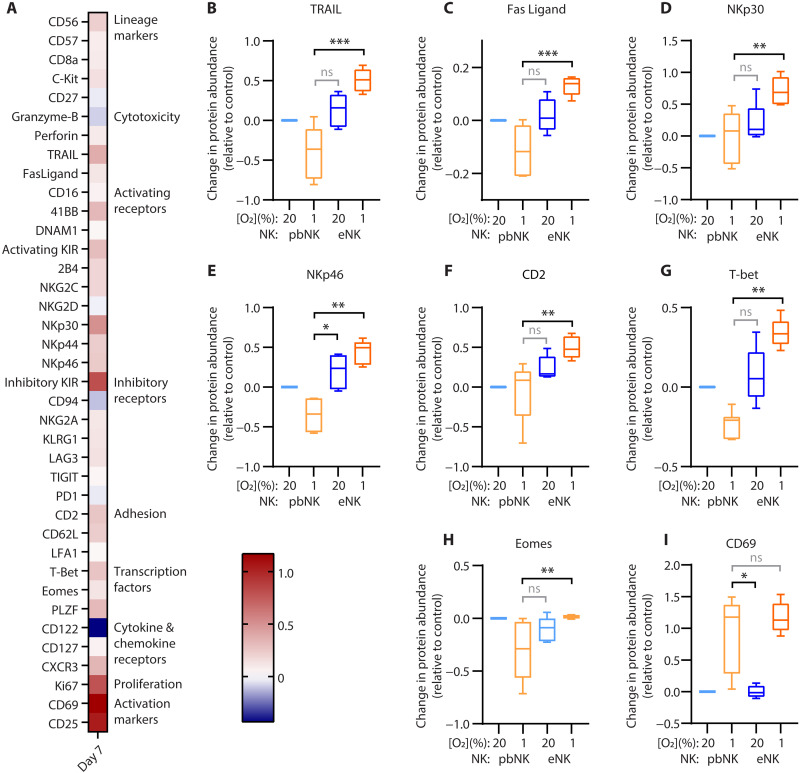
Preactivation of NK cells with IL-2 and feeder cells increases abundance of death receptors, activating receptors and transcription factors in severe hypoxia. NK cells expanded with feeder cells were thawed and incubated in normoxia (20% O_2_) or severe hypoxia (1% O_2_ + 2 psi) for 7 days. (**A**) A heatmap shows the relative change in protein abundance with 1% compared to 20% O_2_ by CyTOF analysis in Astrolabe Diagnostics. (**B** to **I**) Box (median and IQR) and whisker plots (minimum to maximum) show the relative abundance of key proteins as quantified by Astrolabe Diagnostics software, normalized to pbNK cells cultured in normoxia with IL-15 (1 ng/ml). Kruskal-Wallis test with Dunn’s multiple comparisons to 1% O2 pbNK cells (eNK cells; *n* = 5; pbNK cells are reproduced in fig. S16 for reference, *n* = 7); not significant, *P* > 0.05; *P ≤ 0.05; **P ≤ 0.01; and ***P ≤ 0.001

We asked what proteomic changes occurred in eNK cells when they were exposed to hypoxia, given that preactivation with feeder cells induced superior cytotoxicity in hypoxia, compared to IL-15 treatment. In contrast with pbNK cells, eNK cells had increased abundance of nearly all the key proteins in hypoxia relative to normoxia (Fig. 8). This included transcription factors (T-bet and, to a lesser extent, Eomes), activating receptors (NKp30, NKp46, and others), adhesion and costimulatory protein (CD2), and cytotoxic proteins (TRAIL, FasL, but not granzyme B). The CD56^bright^ population and adaptive populations were absent from our subset analysis of eNK cells, but we were able to analyze a continuum of more mature to less mature NK cells of which the majority were immature NKG2A^+^ KIR^−^ CD57^−^ cells (fig. S10C). There were no differences in the frequency of eNK cell subsets when cells were cultured in normoxia or hypoxia, and the subsets tended to follow the proteomic trends of the bulk population (fig. S11C). The IL-15 receptor CD122 was down on the immature populations in hypoxia but increased on CD57^+^ eNK cells in hypoxia. CD57^+^ eNK cells were a rare subset in our expansion method, but other expansion protocols that focus on generating adaptive cells might produce cells that respond more favorably to hypoxic environments. Emerging as a consistent feature of severely hypoxic NK cells regardless of the NK cell source and activation, markers of activation (CD25 and CD69) remained high across all eNK subsets.

Chemokine receptor expression is known to strongly differ between pbNK cells compared to eNK cells (*67*). The majority of chemokine receptors are not shared. CXCR3 is unusual in that it is found on both pbNK cells and eNK cells and on a number of NK cell lines (*68*). While CXCR3 was less abundant on pbNK cells in severe hypoxia compared to normoxia (fig. S16J), eNK cells had significantly more CXCR3 in severe hypoxia compared to pbNK, suggesting that they are more capable of trafficking in response to CXCR3 ligands in severe hypoxia.

### Feeder cell activation, but not IL-15 activation, stabilizes NAD^+^/NADH ratios

**Fig. 9.**
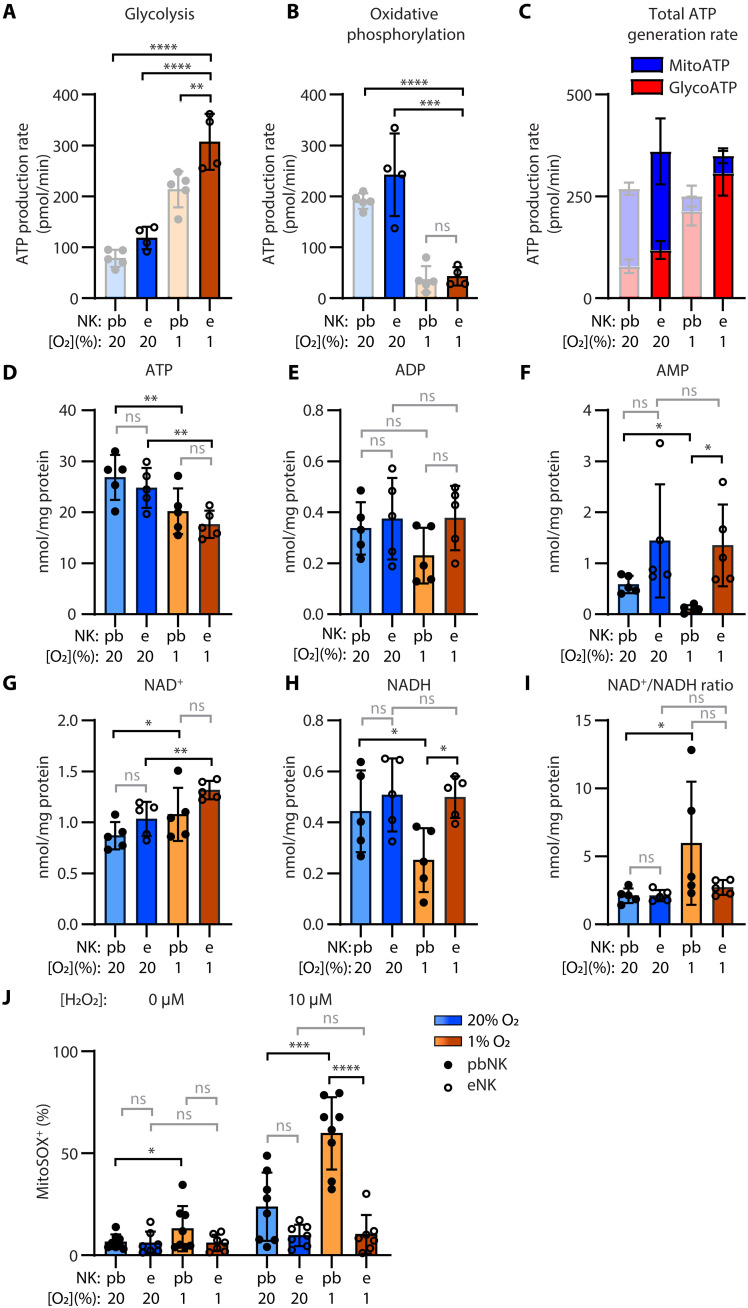
Preactivation of NK cells with feeders sustains AMP and NADH in severe hypoxia. Freshly enriched NK cells (pbNK) or thawed eNK cells were cultured in normoxia (20% O_2_) or severe hypoxia (1% O_2_ + 2 psi). After 7 days, the rate of ATP generation via glycolysis (glycoATP) (**A**) or oxidative phosphorylation (mitoATP) (**B**) was measured by Seahorse analysis; eNK (*n* = 4); pbNK (*n* = 5 of which five of seven were included in Fig. 6D; shown here opaque for reference only); analyzed by one-way ANOVA with Dunnett’s multiple comparisons test. The total rate of ATP generation from these two processes is plotted in (**C**). Bars represent the mean and SD. In (**D**) to (**I**), the energy status of cultured NK cells (as before) after 7 days in normoxia (20% O_2_) or severe hypoxia (1% O2 + 2 psi) was determined by analytical chemistry (*n* = 5); dots represent donors, and bars show the mean and SD. Analyzed by two-way ANOVA with Sidak’s multiple comparisons test. (**J**) ROS (mitoSOX^+^) were detected by flow cytometry within live NK cells (TOPRO-3^−^, singlets, CD56+, CD3^−^) when exposed to 0 or 10 μM hydrogen peroxide (H_2_O_2_); pbNK, *n* = 8; eNK, *n* = 7; bars show the mean and SD, analyzed by two-way ANOVA with Sidak’s multiple comparisons test. In all graphs, bars mark tests that were performed where *P* > 0.05 (not significant), *P ≤ 0.05, **P ≤ 0.01, ***P ≤ 0.001, and ****P ≤ 0.0001.

Having determined that eNK cells had superior cytotoxicity and protein abundance compared to pbNK cells in hypoxia, we asked whether the energy status and redox balance in these cells could be contributing to their enhanced function (Fig. 9). In terms of energy usage, pbNK cells treated with IL-15 (1 or 10 ng/ml; fig. S17A) relied on glycolysis in hypoxia, decreasing oxidative phosphorylation. eNK cells, in comparison to pbNK cells (1 ng/ml IL-15), increased their rate of ATP production by glycolysis even more in severe hypoxia (Fig. 9A) and similarly shut down oxidative phosphorylation (Fig. 9B), which led to a slightly higher rate of ATP production overall for eNK cells relative to pbNK cells in hypoxia (Fig. 9C). Both pbNK cells and eNK cells had less ATP in the cell under hypoxia than normoxia (Fig. 9, D to F), suggesting that enhanced cytotoxicity of eNK cells did not result from enhanced ATP generation leading to enhanced ATP availability. A deeper dive into metabolites revealed unchanged levels of ADP but very low levels of adenosine monophosphate (AMP) in pbNK cells (suggesting catabolism), but hypoxic eNK cells maintained their levels of AMP. Lower AMP relative to ATP within the cell (the energy charge; fig. S17B) is generally considered an indicator that ATP availability is not limiting. The high energy charge in hypoxic pbNK cells thus suggests that ATP is not limiting in these cells.

In pbNK cells and eNK cells, there was more nicotinamide adenine dinucleotide (NAD^+^) in hypoxic cells than normoxic cells (Fig. 9G). Glycolysis converts NAD^+^ to NADH (reduced form of NAD^+^), so NAD^+^ must be regenerated for glycolysis to proceed. This suggests that NAD^+^ levels are being actively regenerated in hypoxia-adapted cells. The lethality of LDHA inhibition in hypoxia also suggests that the regeneration of NAD^+^ by LDHA is critical for NK cell survival. In pbNK cells, higher NAD^+^ levels combined with lower availability of NADH led to a skewed NAD^+^/NADH ratio, but in eNK cells, NADH levels were maintained, and the ratios did not differ for eNK in hypoxia and normoxia (Fig. 9, G to I). These altered NADH levels were significantly higher for eNK cells compared to IL-15–treated pbNK cells (1 ng/ml) under hypoxia. pbNK cells treated with IL-15 followed a similar pattern regardless of treatment dose (fig. S17B). Other metabolites (acetyl coenzyme A and succinyl coenzyme A; fig. S17B) varied according to therapeutic intervention in hypoxia, suggesting that altered metabolic pathways in these cells extend beyond ATP availability and NAD^+^/NADH ratios.

It should be noted that the energy measurements described are based on the total amount of these molecules within the cells, but it is the amount of free NAD^+^ and NADH that has regulatory roles. To address whether the altered NAD^+^/NADH ratio correlated with oxidative stress, we challenged NK cells at the end of culture with increasing doses of peroxide (Fig. 9J and fig. S17C). Titrating in the dose of peroxide revealed that pbNK cells treated with IL-15 (1 or 10 ng/ml) under hypoxia were very sensitive to oxidative stress, with more ROS detected by mitoSOX staining than in eNK cells that were very resistant to oxidative stress, highlighting another difference of NK cells based on their activation conditions. This aligns with the NAD^+^/NADH balance being maintained in eNK cells but not pbNK cells. Thus, the redox balance within hypoxia-cultured eNK cells likely contributes to their improved function in the energetically demanding context of tumor cell cytotoxicity.

### Knocking out *HIF1A* fails to enhance cytotoxicity of eNK cells in hypoxia

Features that would improve the performance of adoptively transferred NK cells in solid tumors are of interest to the scientific community. Since HIF-1 signaling was up-regulated in hypoxia in pbNK cells over time (fig. S12C), we asked whether knocking out *HIF1A* would result in enhanced cytotoxicity of tumor cells in our experimental system. Translation of *HIF1A* is known to be driven by IL-15/IL-2 signaling in NK cells (*69*–*71*) so their history of activation with cytokines may affect their reliance on HIF-1α in hypoxic environments. We used a CRISPR-Cas9 system to knock out (KO) *HIF1A* on NK cells at an intermediate point of expansion (fig. S18A) and then proceeded to further expand the *HIF1A* KO cells. After 7 days in hypoxia, HIF-1α was detectable by flow cytometry in hypoxic wild-type (WT) cells, but the levels were significantly lower in KO cells, aligning with the staining for HIF-1α seen in normoxic cells (fig. S18, B and C). Harvesting the cells on day 7 revealed no differences in viability, proliferation, or yield in hypoxia between WT and KO cells (fig. S18, D to F), although there were differences in proliferation in normoxia, with KO cells proliferating less than WT cells (fig. S18E). *HIF1A* CRISPR KO did not induce substantial changes in either natural cytotoxicity or ADCC (fig. S18, G to J). Together, these data suggest that the expansion process primes eNK cells to function in hypoxia, not only removing their reliance on HIF-1α but also negating benefit of knocking out *HIF1A* in this setting.

### CD16-targeted delivery of IL-15 further augments NK cell function under hypoxia

**Fig. 10.**
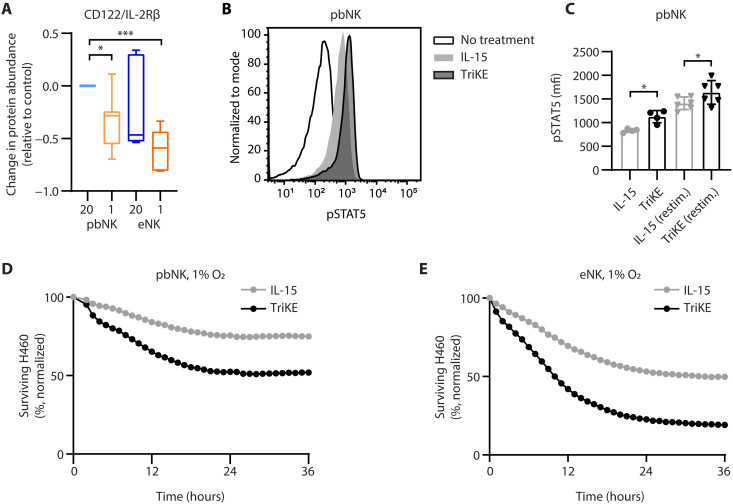
CD16-targeted delivery of IL-15 further improves function of primary and preactivated NK cells in severe hypoxia. (**A**) Freshly enriched NK cells (pbNK) or thawed eNK cells were cultured in normoxia (20% O_2_) or severe hypoxia (1% O_2_ + 2 psi) for 7 days and analyzed by CyTOF, as in Fig. 7. A box (median and IQR) and whisker plot (minimum to maximum) show the relative abundance of CD122/IL-2Rβ, normalized to pbNK cells cultured in normoxia with IL-15 (1 ng/ml), quantified by Astrolabe Diagnostics software. Mixed-effects analysis with Dunnett’s multiple comparisons test to 20% O_2_ pbNK cells (eNK cells; *n* = 5; pbNK cells are reproduced in fig. S16 for reference, *n* = 7). (**B**) During 7-day culture in hypoxia, pbNK cells were incubated with no cytokines (empty histogram), 3 nM mesothelin-targeting TriKE (dark histogram), or an equifunctional dose of IL-15 (light histogram). Representative histograms are shown of pSTAT5 staining assessed by flow cytometry on day 4 of culture, before restimulation with drugs. (**C**) The median fluorescence intensity of the pSTAT5 signal on day 4, before (*n* = 4) or after (*n* = 6) restimulation with TriKE or IL-15 is quantified for independent donors. Mixed effects analysis with Holm-Sidak’s multiple comparisons test; bars shown the mean and SD. (**D**) Primary or (**E**) expanded NK cells were incubated with TriKE (black) or an equifunctional dose of IL-15 (gray) during 7-day culture in hypoxia. After washing away the drugs, cytotoxicity analysis of NCI-H460 was performed as in Fig. 1, shown for one of three representative donors. In all graphs, *P ≤ 0.05 and ***P ≤ 0.001.

The NK cell activations tested (IL-15 and eNK) enhanced cytotoxicity in severe hypoxia, but there were opportunities to further enhance antitumor activity. The consistently low abundance of CD122/IL-2Rβ in severely hypoxic pbNK and eNK (Fig. 10A) suggested that IL-15 signaling may still be a limiting factor through this receptor. Our lab has previously described how targeting both CD16 and IL-15R signaling with tri-specific killer engager (TriKE) molecules drives robust proliferation of CD16^+^ NK cells in preference to CD16^−^ T cells (*72*). We hypothesized that CD16 targeting of the IL-15 moiety to hypoxic NK cells might overcome decreased abundance of CD122, enhancing signaling through the IL-15 receptor relative to IL-15. To test this, we measured the levels of phosphorylated signal transducer and activator of transcription 5 (STAT5) after an initial stimulation and upon restimulation with a TriKE or a dose of IL-15 (“equifunctional”) that drove equivalent activation/proliferation in CD16-null cells, as previously described (*73*). After 4 days in culture, TriKE-treated cells had greater levels of phosphorylated STAT5 at baseline and upon restimulation than IL-15–treated cells, indicating better downstream signaling through IL-15 (Fig. 10, B and C). In normoxia, restimulation with IL-15 or TriKE generated more phosphorylated STAT5 than in severe hypoxia. This suggests that the deficit in CD122 was impairing STAT5 signaling relative to normoxia, even in the presence of TriKE (fig. S19). The antitumor element of the TriKE used here targeted mesothelin (SS1 TriKE) (*73*) that can be found on the lung cancer cell line H460. To test whether this enhanced IL-15 signaling led to enhanced function, we incubated pbNK cells or eNK cells with the SS1 TriKE or an equifunctional dose of IL-15 for 7 days in severe hypoxia, in the absence of any tumor target. When these cells were washed and challenged with H460 at the end of the 7-day incubation, TriKE-pretreated cells showed enhanced cytotoxic activity against these mesothelin-expressing targets, compared to IL-15 pretreatment alone (Fig. 10, D and E). If TriKE was not included in the 7-day pretreatment, but instead added upon exposure to the H460 cells, then the differential effect was greatly diminished (fig. S20). This suggested that the benefit to the NK cells was provided by enhanced IL-15 signaling during hypoxia, not just because of the enhanced tumor targeting through CD16 that a TriKE provides. Treating with the SS1 TriKE during exposure to hypoxia and upon tumor challenge maximized both IL-15 delivery and tumor-targeting mechanisms to provide the greatest benefit relative to IL-15 alone (fig. S20). Overall, these data show that pbNK and eNK cell responsiveness to IL-15 is limited by decreased abundance of CD122. The result of targeting CD16 and IL-15 together is enhanced responsiveness to IL-15 signals and enhanced antitumor activity in hypoxia. Together, these data point to potent IL-15 signaling, effector protein abundance, and a stable redox balance as critical factors for optimal tumor control by NK cells in severe hypoxia.

## DISCUSSION

We have demonstrated that oxygen concentrations commonly found in arterial blood (12% O_2_) do not affect NK cell cytotoxicity or transcriptional programming, relative to normoxic controls (20% O_2_), but more severe hypoxia has profound effects on NK cell function. This changes our interpretation of how to study NK cell biology under conditions of physiologic relevance. While conventional incubators are acceptable in modeling the oxygen concentrations found in arterial blood, they do a poor job of replicating the challenging conditions found in the TME, where 1% O_2_ strongly inhibits NK cell function. In addition, there is a transcriptional imprint when NK cells are exposed to intermediate oxygen levels found in bone marrow (5% O_2_). This imprint does not result in altered killing by NK cells but may be relevant to NK cell therapy of myeloid leukemias (*74*), now in clinical trial testing by many groups (*11*, *22*, *75*–*77*). In contrast to hematologic malignancies, solid tumors are more heterogeneous, with great variation in oxygen availability in different tumor types and locations within a tumor mass. These experiments have demonstrated that as NK cells penetrate into the TME and experience greater oxygen restriction, they reprogram themselves to shut down the cell cycle, increase mitophagy, autophagy and endocytosis, and switch over to rely on glycolysis. Proteomic and mitochondrial changes seen in our system resemble NK cells extracted from hypoxic solid tumors (*28*, *62*). Transcriptionally, reanalysis of a glioblastoma scRNA-seq dataset looking at tumor-infiltrating NK cells [GSE147275 (*61*)] compared to blood NK cells [GSE130430 (*60*)] confirmed up-regulation of key genes relating to glycolysis, autophagy, endocytosis, and mitophagy. These data suggest that our results are clinically relevant and that oxygen deprivation has the potential to be a major driver of NK cell function in vivo.

We observed profound changes in NK cell metabolic regulation under hypoxic conditions. NK cells switch to rely on glycolysis with increased expression of LDHA. There was a decrease in mitochondrial metabolism, mitochondrial mass, and the net amount of ATP in cells, coupled with an increase in ROS. The rate of ATP production was similar in normoxic or severely hypoxic NK cells, suggesting that reduced ATP levels might result from greater energy demands in hypoxia. Among the pathways up-regulated in hypoxic pbNK cells, endocytosis is required in hypoxic epithelial cells to remove Na^+^/K^+^ adenosine triphosphatase from the plasma membrane to limit ATP expenditure (*78*). Thus, endocytosis and autophagy may be a necessary step for pbNK to acclimatize to hypoxia by preserving ATP turnover in the cell. NK cells that switch to rely on glycolysis in oxygenated environments (aerobic glycolysis) use this metabolic pathway to proliferate, kill, and produce cytokines as knocking out *LDHA* prevents these functions (*79*). In contrast, severely hypoxic NK cells rely on glycolysis but were only able to produce cytokines and died when LDHA was inhibited. Others have also shown that cytokine and chemokine production is independent of oxygen and glucose availability in short-term assays (*80*). In mice, deletion of *Cox10*, a component of complex IV that is required for oxidative phosphorylation, did not impair cytokine production at baseline (*81*), suggesting that cytokine production can be independent of oxygen. Nevertheless, the metabolic requirements for cytokine production vary with the kind of stimulation used (*42*, *79*, *81*). Given the complexities of hypoxia on immune cells, we need to carefully interpret results between studies as precise conditions seem to determine ultimate functional outcomes, and understanding these mechanisms is important to develop effective therapeutic strategies.

HIF-1α is regulated at many levels, but low oxygen is known to rescue HIF-1α protein from E3 ligase degradation. Downstream of IL-15/IL-2 receptor activation, STAT3, and the phosphoinositide 3-kinase (PI3K)/mammalian target of rapamycin (mTOR) pathway increase transcription and translation of *HIF1A* in human NK cells (*69*, *70*). Murine NK cells with a *HIF1A* KO had less ATP, more ROS, were less viable, and produced less IFN-γ when examined ex vivo without IL-2/IL-15 activation (*31*). In a different study, the same cells with strong IL-2 stimulation had better IFN-γ production than their WT counterparts (*29*). Thus, cytokine and receptor activation–induced PI3K/mTOR stimulation can alter the cellular response to HIF-1α. Our results show that NK cells activated by a combination of 4-1BBL, membrane-bound IL-21, IL-2, and irradiated K562 feeders resulted in highly functional NK cells in severe hypoxia, independent of HIF-1α. Using a feeder-free method of NK cell expansion (IL-2, IL-18, anti-NKp46, and anti-CD16), others have shown that a *HIF1A* KO in human NK cells gave a subtle improvement in cytotoxicity in hypoxia, so the context of priming and evaluation are critical to any benefit observed (*82*). While it is known that hypoxia response elements exist in the LDHA promoter (*83*) and HIF-1α mediates much of its metabolic regulation via LDHA and pyruvate dehydrogenase kinase 1 up-regulation (*84*), we conclude that the role of HIF-1α may be different for resting NK cells or those activated using various activation strategies.

IL-15 (either intrinsic or extrinsic) and the expansion process enhanced cytotoxicity, and our data suggest distinct molecular mechanisms underlying these strategies. pbNK and eNK cells lacked CD122 in hypoxia and pbNK cells phosphorylated STAT5 to a lesser extent in response to IL-15 after exposure to severe hypoxia. In IL-15–treated cells, this STAT5 signaling was presumably sufficient to prevent the observed loss of granzyme B, TRAIL, and T-bet, but several key factors were still decreased (Eomes and TRAIL). In contrast, eNK cells had higher amounts of a much wider range of critical factors (T-bet, Eomes, FasL, TRAIL, and activating receptors), and their ability to kill was not impaired. eNK responsiveness peaked early after thaw in both normoxia and hypoxia, but peak responsiveness for IL-15 varied by oxygen treatment, peaking early in hypoxia, but later in normoxia. This drop in responsiveness in hypoxia mirrors the temporal loss of CD122. While overexpression of CD122 might be expected to prevent this decline, it also risks exhausting NK cells through sustained IL-15 stimulation (*85*). If overexpression of CD122 was under the control of hypoxia-inducible factors, this might circumvent this issue. Metabolically, eNK cells were distinct from high-dose IL-15–treated NK cells, with a lower NAD^+^/NADH ratio, better maintenance of AMP, and greater resistance to ROS. This aligned with a previous study of NK cells in liver cancer and other studies in T cells showing interventions that improve mitochondrial function and alleviate ROS enhance antitumor responses (*28*, *35*, *86*). Overall, IL-15 alone boosts cytolytic capacity in hypoxia, but greater metabolic stability of eNK cells renders them more adept at functioning in severe hypoxia, similar to what others have observed (*66*).

The use of expanded NK cells as immunotherapy has grown over the years to include overnight activated pbNK cells, NK cell lines, and cord blood–derived cells as well as genetically modified NK cells from different sources including induced pluripotent stem cells (*87*–*89*). Preclinical data using NK cells expanded with K562 feeder cells have been published for a wide range of cancers including a few solid tumors (*77*, *90*). A phase 1 clinical trial of cord blood–derived, feeder-expanded CD19-chimeric antigen receptor NK cells infused into patients with relapsed/refractory lymphoid malignancies proved safe with promising clinical activity (*4*). This ex vivo expansion system was initially developed to boost cell numbers for NK cell–based cellular products because the K562 feeder cells enable log-phase growth of NK cells for many weeks without inducing senescence (*91*). The results from our study show that including an expansion step during the generation of NK cellular products prepares the cells to function better under hypoxia. Both 4-1BBL and IL-21 are known to contribute to this expansion process for therapeutic products (*91*, *92*) and have the potential to contribute to the metabolic benefit we observed. In T cells, short-term stimulation through 4-1BB can drive metabolic reprogramming to improve control of cancer in preclinical models (*93*). IL-21 is a pleiotropic cytokine that could itself be contributing to this metabolic rewiring alone or through synergy with IL-2/IL-15 stimulation and activating receptor stimulation via K562. Regardless of the contribution of each individual factor, the combined effect of this expansion process—one that is well-established within clinical trials at present—is to prime adoptive NK cells for therapeutic function in hypoxia.

While IL-15 and NK cell expansion mitigated the loss of function and improved NK cell killing under hypoxia, there are opportunities for further investigation. Many immunotherapies in development make use of different modalities of IL-15 (*7*). Our study highlights the importance of efficient delivery of IL-15 since CD122/IL-2Rβ is decreased by severe hypoxia. Systemic treatment with an IL-15 receptor superagonist (nogapendekin alfa inbakicept), recently US Food and Drug Administration approved for the treatment of bladder cancer, can enhance rejection of allogeneic NK cells by enhancing cytolytic T cell responses when dosed frequently (*76*). Therefore, targeted delivery of IL-15 to NK cells, cell-intrinsic IL-15 production, or careful dosing schema to avoid exhaustion or allogeneic depletion may be needed and require further investigation (*94*).

Our study highlights the importance of considering oxygen content of the environment when evaluating therapeutic potential. Immunotherapies have demonstrated precision and durability of responses in the clinic, but to broaden the applicability and success of NK cell therapies, we must build the suppressive elements of the TME into the design and testing of new immunotherapies. As NK cell immunotherapies are being tested in solid tumor settings, our data indicate that activation using targeted delivery of IL-15 and/or expansion of NK cells pose considerable advantages in terms of preserving cytolytic activity and mediating hypoxia resistance within inhospitable solid TMEs.

## MATERIALS AND METHODS

In depth descriptions of protocols are available as a collection at protocols.io: dx.doi.org/10.17504/protocols.io.5qpvo3y9xv4o/v1.

### Cell lines

The cell lines NCI-H460, LN229, HT29, Raji, K562, and P815 were obtained from American Type Culture Collection, and OVCAR8 was received from the National Cancer Institute (NCI). Cal33 were a gift from R. Garcia-Escudero [Centro de Investigaciones Energéticas, Medioambientales y Tecnológicas (CIEMAT), Spain], and K562 and Raji expressing Nuclight Red were received from Fate Therapeutics, California. Cal33, K562, and Raji were all authenticated by short tandem repeat typing. The K562, Raji, H460, OVCAR8, and HT29 cells were cultured in R10 media: Roswell Park Memorial Institute 1640 (Gibco, catalog no. 2240-089) + 10% fetal bovine serum (Gibco, catalog no. 26140079) + penicillin and streptomycin (100 U/ml; Gibco, catalog no. 15140122). LN229, Cal33, and P815 were cultured in D10 media: Dulbecco’s modified Eagle’s medium (DMEM; Corning, catalog no. MT10013CV) + 10% fetal bovine serum (Gibco, catalog no. 26140079) + penicillin and streptomycin (100 U/ml; Gibco, catalog no. 15140122). Nuclight Red levels were maintained with puromycin (1 μg/ml; Sigma-Aldrich, catalog no. P9620-10ML). All cells were cultured in humidified incubators at 37°C, set to 5% CO_2_. All cells were routinely tested for mycoplasma.

### Isolation and culture of immune cells

Deidentified human blood products were obtained from Memorial Blood Bank (Minneapolis, MN). Their use was approved by the University of Minnesota and National Marrow Donor Program institutional review board (ID9709M00134). Blood was processed to obtain PBMCs using density gradient Ficoll-Paque (GE Healthcare, catalog no. GE17-5442-03). For experiments with enriched NK cells (pbNK), PBMCs were processed fresh using the EasySep Human NK Cell Enrichment Kit (STEMCELL Technologies, catalog no. 19055). For experiments studying the interaction of NK cells with T cells, the EasySep Human CD3 Positive Selection Kit II (STEMCELL Technologies, catalog no. 17851) was used to enrich T cells for addition into wells with NK cells (1:1). All these preparations of NK cells were cultured in R10 media supplemented with IL-15 (1 ng/ml; R&D Systems) in a standard humidified incubator (20% O_2_, 5% CO_2_, 37°C, atmospheric pressure) or AVATAR System (Xcell Biosciences) incubator imitating blood (12% O_2_, 5% CO_2_, 37°C, + 2 psi), bone marrow (5% O_2_, 5% CO_2_, 37°C, + 0.6 psi), low pressure tumor (1% O_2_, 5% CO_2_, 37°C, + 0.3 psi), or high pressure tumor (1% O_2_, 5% CO_2_, 37°C, + 2 psi). We selected the AVATAR System, because, in our hands, it stabilized faster and gave more consistent results than other hypoxic chambers and because pressure was a variable factor in the experimental setup.

### Assessment of oxygen and pressure as biological variables

Two factors that can affect the severity of hypoxia are pressure and timing. Within tissues, oxygen availability is determined by partial pressure—a combination of the concentration of oxygen and the physical pressure the tissue experiences (*43*). Tumors can have high interstitial pressure, which may affect their growth and response to therapy (*44*–*46*). Thus, we considered pressure a relevant variable to consider in evaluating the function of NK cells in hypoxic environments.

Since oxygen and pressure were both varying in this model, we asked whether pressure had an effect on its own by comparing NK cell proliferation and cytotoxicity at 1% O_2_ at low or high pressure. CellTrace dye was used to track NK cell proliferation under these conditions over 7 days. This revealed no impact of pressure on the number of NK cells recovered at the end of 7 days and no impact on the proportion of cells dividing (fig. S1B). There were measurable increases in degranulation at higher pressure, when NK cells were challenged with tumor cells to evaluate their capacity for natural cytotoxicity and ADCC (fig. S1C). This suggested that the lower partial pressure of oxygen at lower pressure might be impairing cytotoxic granules release. However, this did not result in improved lysis of tumor cells by natural cytotoxicity or ADCC under high pressure compared to low (fig. S1D) as measured by live cell imaging of fluorescent tumor cells in the presence of an indicator of apoptosis. Figure 1 shows that, in these same assays, there were large differences in NK cell cytotoxicity and proliferation based on the different oxygen levels so this became the focus of how this research is presented.

For 7-day incubations, the media was changed on day 4. This procedure was selected because it avoided acidification of the media in 1% O_2_, and there were no significant differences in NK cell viability across oxygen conditions, as evaluated by flow cytometry annexin V^+^ and live/dead^+^ staining of NK cells among PBMCs on day 7 (fig. S1A). In comparison, under these same conditions, there were substantial differences in NK cell proliferation and cytotoxicity (Fig. 1). In later assays with higher *n* numbers, measurable differences in viability did emerge between 20% O_2_ and atmospheric pressure and 1% O_2_ + 2 psi (Fig. 7). These are discussed when they appear in the text. However, the size of the effect was always small compared to the impacts on proliferation and cytotoxicity, and so data are focused on proliferation and cytotoxicity as key readouts that are both essential processes for cancer immunotherapy.

### Models of therapeutic interventions—IL-15, expanded NK cells, and iNK cells

Where enriched NK cells were cultured in IL-15 (10 ng/ml; R&D Systems) as a therapeutic intervention strategy and cultured as above for 7 days, these assays were performed in parallel to the same donor treated with IL-15 (1 ng/ml), as the matched control. eNK cells were prepared according to (*95*) with the following slight alterations. NK cells were enriched from PBMCs as above. K562 membrane–bound IL-21–4-1BBL feeder cells were a gift of Fate Therapeutics and were maintained as previously described (*96*). Irradiated feeder cells and enriched NK cells were incubated at 2:1 ratio for the first week, and fresh irradiated feeders were added at a 1:1 ratio in the second week for a total of 14 days. The media was R10, supplemented with IL-2 (50 U/ml; Prometheus, catalog no. NDC 65483-116-07), which was refreshed every 2 to 3 days. In the second week, cells were cultured in G-rex vessels (Wilson Wolf, catalog no. 80660M). After 14 days, cells were frozen at >10 million/ml in 90% fetal bovine serum and 10% dimethyl sulfoxide. To assess their function in hypoxia, they were thawed and placed immediately into culture, as above. The eNK donors were not matched to the pbNK donors.

iNK cells were provided as frozen vials from Fate Therapeutics. An unmodified research line was provided alongside a research line with a single modification of the membrane-tethered fusion protein of IL-15 and IL-15 receptor α (IL-15FP) that is one of their available edits for their multiplex edited products. After thaw, freezing media was removed by centrifugation, and cells were cultured with IL-15 (1 ng/ml; R&D Systems) in B0 media: 60% DMEM (Corning, catalog no. 10-017-CV), 30% modified Ham’s F-12 (Corning, catalog no. 10-080-CV), 10% heat-inactivated human AB serum (Valley Biomedical Inc., catalog no. HP1022), penicillin/streptomycin (100 U/ml; Gibco, catalog no. 15140-122), 20 μM β2-mercaptoethanol (Sigma-Aldrich, catalog no. M7522), 50 μM ethanolamine (Sigma-Aldrich, catalog no. E0135), ascorbic acid (20 μg/ml; Sigma-Aldrich, catalog no. A4544), and sodium selenite (5 ng/ml; Sigma-Aldrich, catalog no. S5261) for up to 7 days with a media change on day 4.

### Proliferation assays

PBMCs (Fig. 1) or enriched NK cells (Fig. 7) were labeled with the CellTrace Violet Proliferation Kit (catalog no. C34557, Thermo Fisher Scientific), cultured in oxygen-specific conditions as described above, harvested on day 7, and analyzed by flow cytometry on an LSR II (BD Biosciences). Relative NK cell counts on day 7, from 300,000 cells (PBMCs or NK) on day 0, were determined by harvesting, staining, and resuspending the day 7 cells in a known volume (200 μl) that was run on the cytometer at constant speed for 60 s. Proliferation was assessed by dilution of the dye. NK cells were identified using LIVE/DEAD near infrared (NIR; catalog no. L34976, Thermo Fisher Scientific), PE-CY7 conjugated anti-CD56 (RRID:AB_604107, HCD56, BioLegend), and PE-CF594–conjugated anti-CD3 (RRID:AB_604107, UCHT1, BD Biosciences). When viability was being directly quantified, fluorescein isothiocyanate (FITC)–conjugated annexin V (BD 556419, BD Pharmingen) was included, and cells were stained according to the manufacturer’s protocol. NK cells were identified as LiveDead^−^/annexin V^−^/CD56^+^/CD3^−^. Flow cytometric analysis was performed using FlowJo software (Tree Star Inc.).

### Functional assays (degranulation and cytokine production)

Following 7 days of culture in different oxygen conditions, assessment of NK cell degranulation by flow cytometry was carried out in those same oxygen conditions in a 5-hour assay. Effector cells (enriched NK cells) were plated in a 2:1 effector to target ratio with tumor cells: K562 without drug or Raji cells with rituximab (10 μg/ml; Genentech). Following addition of the treatments and targets, cells were stained with FITC-conjugated anti-CD107a (RRID:AB_1186036, clone H4A3, BioLegend). One hour after the addition of anti-CD107a, cells were given Golgi Stop and Golgi Plug (catalog nos. 554724 and 555029, BD Biosciences) and incubated for a further 4 hours. At the end of the incubation, cells were stained with the Live/Dead Fixable Aqua Staining Kit (catalog no. L-34966, Thermo Fisher Scientific), PE-CY7–conjugated anti-CD56 (RRID:AB_604107, HCD56, BioLegend), and PE-CF594–conjugated anti-CD3 (RRID:AB_11153674, UCHT1, BD Biosciences) and fixed [2% paraformaldehyde in phosphate-buffered saline (PBS)]. Cells were permeabilized with Triton X-100 and then stained with BV650-conjugated IFN-γ (RRID:AB_2563608, 4S.B3, BioLegend). The appearance of CD107a at the cell surface and build-up of IFN-γ within NK cells (live, CD56^+^ CD3^−^) were evaluated by flow cytometry on an LSR II (BD Biosciences).

A variant of this assay was used to examine degranulation and cytokine production, segregated by cell division. NK cells or PBMCs were labeled with the CellTrace Violet Proliferation Kit (catalog no. C34557, Thermo Fisher Scientific) as for a proliferation assay and then cultured for 7 days. After 7 days, these cells were challenged in a redirected lysis assay where Fc receptor–bearing P815 cells cross-link antibodies directed against NK cell receptors (anti-CD16 clone 3G8, BD Biosciences, catalog no. 550383; anti-NKp30 clone P30-15 LEAF, BioLegend, catalog no. 325204) or isotype control antibody (mouse IgG1 κ clone MOPC-21 LEAF, BioLegend, catalog no. 400124). These cells were stained, and responses were measured as above except with live/dead NIR (catalog no. L34976, Thermo Fisher Scientific) in place of live/dead aqua.

### Cytotoxicity by time-lapse microscopy (IncuCyte)

K562 and Raji cells were labeled using the CellTrace Far Red Proliferation Kit (catalog no. C34564, Thermo Fisher Scientific) or tracked through expression of Nuclight Red. Enriched NK cells were cultured in various oxygen conditions for 7 days (or 3 days where indicated) before challenging them with these fluorescent targets cells (K562 or Raji with rituximab at 10 μg/ml) in incubators capable of time-lapse microscopy (IncuCyte S3 and IncuCyte Zoom, Sartorius Inc.; humidified, 20% O_2_ 5% CO_2_, 37°C) for up to 48 hours. Death of tumor targets was tracked using Caspase-3/7 Green Apoptosis Assay reagent (catalog no. 4440, Essen BioScience). Images were taken every 30 min. A graph was created representing the percentage of live K562 and Raji targets (CellTrace Far Red^+^, Caspase-3/7^−^) normalized to the growth of target cells alone, and the number of live target cells in that well before NK cells was added (0 hours).

For experiments involving the mesothelin-targeted TriKE (cam1615SS1), the TriKE was added either during the 7-day exposure to different oxygen conditions; at the end of the 7 days, during the live cell cytotoxicity assay performed in 20% O_2_, 5% CO_2_; or both—as indicated. The TriKE was compared to an equifunctional dose of IL-15, calculated as that required to induce the same extent of proliferation in NK-92 cells, which lack CD16 and are dependent on IL-15 or IL-2 for survival, as described by Kennedy *et al.* (*73*). To match the low dose of cytokine used throughout culture, during the 7-day culture, the dose was set to IL-15 (1 ng/ml) or an equifunctional dose of the TriKE (3.4 nM). When TriKE was added at the end of the culture period, the dose was set to 30 nM TriKE, 0.67 nM IL-15 as this had previously been shown to maximize cytotoxicity in this assay (*73*).

### Cytotoxicity of adherent cells by impedance (xCELLigence)

This assay was adopted in addition to the live cell imaging assay because it allows efficient readout of adherent tumor cell survival without the need for a fluorescent reporter, so it can be used to evaluate a wide range of different tumor types.

Briefly, enriched NK cells were cultured in various oxygen and pressure conditions described above for up to 7 days before challenging them with target cells (HT29, LN229, Cal33, or OVCAR8) with or without cetuximab in incubators capable of impedance-based cytotoxicity monitoring (xCELLigence RTCA, Agilent Technologies Inc.; humidified, 20% O_2_ 5% CO_2_, 37°C). Seeding cell density and dose of cetuximab (0.1 μg/ml for cetuximab-sensitive Cal33 or 1 μg/ml for the other lines) were determined for optimal assay sensitivity to tumor survival while limiting tumor-intrinsic effects of cetuximab. Cal33 and OVCAR8 cells were seeded at 12,500 cells per well; LN229 cells were seeded at 25,000 cells per well; HT29 were seeded at 50,000 cells per well onto a 96-well gold-coated plate. Impedance readings were taken every 15 min for 24 hours. NK cells and drugs were added at the 24-hour mark, and impedance readings continued for 60 hours. Impedance measurements were used to infer adherent tumor cell survival at each time point. Cytolysis was estimated within xCELLigence software (the impedance of treatment wells, normalized to the impedance reading when NK cells were added and normalized to the impedance of wells containing tumor cells alone) and inverted to obtain tumor survival graphs. Graphs are plotted from the time of NK cell addition.

### CyTOF (Cytobank and Astrolabe)

Enriched NK cells and expanded NK cells cultured for 1, 3, or 7 days in various oxygen conditions were stained with Cisplatin (catalog no. 201064, Fluidigm) followed by barcoding using the Cell-ID 20-Plex Pd Barcoding Kit (catalog no. 201060, Fluidigm). Barcoded samples were combined into a single 5-ml polystyrene U-bottom tube and incubated in the surface marker antibody cocktail for 30 min at 4°C. Cells were then fixed using 2% paraformaldehyde, for 20 min. Cells were permeabilized with 0.1% Triton X-100 for 5 min at room temperature, followed by incubation with the intracellular antibody cocktail for 30 min at 4°C (listed in table S1). Stained cells were incubated overnight with Cell-ID Intercalator (catalog no. 201192A, Fluidigm). The following morning, cells were washed with Maxpar PBS (catalog no. 201058, Fluidigm). Samples were run on a time of flight mass cytometer (CyTOF 2, Fluidigm). ViSNEs were generated of live, single NK cells (cisplatin^−^, Cell-ID Intercalator single cells, CD45^+^, CD56^+^, CD3^−^) using Cytobank software (Cytobank Inc., RRID:SCR_014043), where each plot was formed from data concatenated from three donors, organized on the basis of CD16, CD56, CD57, granzyme B, Ki67, NKG2A, and perforin. Differential expression analysis was performed in Astrolabe Diagnostics (https://astrolabediagnostics.com/) where NK cells were considered to be CD56^+^ CD3^−^ within live (cisplatin^−^) single cells (Cell ID Intercalator).

NK cell subsets were evaluated in Astrolabe Diagnostics software using the following definitions: the CD56^bright^ population (CD62L^+^, CD16^−^, CD57^−^, KIR^−^), CD56^dim^ immature population (CD62L^−^ CD57^−^ KIR^−^ NKG2A^+^), CD56^dim^ mature population (CD62L^−^ CD57^−^ KIR^+^ NKG2A^−^), late-stage cells (CD57^+^ NKG2C^−^), and adaptive cells (CD57^+^ NKG2C^+^). The developmental subset definitions are not generally applied to NK cells activated by feeder cells, but KIR and NKG2A can dictate functional status (*57*), and CD57^+^ cells are known to have less proliferative potential (*97*), so there is value in assessing the phenotypes of these populations. We acknowledge that some populations, such as the CD56^bright^ NK cells, were likely underestimated in these panels, for example, due to CD62L clipping. We included an “unassigned” cell population in plots to account for cells which fell outside these definitions (e.g., CD62L^+^ CD16^+^ cells).

### RNA sequencing

Briefly, NK cells cultured under different conditions were harvested, total RNA was extracted by the RNeasy Plus mini kit (Qiagen, Valencia, CA, USA), and RNA quality was evaluated by analysis of the RNA integrity number determined using a 2100 bioanalyzer (Agilent RNA 6000 Nano Kit, Agilent Technologies, Santa Clara, CA, USA). Total RNA (0.5 μg) was used for library prep with the TruSeq Stranded mRNA Library Prep Kit (Illumina) according to the manufacturer’s instructions. Validation of the library preparations was performed on an Agilent Bioanalyzer using the high sensitivity DNA kit (5067-4626, Agilent Technologies). Libraries were sequenced using the HiSeq sequencing system with a 2 × 150 bp paired-end read run (Illumina, San Diego, CA) by Novogene Corporation Inc. (Sacramento, CA). The overall quality of sequencing reads was evaluated using FastQC (v.0.11.9). Sequence alignments to the reference human genome (GRCh38) were performed using HISAT2 (v2.0.4). Read count was generated using featureCounts, differential expression analysis between normoxia and different O_2_ groups was performed using DESeq2, and genes were considered significantly differentially expressed when the absolute log_2_ fold change was >1 and *P*adj was <0.05. Gene Ontology and Kyoto Encyclopedia of Genes and Genomes (KEGG) pathway enrichment analysis were done using the g:Profiler—a web server for functional enrichment analysis (https://biit.cs.ut.ee/gprofiler/). Data are deposited as GSE269629.

### scRNA-seq analysis

The peripheral blood dataset GSE130430 and excised glioma dataset GSE147275 were downloaded from the Gene Expression Omnibus database and reanalyzed by a commercial pipeline built by LatchBio Inc. (https://latch.bio/) with embedded Seurat package (v2.3.1). In the quality control step, we filtered out the cells that expressed <200 genes or >2500 genes and removed cells with >5% mitochondrial transcripts content. These two datasets were then merged for differential gene expression analysis. Briefly, gene expression values for each cell were log-normalized and scaled on the basis of the number of Unique Molecular Identifiers in each cell and the cell mitochondrial transcript content. For differential gene expression, we used Wilcoxon rank-sum test and only selected the genes with adjusted *P* value of <0.05. A machine learning model using a ridge classifier was built on the scRNA-seq differentially expressed genes (DEGs) to evaluate the accuracy [i.e., (true positives + true negatives)/all] of separating NK cells from gliomas (considered hypoxic) from NK cells from blood (considered normoxic). We conducted two tests: using all DEGs identified from scRNA-seq analysis and using the DEGs that were only present in the top 50 DEGs identified from bulk RNA-seq analysis of NK cells exposed to hypoxia or normoxia in vitro. As a control test, we randomly chose six DEGs from all scRNA-seq DEGs and repeated them three times. For each classification test, we ran 10-fold cross-validation tests, and the error bar represents the SD from these 10 validation tests.

### Oxidative phosphorylation and glycolytic rate assays

To assess metabolic activity, enriched NK cells were harvested after 7 days of culture and resuspended in Seahorse XF Assay Medium (Agilent Technologies). Plates were precoated with 0.01% poly-l-lysine solution (catalog no. P4707, 50 ml; MilliporeSigma) according to the manufacturer’s instructions, and 500,000 NK cells per well were immobilized in an even monolayer. The extracellular acidification rate and the oxygen consumption rate were measured (picomoles per minute) in real time on an XFe96 Extracellular Flux Analyzer (Agilent Technologies) after addition of drugs from the Mito Stress Test kit [1 μM oligomycin, then 1 μM carbonyl cyanide p-trifluoromethoxyphenylhydrazone (FCCP), and then 0.5 μM rotenone/antimycin A; catalog no. 103015-100], the glycolytic rate assay kit (0.5 μM rotenone/antimycin A and then 50 mM 2-deoxy-D-glucose; catalog no. 103344-100), and the ATP Rate Assay kit (1 μM oligomycin and then 0.5 μM rotenone/antimycin A; catalog no. 103592-100; all Agilent Technologies). Oxygen consumption rates are reported from the Mito stress test and ATP rate assay; extracellular acidification rate is reported from the glycolytic rate assay and ATP rate assay.

### ROS detection and ATP abundance

To assess levels of ROS, 500,000 enriched NK cells per condition were stained with the 5 μM MitoSOX Red Mitochondrial Superoxide Indicator (catalog no. M36008, Thermo Fisher Scientific). Live cells were identified by exclusion of TO-PRO-3 Iodide (catalog no. T3605, Thermo Fisher Scientific). Cells were kept on ice and evaluated on a LSR II flow cytometer (BD Biosciences) immediately. The levels of ATP in enriched NK cells were evaluated using the ATP Bioluminescence Assay Kit HS II (catalog no. 11699709001, Sigma-Aldrich) according to the manufacturer’s instructions. For this assay, 100,000 enriched NK cells were measured in triplicate per condition.

### Quantification of mitochondrial surface area and volume

For visualization of mitochondria in NK cells, chamber slides (μ-Slide eight-well polymer bottom 1.5, Ibidi, catalog no. 80826) were initially coated with fibronectin overnight at 4°C. After 7 days in 20 or 1% oxygen, NK cells were immobilized on the slides and stained with 200 nM MitoTracker deep red FM (Thermo Fisher Scientific, catalog no. M22426) for 30 min at 37°C. After washing, cells were then fixed with 4% paraformaldehyde (Electron Microscopy Sciences, catalog no. 15710) and permeabilized with 0.1% Triton X-100/PBS (Sigma-Aldrich, catalog no. 100261-4890) before a 4′,6-diamidino-2-phenylindole counterstain was added to visualize the nucleus (NucBlue fixed cell stain ReadyProbes; Thermo Fisher Scientific, catalog no. R37606). NK cells were imaged immediately after staining on a Nikon A1Rsi HD confocal microscope, using 60× 1.27 numerical aperture water immersion objective with 405- and 640-nm laser excitation, detected using two GaAsP detectors and a transmitted light detector. Z-stack images were denoised in Nikon Elements using a Fourier transform. The mitochondrial structure was modeled as three-dimensional spots within Imaris software, and the dimensions of these spots were quantified for mitochondrial surface area and volume.

### pSTAT5 flow cytometry

Flow cytometry was used to examine the levels of STAT5 phosphorylation at Y694. After 4 days in culture, NK cells were restimulated or not with 3 nM SS1 TriKE or an equifunctional dose of IL-15 (0.2 nM) for 30 min in the same oxygen conditions that they had been cultured in. See live cell cytotoxicity protocol for explanation of equifunctional IL-15 dose. Cells were fixed in prewarmed BD Phosflow Fix Buffer I (catalog no. 557870; contains 4.2% paraformaldehyde) for 10 min. Cells were washed with flow buffer: 1× PBS, 0.5% human antibody serum (catalog no. HP-1022HI, Valley Biomedical), 2.5 mM EDTA; and then permeabilized using ice-cold BD Phosflow Perm Buffer III (catalog no. 558050; contains 87% methanol). Cells were stained with Alexa Fluor 647–conjugated anti-STAT5 pY694 antibody (BD Biosciences, catalog no. 612599, RRID:AB_399882) for 30 min at 4°C. Levels were evaluated using a BD LSR II flow cytometer.

### Statistical analysis

Statistical analysis was performed in GraphPad Prism, unless otherwise indicated. Details of statistical tests are given in figure legends. Data were tested for normality with the Shapiro-Wilk test. Where two variables appear in panel, e.g., time and oxygen or time and tumor target cells, a two-way analysis of variance (ANOVA) was first performed with repeated measures if donors appeared across time points and conditions. If the variables of interest were changing (*P* ≤ 0.05) according to the two-way ANOVA, then post hoc tests were performed as recommended by GraphPad Prism software. Care was taken to avoid unnecessary multiple testing and focus on the comparisons of interest. To differentiate between a post hoc test not being performed or returning a nonsignificant result (*P* > 0.05), we have marked the latter with gray bars on appropriate panels. Where data were missing at random, e.g., an autofocus failure on a time point in live cell imaging, a mixed-effects model was performed in preference to a two-way ANOVA.

## Supplementary Materials

### This PDF file includes:

Supplementary Text Figs. S1 to S20 Table S1 References
