# Supplementary material for: Metabolic programs drive function of therapeutic NK cells in hypoxic tumor environments
Source: Sci Adv. 2024 Oct 30;10(44):eadn1849. doi: 10.1126/sciadv.adn1849 (PMC11524192; doi:10.1126/sciadv.adn1849)
Supplement: Supplementary file 1 — Supplementary Text Figs. S1 to S20 Table S1 References [file sciadv.adn1849_sm.pdf]

Science Advances
AAAS

Supplementary Materials for

Metabolic programs drive function of therapeutic NK cells in hypoxic tumor environments

Philippa R. Kennedy *et al.*

Corresponding author: Martin Felices, mfelices@umn.edu

Sci. Adv. **10**, eadn1849 (2024)
DOI: 10.1126/sciadv.adn1849

This PDF file includes:

Supplementary Text
Figs. S1 to S20
Table S1
References

Supplementary Text

Conversion of oxygen percentages to partial pressures

For reference, calculated partial pressures of oxygen in the respective incubators appear in the table below. These were calculated as follows:

Partial pressure of oxygen = (atmospheric pressure + additional pressure - saturated water vapor pressure) * oxygen fraction.

Conversion of oxygen percentages to partial pressures

Incubator setting	Partial pressures of oxygen (mmHg)
20% O_2	142
12% O_2 + 2 psi	98
5% O_2 + 0.6 psi	37
1% O_2 + 0.3 psi	7
1% O_2 + 2 psi	8

Useful pressure conversions:
Atmospheric pressure = 760 mmHg / 14. 7 psi
Saturated water vapor pressure at 37°C = 47.1 mmHg / 0.91 psi
Systolic/diastolic blood pressure (above atmospheric) = 120/80 mmHg or 2.3/1.5 psi
Capillary pressure = 10-20 mmHg / 0.19-0.39 psi (*99*)
Bone marrow pressure = approximately 25% of blood pressure = 30 mmHg / 0.6 psi (*100*)
2 psi = 103 mmHg
0.6 psi = 31 mmHg
0.3 psi = 16 mmHg

Adhesion assay

Protocols.io: dx.doi.org/10.17504/protocols.io.dm6gpj111gzp/v1

To assess how rapidly and stably NK cells from different culture conditions form synapses with K562 or Raji with rituximab (10µg/mL, Genentech), we applied a flow based assay where NK cells cultured in different oxygen conditions for 7 days were incubated for 0, 10, 20 or 30 min in a water bath at 37 °C; before fixation in 2% paraformaldehyde. The number of NK cells (CellTrace Violet labeled) forming conjugates with target cells (Nuclight Red fluorescent) as a proportion of all NK cells was then determined by flow cytometry.

Conjugate assay

Protocols.io:dx.doi.org/10.17504/protocols.io.5qpvordr9v4o/v1

NK cell conjugates with tumor cells were visualized on a Nikon A1Rsi HD confocal microscope, using 60x 1.4 NA oil immersion objective with 405 nm, 488 nm, 561 nm, 640 nm laser excitation, detected using four PMT detectors, two of them GaAsP detectors, and a transmitted

light detector. To prepare the cells, NK cells and K562 were co-mixed in V-bottom 96 well plate wells and incubated for 5 min at 37°C 5% CO_2, before being fixed with 4% paraformaldehyde/PBS (Electron Microscopy Sciences, Cat. 15710) for 30 min at room temperature. Cells were permeabilized in 0.01% Triton X/PBS (Sigma Aldrich, Cat. 100261-4890) for 5 min at room temperature. Cells were washed with PBS, then incubated for 1 h at room temperature in blocking solution: 3% bovine serum albumin (cat. A9430, Sigma), 1% human AB serum (cat. HP-1022HI, Valley biomedical) in PBS. Primary antibodies against pericentrin (0.5 µg/ml; RRID:AB_304461, rabbit IgG, Abcam, Cat. ab4448), LFA-1 (1µg/ml; RRID:AB_10662540, clone TS2/4, mouse IgG1, Biolegend Cat. 350602) and AlexaFluor488-conjugated antibody against perforin (1.25 µg/ml; RRID:AB_493252, clone δG9, mouse IgG2b, Biolegend, Cat. 308108) were incubated in blocking solution for 1 h at room temperature. Cells were washed again in PBS, then incubated with a blocking solution containing AlexaFluor647plus conjugated anti-rabbit antibody (1µg/ml; RRID:AB_2633282, ThermoFisher Scientific, Cat. A32733), AlexaFluor568 conjugated goat anti-mouse IgG1 (1µg/ml; RRID:AB_2535766, ThermoFisher Scientific, Cat. A-21124) and DAPI (NucBlue fixed cell stain ReadyProbes, ThermoFisher Scientific, Cat. R37606) for 1 h at room temperature. Cells were washed and post-fixed with 4% paraformaldehyde/PBS for 5 min at room temperature. Cells were mounted onto coverslips with Dako fluorescent mounting media (ThermoFisher Scientific, Cat. S3023) and allowed to cure at room temperature overnight. Slides were then stored at 4°C.

When imaging, conjugates were identified in the transmitted light channel and a z-slice through the contact point between the tumor cell and NK cell was obtained at the plane containing the microtubule organizing center (MTOC) of the NK cell. Images were analyzed using ImageJ (RRID:SCR_003070). The mean intensity of LFA-1 was measured for the synapse and the back of the cell using the line tool in order to calculate a ratio for LFA-1 enrichment at the synapse. The shortest distance between the MTOC and the synapse was measured and normalized to the distance between the synapse and the back of the cell for the z-slice where the MTOC appeared. Perforin granules were qualitatively scored 1 (majority of granules are gathered at the MTOC) or 0 (granules are dispersed throughout the cell). These values were then aggregated for all conjugates within a given condition.

CRISPR KO of HIF1α in expanded NK cells

a. Overview

Efficient CRISPR KO requires activation of NK cells, so we expanded NK cells with irradiated feeder cells (as described in Main methods section 2). We paused this expansion process on day 7 of expansion and performed the CRISPR KO described below. We allowed the cells to recover for 3 days in R10 media at 37 °C 20% O_2 5% CO_2 before evaluating them for indel efficiency by inference of CRISPR edits (ICE) analysis (Synthego). To get sufficient numbers of cells for our assay we then continued the expansion with irradiated feeder cells for a further 7 days, before freezing them following procedures described for eNK cells. To analyze their response to different culture conditions these eNK cells were thawed and cultured in the indicated oxygen and pressure conditions for 7 days and then evaluated by flow cytometry for HIF1α abundance, viability and proliferation and by impedance for cytotoxicity.

b. CRISPR

The chemically-synthesized sgRNA and Cas9 electroporation enhancer (Integrated DNA Technologies) was reconstituted in TE buffer (10 mM Tris, 0.1 mM EDTA, pH = 7.5) at concentration of 120 μM and 100 μM.

HIF1A guide sequence: CCATCAGCTATTTGCGTGTG.

For Cas9 RNP assembly, 1 μL sgRNA was mixed with 1 μL of Cas9 electroporation enhancer, followed by addition of 1 μL of 61 μM recombinant Cas9 protein (Integrated DNA Technologies) using slowly swirly pipetting. The mixture was allowed to form Cas9 RNP by incubation at 37 °C for 15 min. The assembled Cas9 RNP was stored at room-temperature until electroporation. For electroporation, 1 million Day 7 feeder-expanded NK cell was centrifuged at 400 x g for 5 min and the cell pellet was resuspended in 19 μL of P3 electroporation buffer (Lonza). The cell suspension was then mixed with assembled Cas9 RNP and transferred into 16-well nucleofection strip (Lonza). The electroporation was conducted on 4D Nucleofector (Lonza) using CM-137 pulse code. Immediately after electroporation, 80 μL of pre-warmed R10 medium was added into electroporation reaction and the 16-well strip was incubated at 37 °C for 20 min. After 20 min incubation, the NK cells were transferred into a 12-well plate pre-filled with 900 μL of R10 medium and cultured for 72 hours. Seventy-two hours later, 100,000 NK cells were collected by centrifuge at 400 x g for 5 min and resuspended in 35 μL QuickExtraction solution (LGC Biosearch Technologies), followed by incubation at 65°C for 6 min, 98°C for 2 min. The extracted genomic DNA was stored at -20°C until PCR amplification.

The rest of the NK cells were then used for a further 7 day expansion and downstream analysis. For PCR reaction, HIF1A genomic sequence was amplified from 100 ng of genomic DNA using Platinum™ SuperFi II Green PCR Master Mix as per manufacturer instructions.

Forward primer: CATGGCATCTTCTAATCCTTCTGTG.
Reverse primer: GCCAGTATCTTATTCCTGACTAAAG.

The amplified product was confirmed by 2% TAE agarose gel electrophoresis and purified using QIAquick PCR Purification Kit (Qiagen, Cat. 28104). The purified product was then resolved by Sanger Sequencing. The KO efficiency was analyzed by ICE analysis (Synthego).

HIF-1α flow cytometry

After 7 days in the indicated oxygen and pressure conditions, NK cells were washed in ice cold flow buffer (1% heat inactivated human AB serum, 0.5 mM EDTA in PBS) and then stained with live/dead NIR stain (Thermo Fisher, Cat. No: L34976; 1/1000) for 15 min at 4ºC. Cells were then fixed in 2% paraformaldhyde/PBS for 10 min at room temperature, permeabilized in 0.1% Triton X/PBS for 5 min at room temperature, stained with a primary antibody (clone: HIF1α67, Abcam, Cat. ab1-100ug; 1/100 dilution) followed by a secondary stain (highly cross-absorbed goat anti-mouse alexa fluor 488, Thermo Fisher, Cat. A-11029; 1/2000 dilution) with wash steps (450*g* for 5 min) in flow buffer in between. Cells were run on a FACSymphony A3 (BD Biosciences) and analyzed in FlowJo software.

Quantification of NK cell energy status

For normalization purposes, at the end of the 7 day culture, 4 million NK cells were frozen and lysed in 200 µL of protein lysis buffer. The protein concentration was calculated based on bicinchoninic acid analysis (BCA). In parallel, another 4 million frozen NK cells were homogenized using one time use pestels in 40 µL of 0.4 M perchloric acid, 0.5 mM EGTA extraction solution containing [$^{13}C_{10}$, $^{15}N_5$]ATP sodium salt (100 mM), [$^{13}C_{10}$, $^{15}N_5$]AMP sodium salt (100 mM), [1,2-$^{13}C_2$]acetyl-CoA lithium salt (5 mM), and [1,2,3-$^{13}C_3$] malonyl-CoA lithium salt (5 mM) purchased from Sigma. After incubation on ice for 10 min, samples were centrifuged at 15,000x*g* for 15 min at 4ºC. The resulting supernatants were neutralized with freshly prepared 0.5 M K_2CO_3, vortexed, and centrifuged at 15,000x*g* for 30 min at 4ºC. Final extracts were then analyzed by LC-MS/MS as previously described, with modifications (*101,102*). Briefly, analysis of energy metabolites was performed using a Vanquish LC system. Separation was achieved on a reverse-phase C18 column (Waters Xbridge, 150 x 2.1 mm, 3.5 µm) using the following mobile phases: A) 95% water/5% methanol with 4 mM dibutylammonium acetate (DBAA, Millipore Sigma) and B) 25% water/75% acetonitrile with 4 mM DBAA. The ion-pairing mobile phase additive DBAA provided adequate chromatographic separation. Samples were separated using the following binary gradients: 0-80% B for 8 min, 80-100% B for 5 min, 100% B for 3 min, and 100-0% B for 5 min. The flow rate was 100 µL/min, with a diverter valve used for the first 2 min to minimize matrix components entering the MS. The column was maintained at 30ºC and the injection volume was 2 µL. The LC system was hyphenated to a Thermo Q Exactive Plus MS equipped with heated electrospray ionization. The MS was operated in positive ionization mode with PRM mode used for quantitatively targeted analysis. The isolation window was set to m/z 1.0, resolution was 17,500, and collision energy 35 (arbitrary units). Retention times and m/z transitions for each metabolite are detailed in the Table below.

MS parameters for energy metabolite quantification

Analyte	Retention Time (min)	Molecular ion	Precursor-product transition	Precursor-product transition of Internal Standards
ATP	7.7	[ATP + DBAA + H]$^+$	637.1548→136.0623	[$^{13}C_{10}$, $^{15}N_5$] ATP 652.1735→146.0642
ADP	7.3	[ADP + DBAA + H]$^+$	557.1885→136.0623	
NAD$^+$	5.1	[NAD$^+$ + H]$^+$	664.1164→136.0623	
ADPR (NADH)	6.6	[ADPR + DBAA + H]$^+$	689.2307→136.0623	
AMP	6.6	[AMP + DBAA + H]$^+$	477.2222→136.0623	[$^{13}C_{10}$, $^{15}N_5$] AMP 492.2408→146.0642
Acetyl-CoA	8.2	[Acetyl-CoA + H]$^+$	810.1333→303.1379	[1,2-$^{13}C_2$] Acetyl-CoA 812.1400→305.1446
Malonyl-CoA	8.0	[Malonyl-CoA + H]$^+$	854.1229→303.1379	[1,2,3,-$^{13}C_3$] Malonyl-CoA 857.1330→305.1446

The energy charge was calculated using following equation:

$$EC = \frac{[ATP] + 0.5[ADP]}{[ATP] + [ADP] + [AMP]}$$

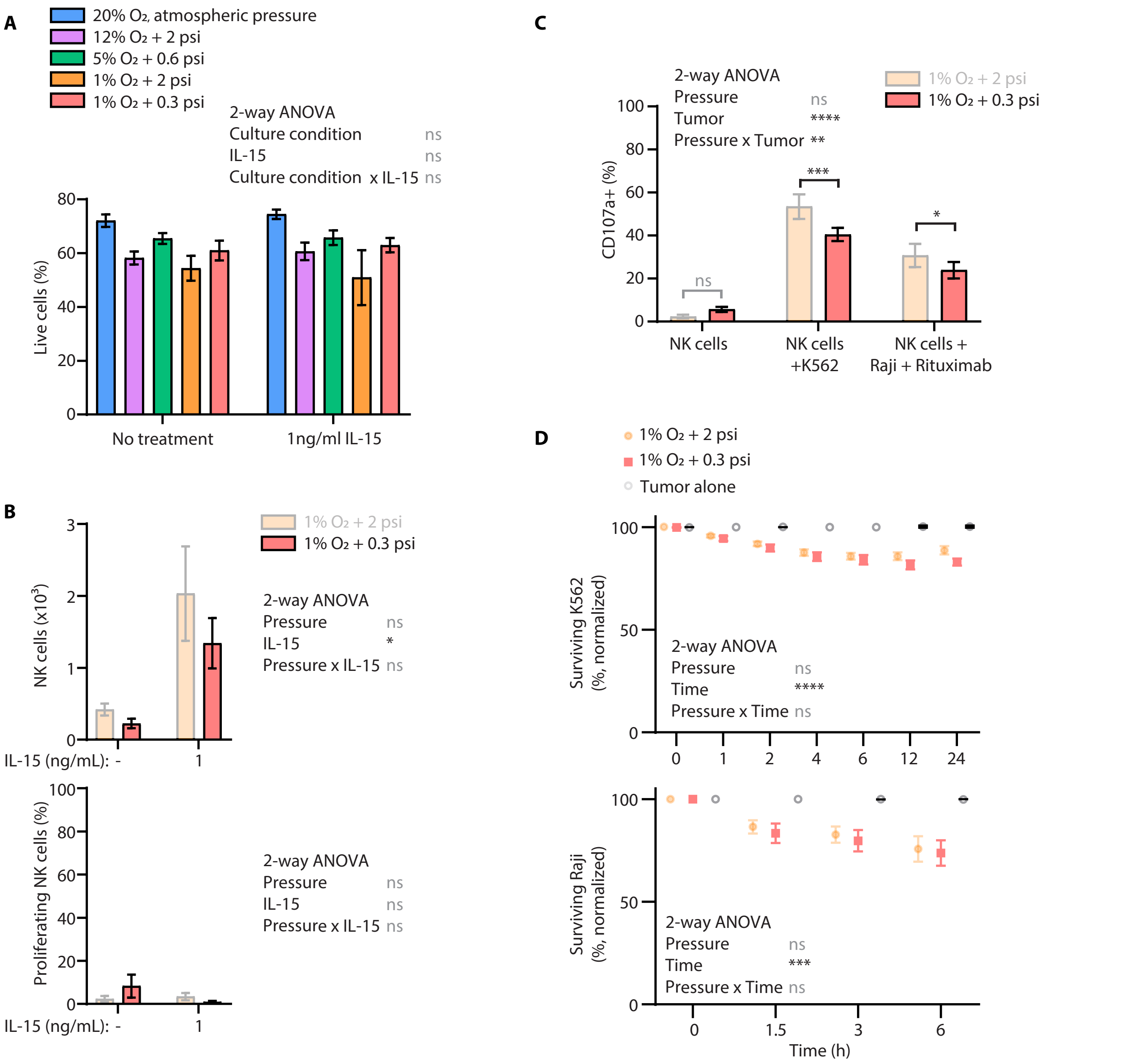

Figure S1. Differential pressures impact NK cell functions less than oxygen content. Hypoxic chambers were pressurized to mimic physiological variables. (**A**) Flow cytometric assessment of NK cell viability (live/dead and annexin V staining; n=4) is shown. A two-way RM ANOVA comparing all conditions was performed, but there was no effect of the culture conditions on viability so no post-hoc tests were performed. In **B-D**, graphs compare culture conditions with the same level of oxygen (severely hypoxic 1% O_2), but different pressures. The high pressure condition is reproduced from Figure 1 and is made opaque to indicate this not new data but is being used as a comparison to the new low pressure oxygen condition. Two-way RM ANOVA were performed comparing the low and high severely hypoxic conditions (B n=7; C n=5; D n=10 for K562, n=8 for Raji) and their interactions with (**B**) IL-15 and its impact on proliferation; (**C**) different tumor target cells and their impact on degranulation (CD107a) and (**D**) the cytotoxic response over time in live cell imaging assays. Where the two-way RM ANOVA indicated a significant impact of pressure ($p \leq 0.05$), post-hoc Sidak's multiple comparison tests were performed, indicated by horizontal bars. In all graphs, vertical bars show the mean and SEM. grey ns (non significant) $p>0.05$ and black $*p \leq 0.05$, $***\ p \leq 0.001$, $****\ p \leq 0.0001$.

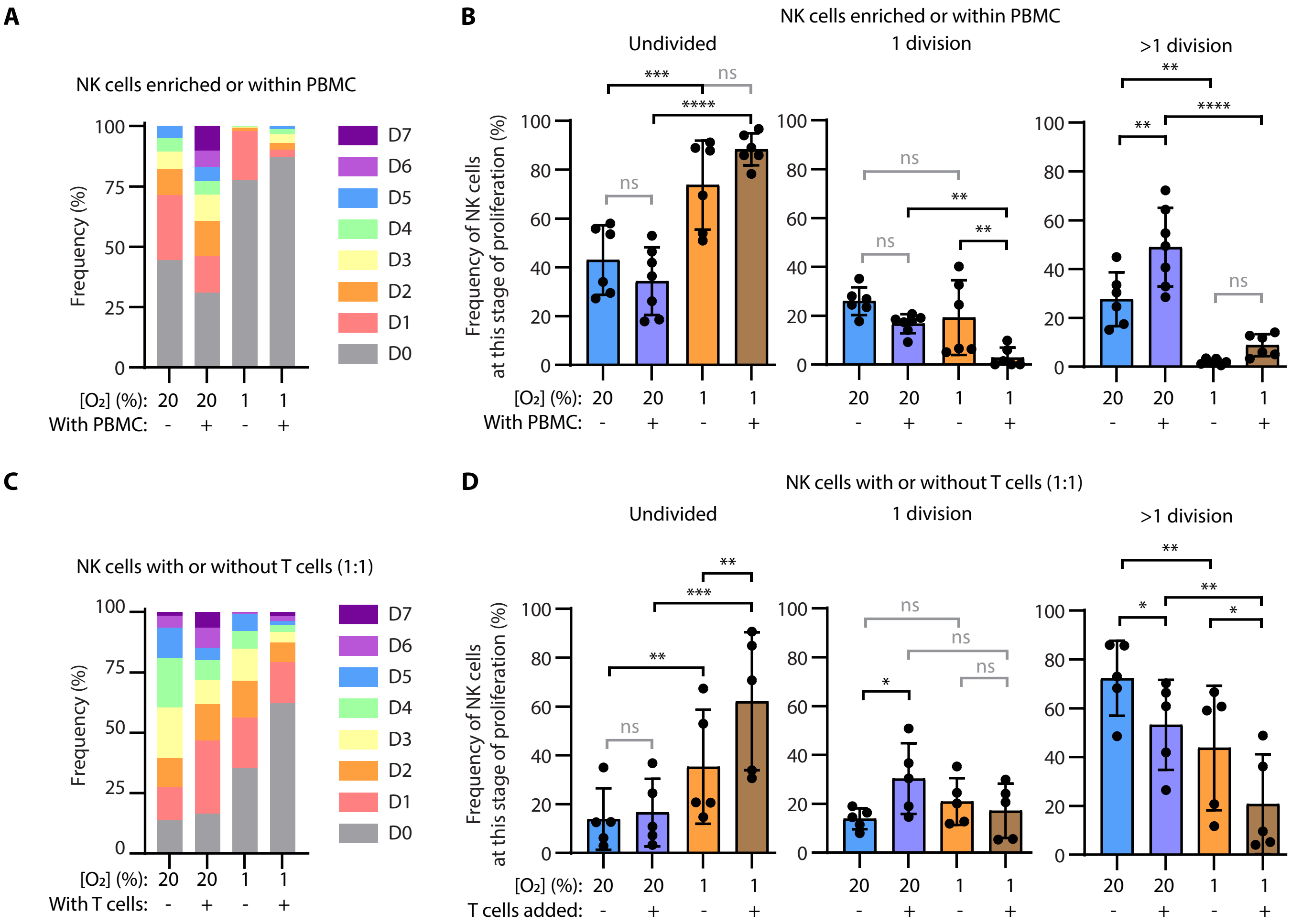

Figure S2. PBMCs enhance NK cell proliferation in normoxia, but T cells limit NK cell proliferation in severe hypoxia. PBMC or enriched NK cells were labeled with CellTrace violet and cultured under 20% O_2 with atmospheric pressure or 1% O_2 + 2 psi. (**A**) The distribution of NK cells amongst daughter generations (0-7) after 7 days was calculated using FlowJo software. Stacked columns show the mean values for 6 (NK cells) or 7 (PBMC) donors (**B**) Individual donor data from (**A**) was plotted separately for the proportion of NK cells that were undivided; had undergone one division; or more than one division. The NK cell and PBMC donors were not matched and the software failed to calculate a peak for one donor in severe hypoxia, so these data were analzyed by mixed effects model. Since O_2 or the presence of PBMC affected the result (* $p \leq 0.05$), these data were analyzed by post-hoc uncorrected Fisher's LSD (indicated by horizontal bars). (**C-D**) NK cells labeled with CellTrace Violet with or without an equal number of T cells were culture for 7 days and presented as above (n=5). In **C-D** NK cell donors were matched within each panel and there were no missing data so they were analyzed by 2-way RM ANOVA with uncorrected Fisher's LSD (indicated by horizontal bars). In all graphs, dots represent donors and vertical bars show mean and standard deviation. ns (not significant) $p > 0.05$, * $p \leq 0.05$, ** $p \leq 0.01$, *** $p \leq 0.001$, **** $p \leq 0.0001$.

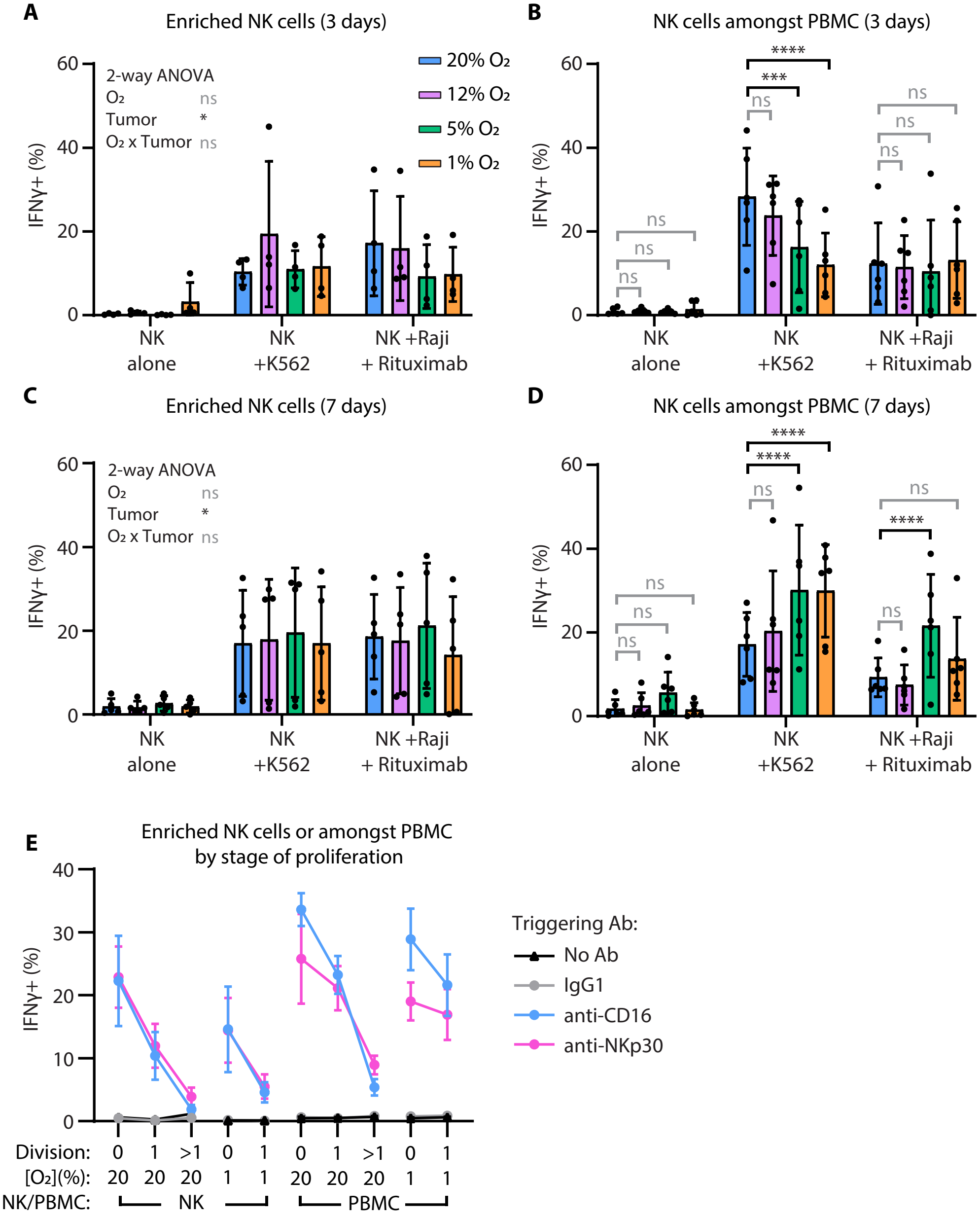

Figure S3. PBMCs alter how NK cells respond to hypoxia, enhacing cytokine production in hypoxia after 7 days. To evaluate cytokine production by NK cells, various NK cell preparations were cultured for 3 days **(A, B)** or 7 days **(C-E)** under specific oxygen and pressure conditions as in Figure 1, then incubated in those same oxygen and pressure conditions for 5 h in the presence of monensin and brefeldin A and various stimulating tumor cells. NK cells were stimulated with (**A-D**) K562 (natural cytotoxicity) or with Raji and rituximab (ADCC) or (**E**) P815 and cross-linking antibodies (Ab) that drive redirected lysis when activating receptors CD16 (blue) or NKp30 (pink) are triggered, but not an isotype control (grey) or no antibody (black). The proportion of the NK cell population that was producing inflammatory cytokines (IFNγ+) was assessed by flow cytometry (live, single, CD56+ CD3-). NK cells were initially enriched from peripheral blood and cultured alone (**A,C, E**) or cultured as peripheral blood mononuclear cells (PBMC; **B, D, E**) for 3-7 days. (**E**) To evaluate the effect of proliferation on cytokine production in hypoxia and normoxia, NK cells were CellTrace Violet labeled prior to 7 day culture and then cytokine production was evaluated for each stage of division (**E**). In **A-D,** vertical bars represent mean and standard deviation. These panels were evaluated by two-way repeated measures ANOVA. No further tests were performed if O_2 or NK cell preparation had no impact on the result ($p \leq 0.05$) and this is reported in the panel, but if these did affect the result post-hoc tests were performed and reported as horizontal bars: Dunnett's multiple comparisons (**A-D**). In **E** vertical bars show mean and SEM and no statistical test was performed. In **A-D**, dots represent different donors. ns (not significant) $p > 0.05$, * $p \leq 0.05$, *** $p \leq 0.001$, **** $p \leq 0.0001$.

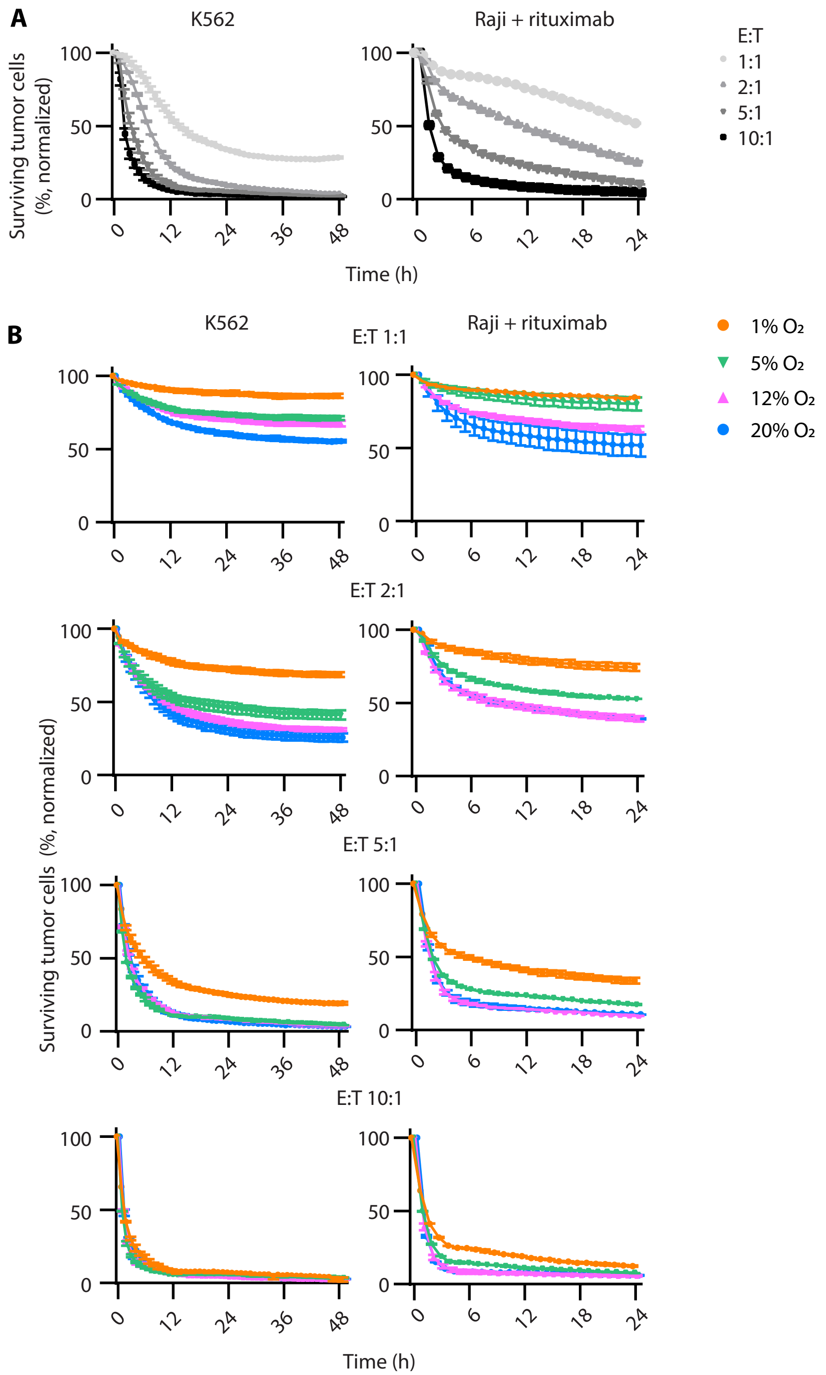

Figure S4. Titration of effector:target (E:T) cell ratios for live cell imaging assays. (**A**) Freshly enriched NK cells on day 0 (n=2) or (**B**) cultured for 7 days at different oxygen concentrations (n=4) were assayed for natural cytotoxicity and antibody-dependent cellular cytotoxicity at the indicated effector: target (E:T ratios). One representative donor is shown. In all graphs, bars represent mean and SEM of measurements in triplicate.

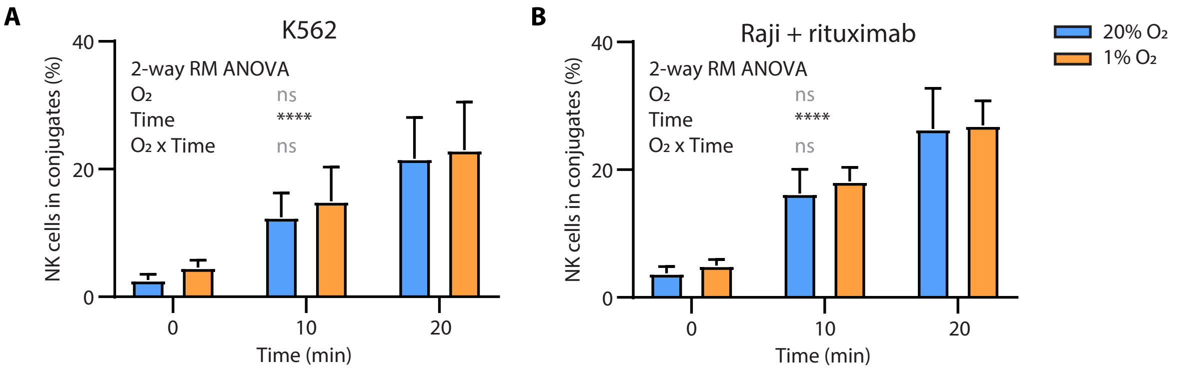

Figure S5. Adhesion remains intact in severe hypoxia The cytotoxic process was examined through flow cytometric analysis of NK cell to tumor cell adhesion. After 7 days of culture, fluorescently labelled NK cells (CellTrace Violet) and target cells (Nuclight Red+) were gently spun together, incubated for the indicated number of minutes and then fixed. The proportion of NK cells forming conjugates with (**A**) K562 or (**B**) Raji in the presence of rituximab was assessed by flow cytometry (n=6; bars show the mean and standard deviation; analyzed by two-way RM ANOVA, but oxygen did not have a significant impact on adhesion). ns (not significant) p>0.05, **** p≤0.0001.

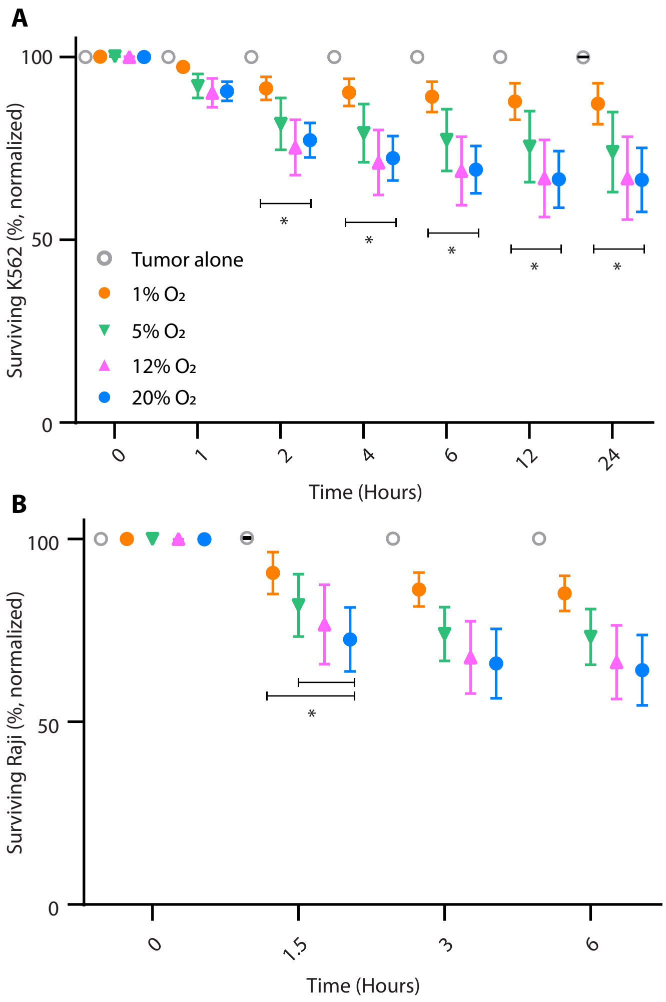

Figure S6. Deficits in killing are apparent after 3 days in severe hypoxia. NK cells were cultured in a range of oxygen concentrations for 3 days before assessment of function. Live cell imaging was used to assess (**A**) natural cytotoxicity against K562 (n=4) and (**B**) antibody-dependent cellular cytotoxicity against Raji cells in the presence of rituximab (n=6). Fluorescent target cells (Nuclight Red+) and an indicator of apoptosis (caspase 3/7 green) were used to assess target cell survival as normalized to target cells alone. In all graphs, vertical bars represent mean and SEM and data were analyzed by two-way RM ANOVA. Since oxygen had a significant impact on the response ($p \leq 0.05$), post-hoc Dunnett's multiple comparisons were used to compare the 20% O_2 condition to all other conditions within a timepoint. Only significant differences ($p \leq 0.05$) are marked by horizontal bars. * $p \leq 0.05$.

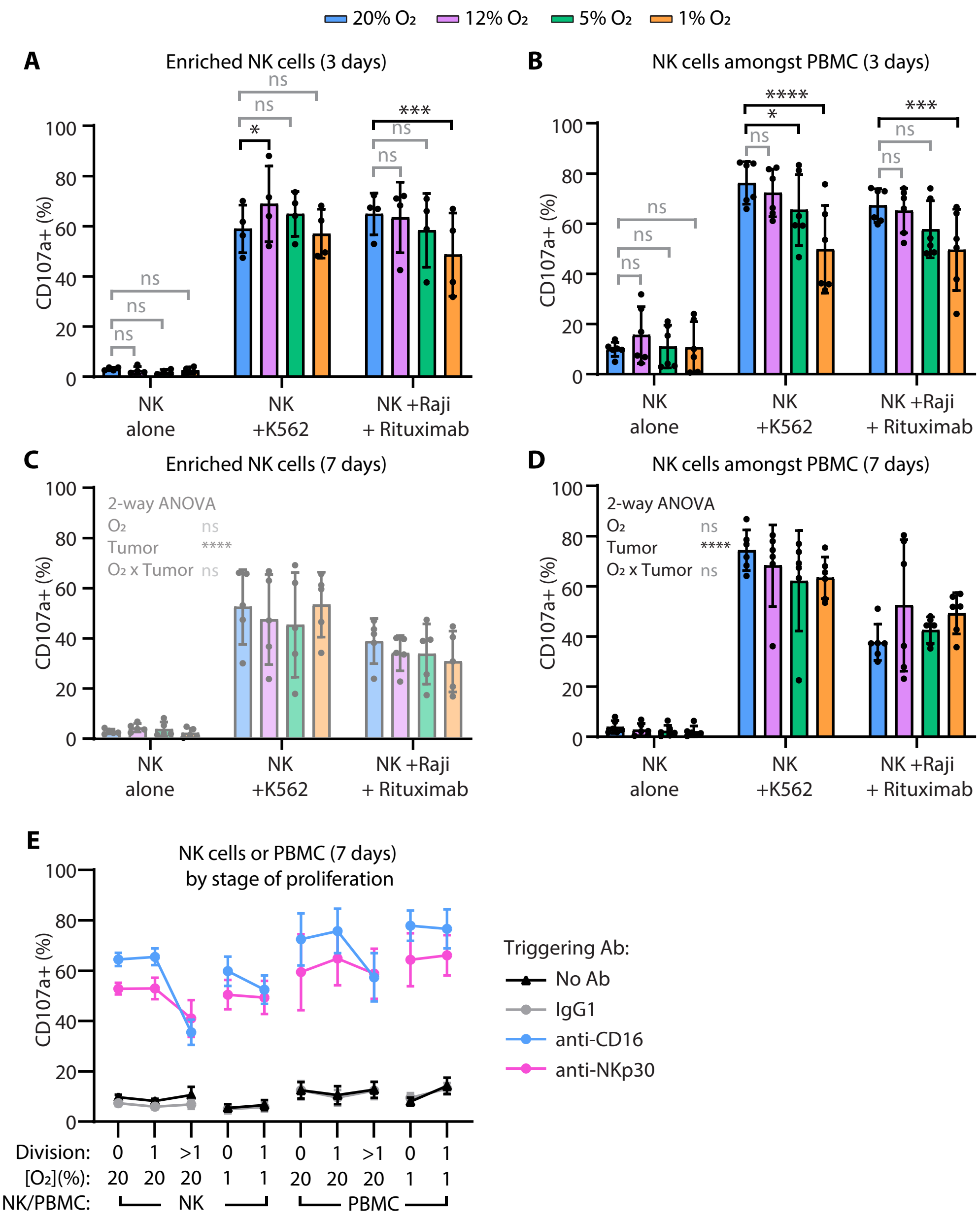

Figure S7. PBMCs have limited impact on how NK cells degranulate in severe hypoxia after 7 days. To evaluate degranulation by NK cells, various NK cell preparations were cultured for 3 days **(A, B)** or 7 days **(C-E)** under specific oxygen and pressure conditions as in Figure 1, then incubated in those same oxygen and pressure conditions for 5 h in the presence of monensin and brefeldin A and various stimulating tumor cells. NK cells were stimulated with (**A-D**) K562 (natural cytotoxicity) or with Raji and riituximab (ADCC) or (**E**) P815 and cross-linking antibodies (Ab) that drive redirected lysis when activating receptors CD16 (blue) or NKp30 (pink) are triggered, but not an isotype control (grey) or no antibody (black). The proportion of the NK cell population that was degranulating (CD107a+) was assessed by flow cytometry (live, single, CD56+ CD3-). NK cells were initially enriched from peripheral blood and cultured alone (**A,C, E**); or cultured as peripheral blood mononuclear cells (PBMC; **B, D, E**) for 3-7 days. (**E**) To evaluate the effect of proliferation on degranulation in hypoxia and normoxia, NK cells were CellTrace Violet labeled prior to 7 day culture and then degranulation was evaluated for each stage of division In **A-D,** vertical bars represent mean and standard deviation and dots represent donors. These panels were evaluated by two-way repeated measures ANOVA. No further tests were performed if O_2 or NK cell preparation had no impact on the result (p≤0.05) and this is reported near the panel, but if these did affect the result post-hoc tests (Dunnett's multiple comparisons) were performed and reported as horizontal bars (**A-D**). In **E** vertical bars show mean and SEM; and no statistical test was performed. **C** appears in Figure 1 and is opaque for reference. ns (not significant) p>0.05, * p≤0.05, *** p≤ 0.001, **** p≤0.0001.

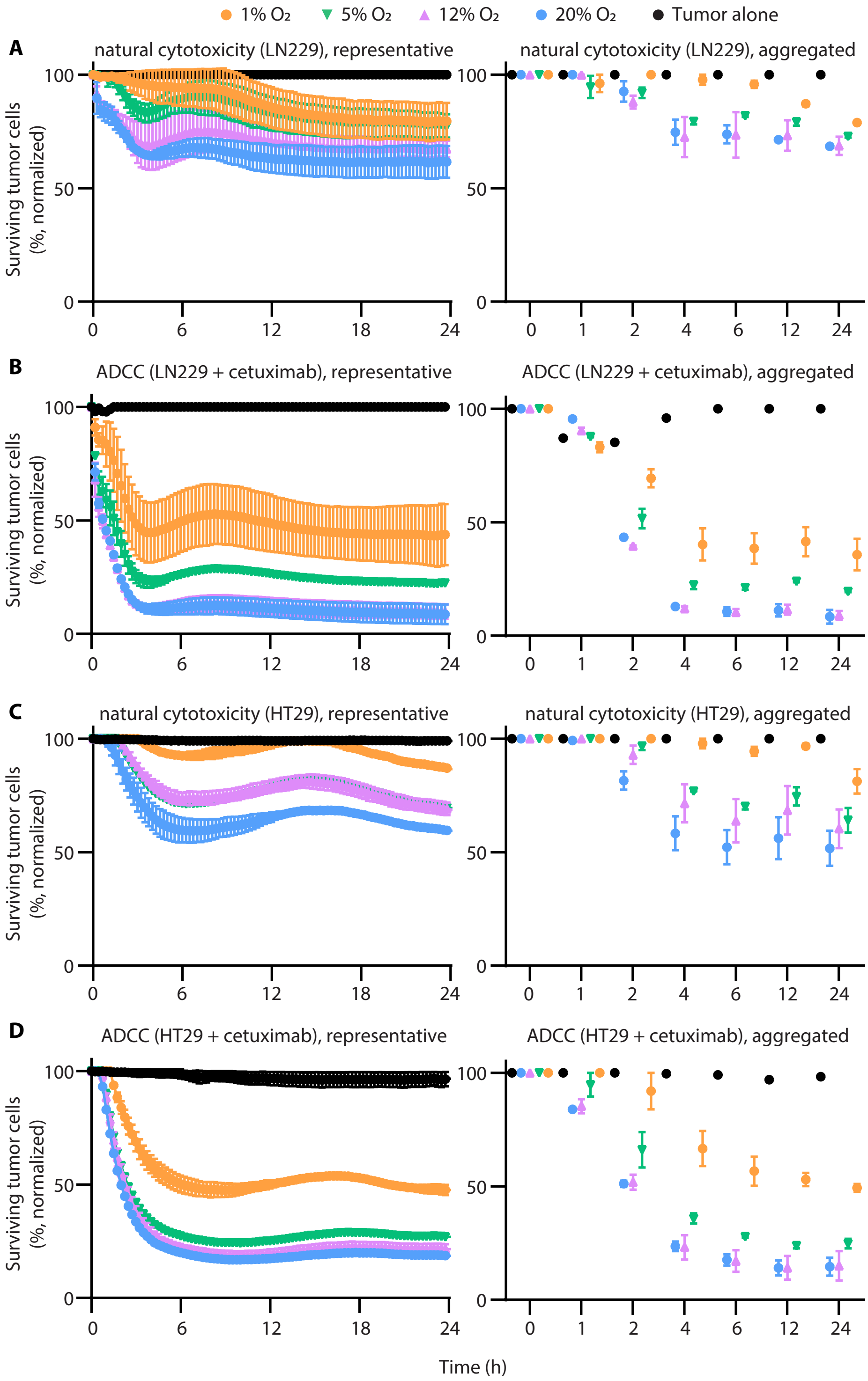

Figure S8. Hypoxia diminishes NK cell cytotoxicity of a wide range of solid tumor lines. NK cells were enriched from blood and cultured under 20% O_2 with atmospheric pressure; 12% O_2 + 2 psi; 5% O_2 + 0.6 psi; or 1% O_2 + 2 psi for 7 days then examined for their ability to kill solid tumor lines by natural cytotoxicity or antibody dependent cellular cytotoxicity (ADCC) with cetuximab. Impedance was used as a measure of solid tumor cell survival, normalized to the growth of tumor cells alone without cetuximab, for brain cancer line, LN229 (**A-B**) and colorectal cancer line, HT29 (**C-D**). In **B** and **D**, all conditions including tumor alone include cetuximab. An effector:target cell ratio of 4:1 and 1 µg/mL cetuximab was selected to have the least impact on tumor growth in the absence of NK cells, but maximal ADCC. In representative graphs (left), conditions were measured in duplicate for one donor and vertical bars show standard deviation of technical replicates. In aggregate graphs (right) data from 2 independent donors is shown and vertical bars show SEM. No statistical test was performed.

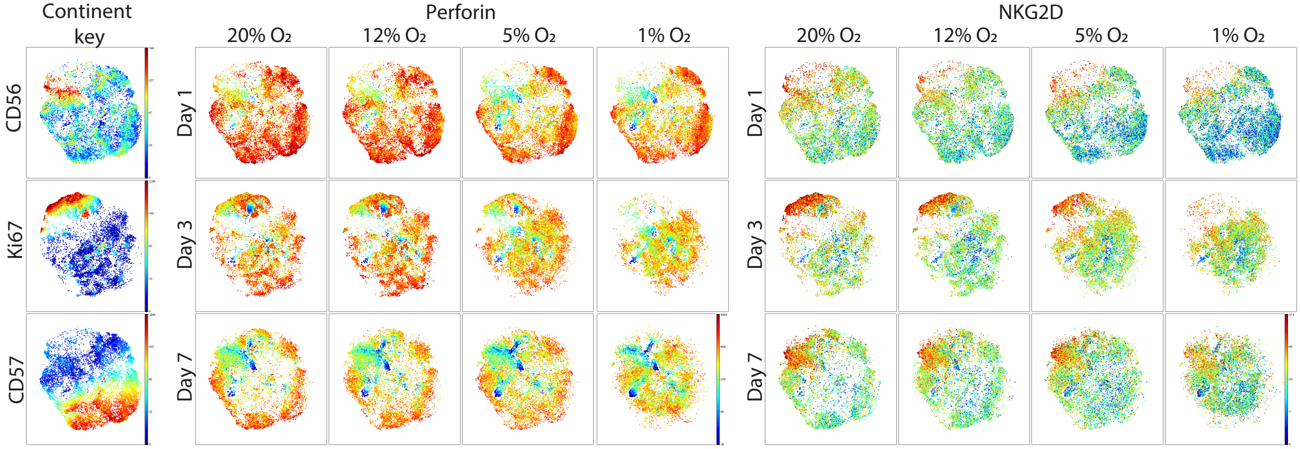

Figure S9. Hypoxia decreases NK cell receptors, cytotoxic proteins and transcription factors, but increases activation markers NK cells were exposed to 20%-1% oxygen for 1-7 days and analyzed by CyTOF. viSNEs generated in Cytobank are organized according to shared features. The key highlights known NK cell subsets: CD56bright cells; Ki67+ proliferating cells and CD57+ mature cells. The remaining plots have the same continent organization, but are colored according to abundance of cytotoxic protein, perforin, or activating receptor, NKG2D. Plots are generated by concatenating data from three donors in FlowJo software.

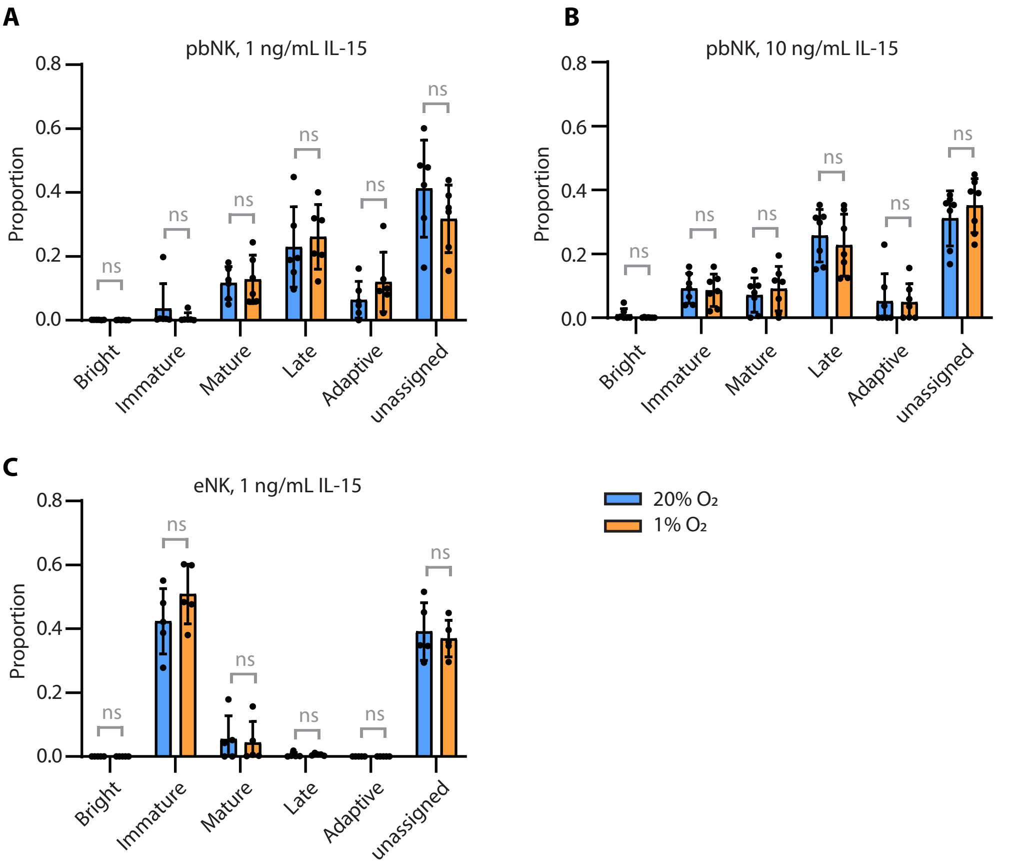

Figure S10. NK cell subsets do not substantially change in frequency upon culture in severe hypoxia. CyTOF analysis was performed on NK cells enriched from blood (**A-B**, pbNK) or thawed NK cells that had previously been expanded with IL-2 and irradiated feeder cells bearing IL-21 and 41BBL (**C**, eNK). These cells were cultured for 7 days in 20% O_2 atmospheric pressure or 1% O_2 + 2 psi with 1 ng/mL IL-15 (**A, C**) or 10 ng/mL IL-15 (**B**) prior to examination. For a much comprehensive explanation of conditions referred to in **B** and **C**, please refer to Figure 7 onwards. The relative size of each NK cell population amongst all identified events ('proportion') is plotted for subsets identified on the basis of maturation makers classically found on the $CD56^{bright}$ subset (CD62L+ CD57- CD16- KIR-) and four $CD56^{dim}$ subsets: Immature (CD62L- CD57- KIR- NKG2A+); Mature (CD62L- CD57- KIR+ NKG2A-); Late stage (CD57+ NKG2C-); and Adaptive (CD57+ NKG2C+). NK cells not assigned to any of these categories (for example, CD62L+ CD16+ cells) were grouped into 'unassigned'. Each dot represents a different donor. Bars show the mean and standard deviation. No significant (ns) differences as measured by differential abundance analysis (astrolabe software), $p>0.05$.

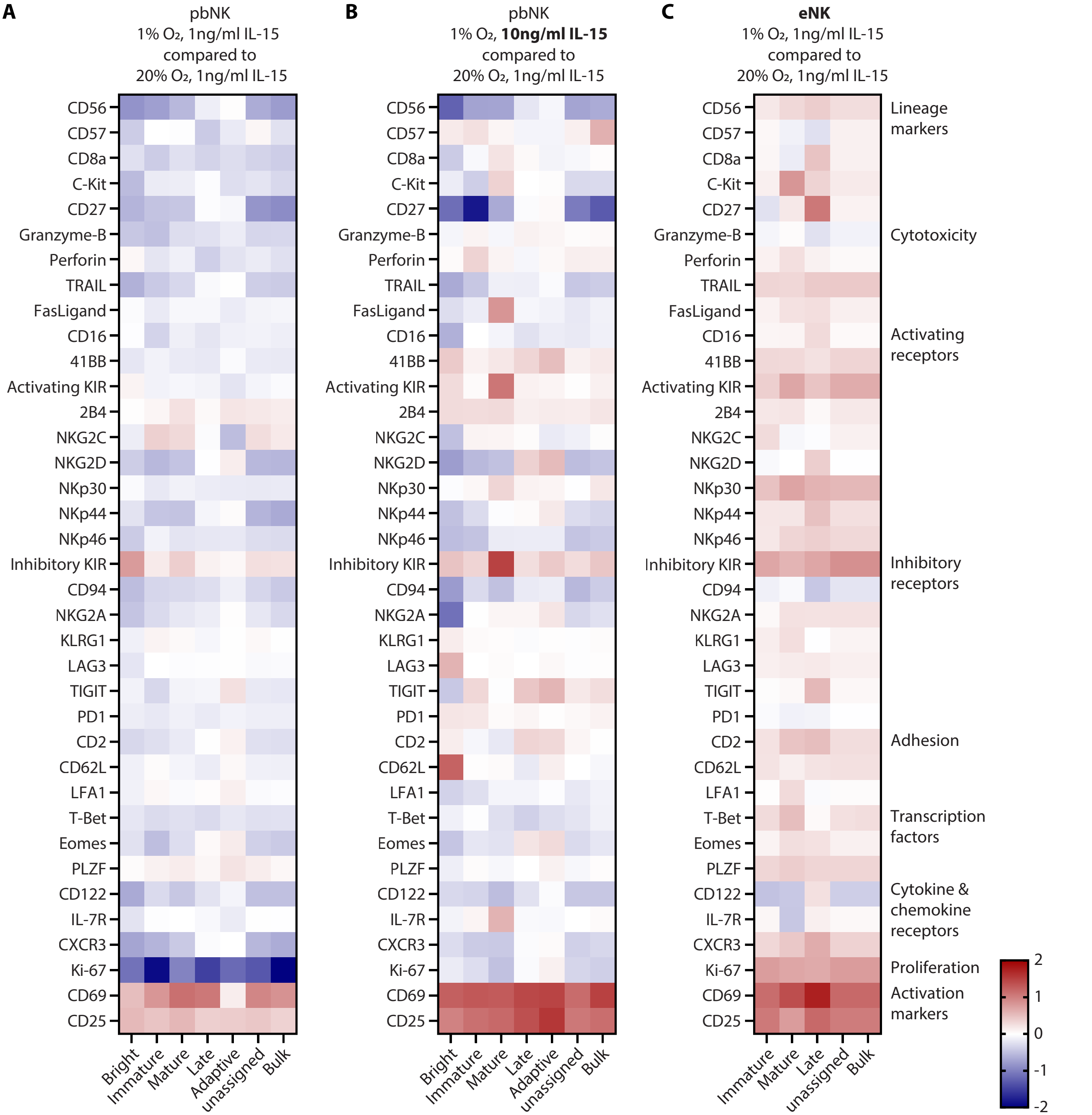

Figure S11. Protein abundance in severe hypoxia follows similar trends for NK cell subsets as the bulk population. CyTOF analysis was performed on NK cells cultured for 7 days in different oxygen conditions. NK cells were enriched from blood (**A-B**, pbNK) or thawed NK cells after an expansion processing involving IL-2 and irradiated feeder cells bearing IL-21 and 41BBL (**C**, eNK). These cells were cultured for 7 days in 20% O_2 atmospheric pressure or 1% O_2 + 2 psi with 1 ng/mL IL-15 (**A, C**) or 10 ng/mL IL-15 (**B**) prior to examination. For a more comprehensive explanation of conditions referred to in **B** and **C**, please refer to Figure 7 onwards. Fold changes in protein abundance are plotted (as in main figures) for all NK cells (CD56+ CD3- : 'Bulk'), or subdivided based upon maturation makers classically found on the $CD56^{bright}$ subset (CD62L+ CD57- CD16- KIR-) and four $CD56^{dim}$ subsets: Immature (CD62L- CD57- KIR- NKG2A+); Mature (CD62L- CD57- KIR+ NKG2A-); Late stage (CD57+ NKG2C-); and Adaptive (CD57+ NKG2C+). NK cells not assigned to any of these categories (for example, CD62L+ CD16+ cells) were grouped into 'unassigned'. Some populations have been underestimated (e.g. $CD56^{brights}$ due to CD62L clipping). It should be noted that fold changes are reported regardless of the size of the subset (see Figure S10 for frequencies).

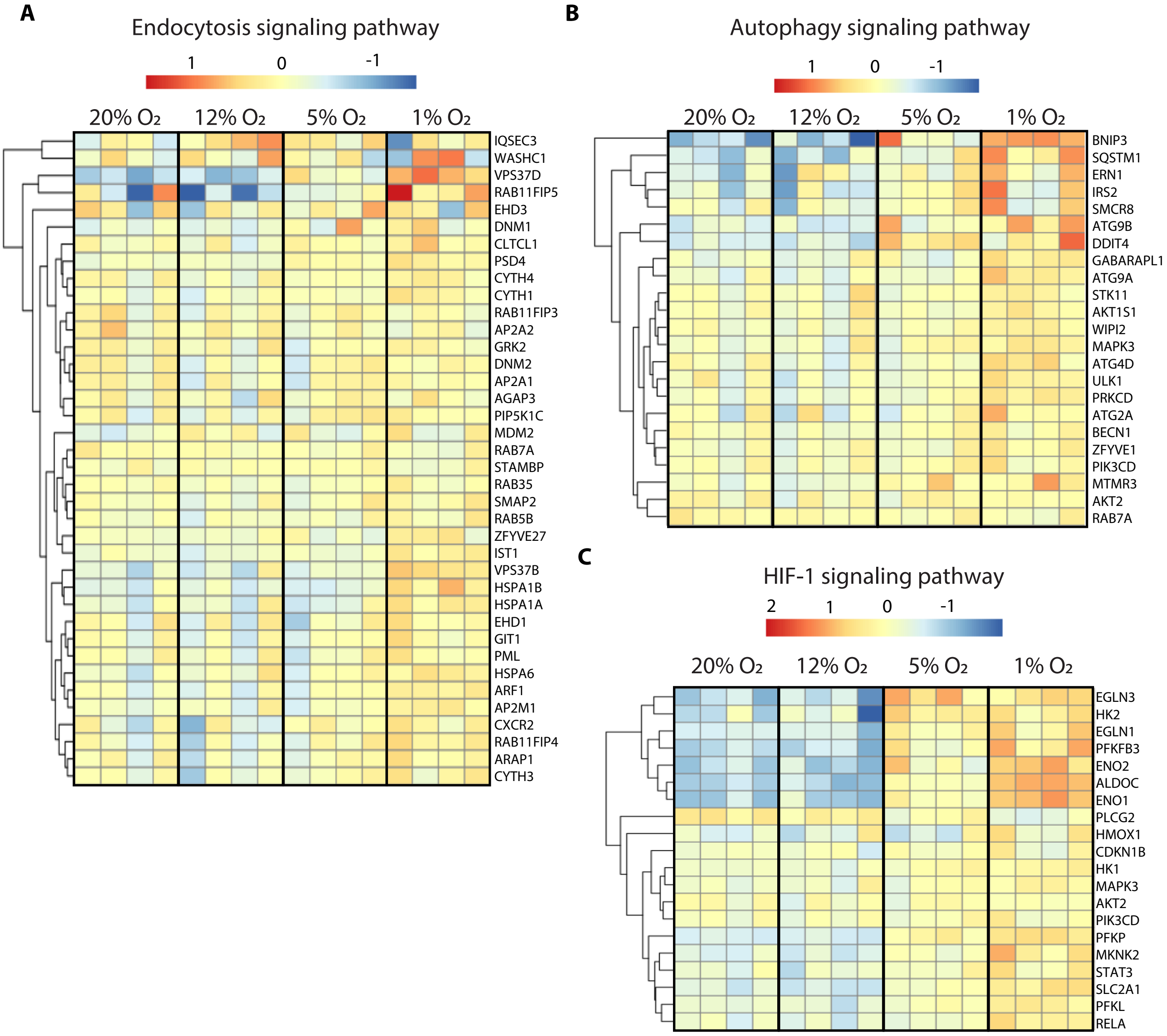

Figure S12. Differentially expressed pathways across oxygen conditions. Heat maps shows the relative expression of genes in the (**A**) Endocytosis, (**B**) Autophagy and (**C**) HIF-1 signaling pathways (KEGG). Each column of squares represents a sample after 7 days of culture at the indicated oxygen concentrations.

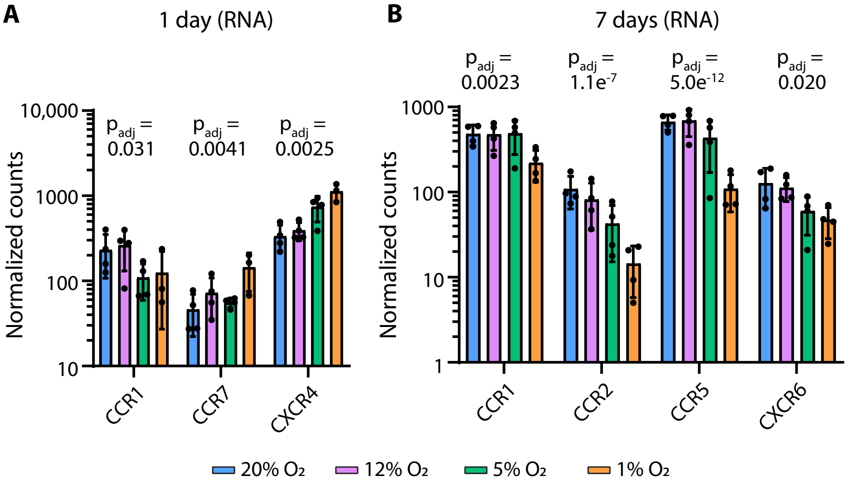

Figure S13. Chemokine receptor expression generally decreases in hypoxia over time, but therapeutic interventions can prevent this defect. (**A-B**) The normalized log counts of chemokine receptor transcripts that were differentially expressed across an oxygen gradient in RNAseq data are plotted for pbNK cells harvested after (**A**) 1 day in culture or (**B**) 7 days in culture with 1 ng/mL IL-15. The adjusted p values from differential expression analysis are noted above each gene. A significant result indicates oxygen impacts normalized counts for that gene. Each dot represents a different donor.

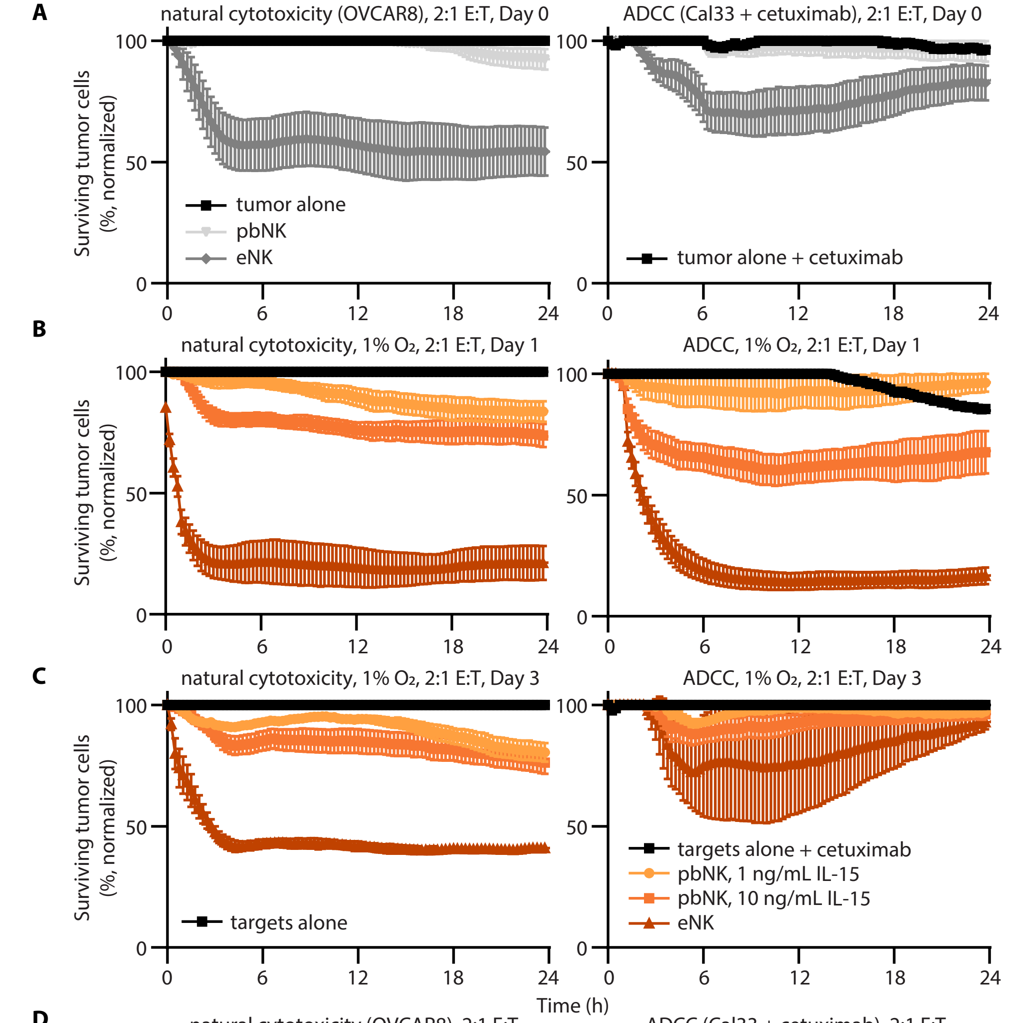

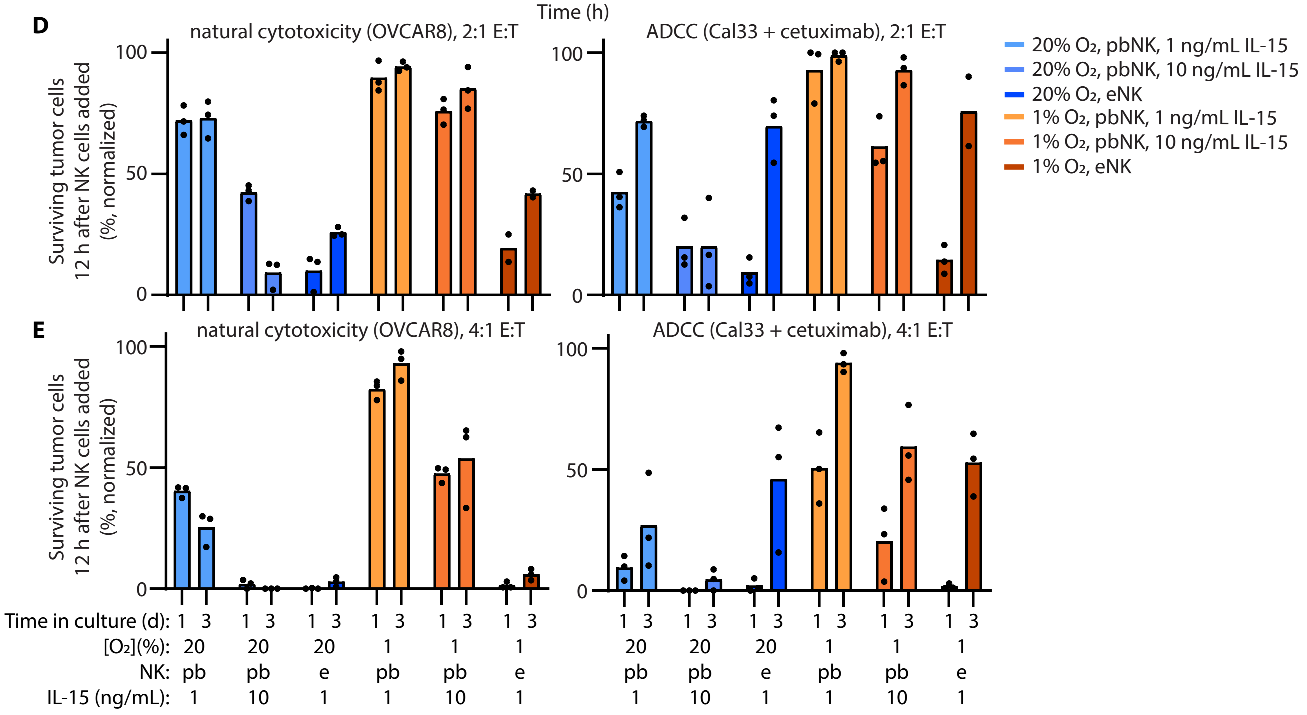

Figure S14. In hypoxia, all therapeutic interventions have peak activity 1 day after treatment and exposure to hypoxia. NK cells were enriched from blood or thawed after an expansion process with feeder cells and then cultured under 20% O_2 with atmospheric pressure or 1% O_2 + 2 psi with 1 or 10 ng/mL IL-15. They were examined for their ability to kill solid tumor lines by natural cytotoxicity (OVCAR8) or antibody dependent cellular cytotoxicity (ADCC; Cal33 with 100 ng/mL cetuximab) either (**A**) freshly isolated without cytokines (Day 0), or (**B**) after 1 day in culture or (**C**) after 3 days in culture. Impedance was used as a measure of solid tumor cell survival, normalized to the growth of tumor cells alone without cetuximab. The tumor alone control in ADCC graphs also contains cetuximab. An effector:target cell ratio of 2:1 is shown in **A-C** and quantified for all oxygen conditions after 12 h of killing in **D**. In **E**, an effector:target ratio of 4:1 is included for reference, since this is the condition used in Figure 2 comparing the effects of oxygen on pbNK cells with 1 ng/mL IL-15. Graphs **A-C** show the mean and SEM (vertical bars) for three independent donors (two independent donors for eNK with Cal33 in hypoxia at 2:1, because one donor did not have enough cells for all conditions). No statistical test was performed.

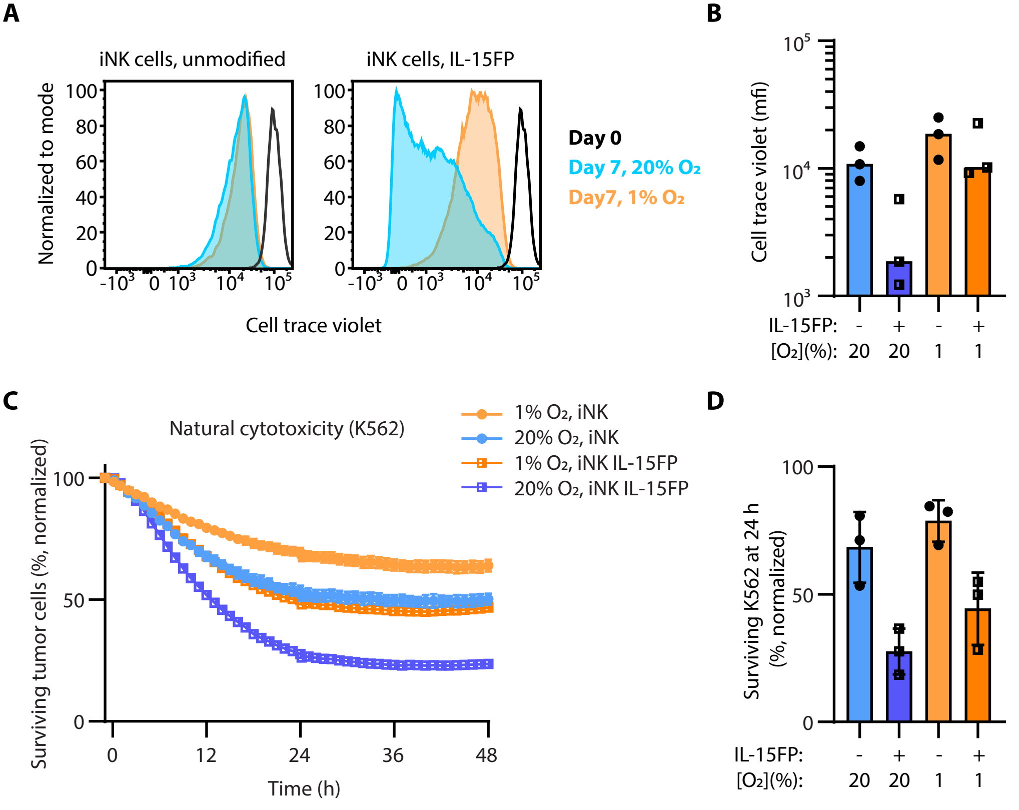

Figure S15. Genetically modified IL-15 intrinsically expressed has similar benefits to exogenous high dose IL-15 on NK cells in severe hypoxia. Induced pluripotent stem cell-derived NK cells (iNK) either unmodified or genetically modified to express a membrane tethered fusion protein of IL-15 and IL-15 receptor α (IL-15FP) were cultured in 20% O_2 or 1% O_2 for 7 days and then evaluated for proliferation and cytotoxicity. 1 ng/mL IL-15 was added exogenously to all cells to support their survival. (**A-B**) CellTrace violet labeling was performed on day 0 (black lines, **A**) and evaluated by flow cytometry on day 7 (blue and yellow lines, **A**). Representative histograms from one experiment are shown in **A** and median fluorescence intensity from 3 experiments is quantified in **B**. Vertical bars show the median and symbols represent individual experiments. (**C-D**) After 7 days in culture these cells were evaluated for their ability to perform natural cytotoxicity by killing K562. Representative survival curves from single experiments are shown (**C**) with data measured in triplicate. Mean survival of the tumor cells at 24 h after addition of NK cells is quantified for three independent experiments (**D**) where vertical bars show the mean and standard deviation. Data are representative only, no statistical tests are performed.

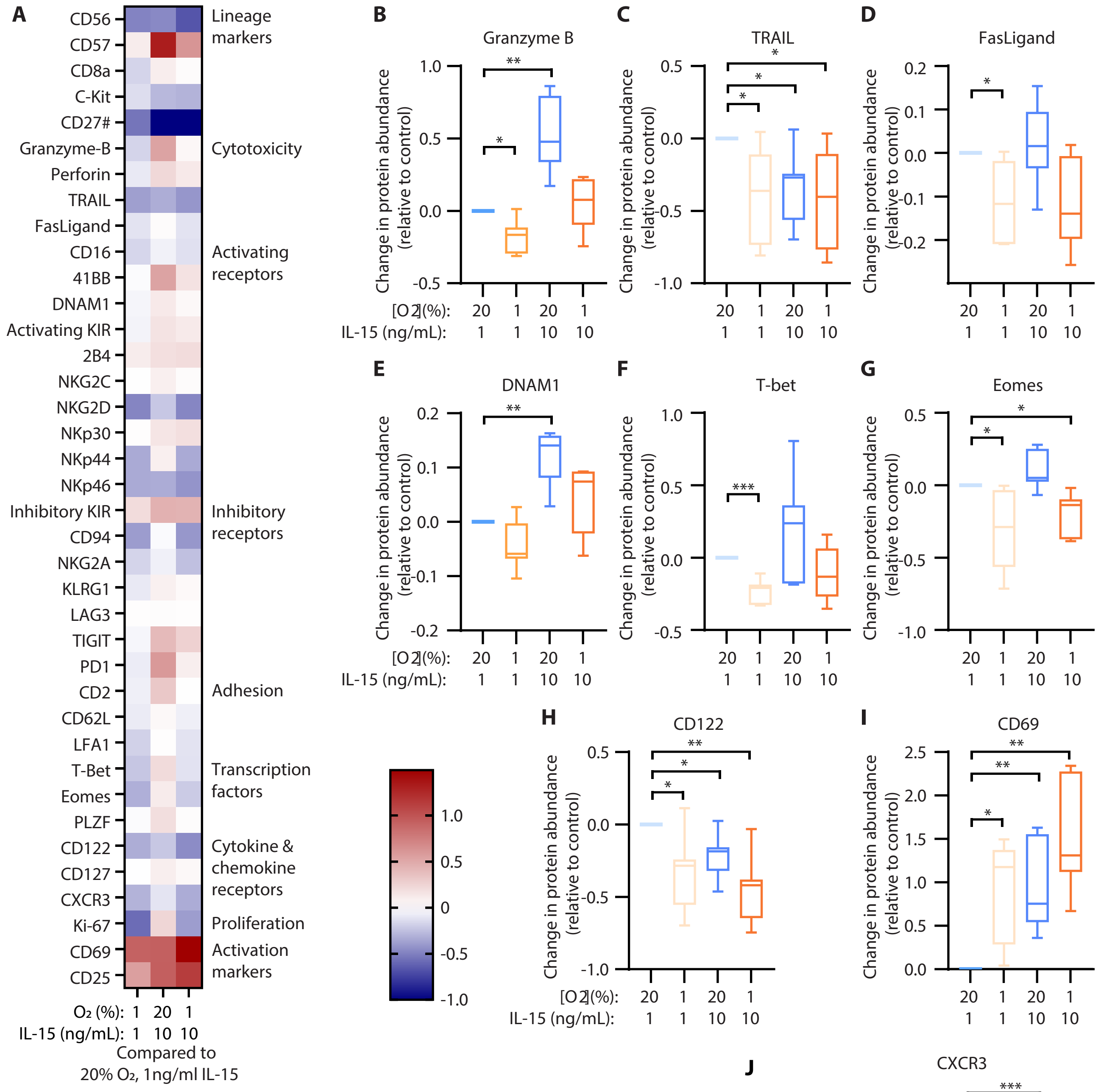

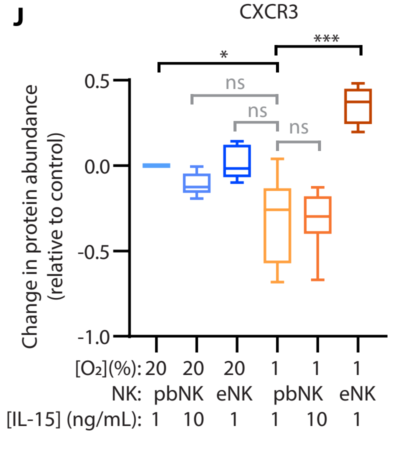

Figure S16. Treatment with IL-15 prevents loss of granzyme B and T-bet in hypoxia. NK cells were exposed to 20% O_2 or 1% O_2 + 2 psi with 1 ng/mL or 10 ng/mL IL-15 for 7 days and then analyzed by CyTOF. The relative abundance of key proteins was quantified by Astrolabe Diagnostics software and normalized to pbNK cells cultured with 1 ng/mL IL-15 in normoxia. In (**A**) each square of the heatmap is the average value for all donors and in (**B-I**) Box (median and IQR) and Whisker plots (min to max) show the spread of the data across donors. Analyzed by RM ANOVA with Dunnett's multiple comparisons compared to 1 ng/mL IL-15 treated normoxic pbNK cells (n=7; matched across all 4 conditions), indicated by horizontal bars if p>0.05. (**J**) The abundance of CXCR3 is presented as above, but with the inclusion of eNK cells. Kruskal-Wallis test with Dunn's multiple comparisons compares pbNK cultured in 1% O_2 with 1 ng/mL IL-15 to all other conditions. All tests performed are marked by a horizontal bar, ns p>0.05, *p≤0.05, ** p≤0.01, *** p≤0.001. In **A**, # indicates two values for CD27 are below the indicated scale. NK cells treated with 10 ng/ml IL-15 and 20% O_2 (-1.38) or 1% O_2 (-1.32) had less CD27 than the control. pbNK data in **C, D, F, G, H, I** is opaque because it already appears in Figures 8 and 10, but is included here for reference.

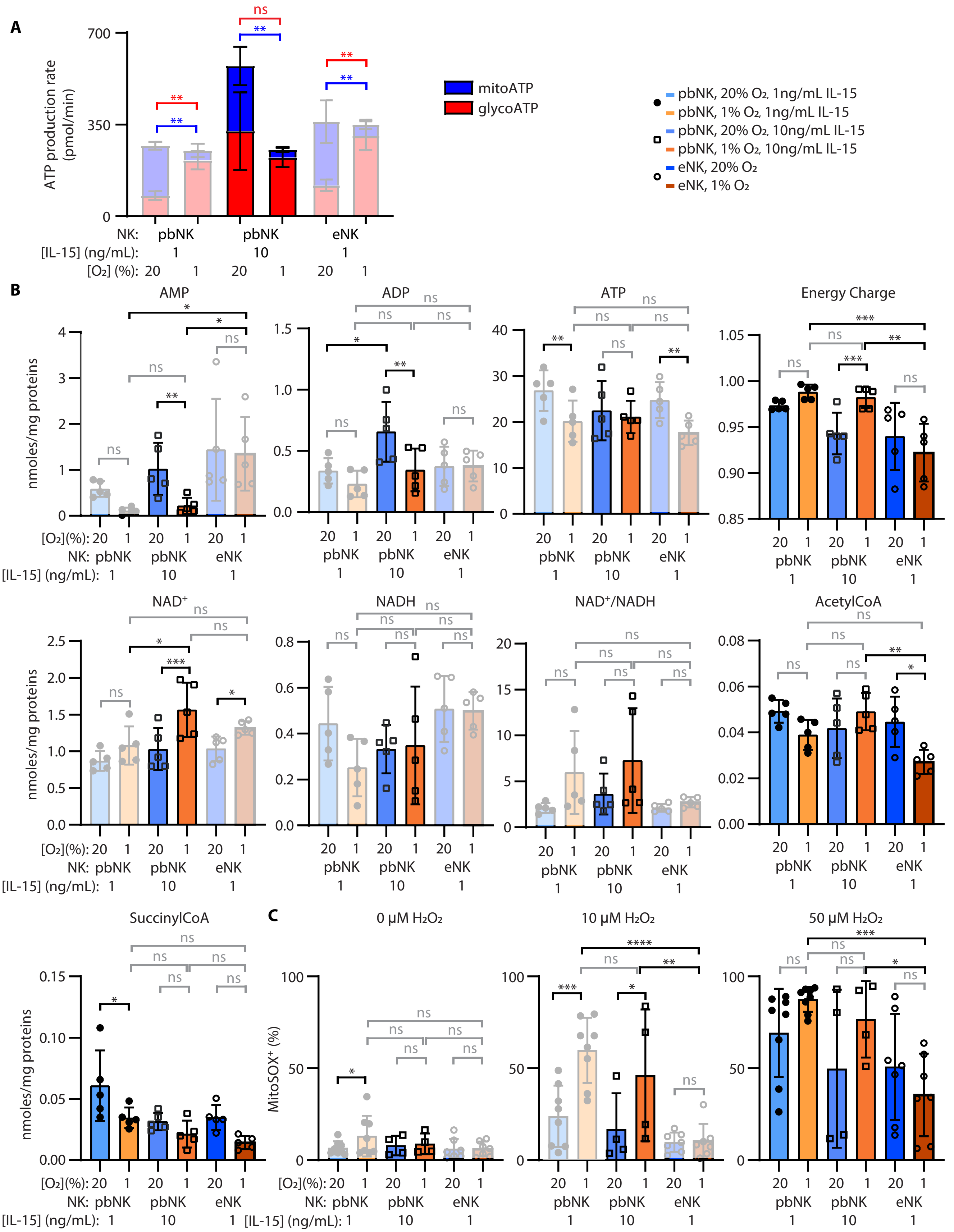

Figure S17. The redox balance under hypoxia is similar in pbNK cells treated with low or high dose IL-15. Where data for pbNK cultured in 1 ng/mL IL-15 and eNK cells is copied from main figures, it is included for reference only (opaque). (**A**) The rate of ATP generation via glycolysis (glycoATP) or oxidative phosphorylation (mitoATP) was measured for pbNK cells cultured in 10 ng/mL IL-15 (n=5) in 20% O_2 or 1% O_2. The rates of ATP generation by glycolysis or oxidative phosphorylation were compared for different oxygen conditions. (**B**) The energy status of cultured NK cells (as previously) was determined by analytical chemistry (n=5); bars show the mean and standard deviation. (**C**) ROS (mitoSOX+) were detected by flow cytometry within live NK cells (TOPRO-3-, singlets, CD56+, CD3-) when exposed to increasing doses of hydrogen peroxide (H_2O_2; pbNK 10 ng/mL n=4; bars show the mean and standard deviation). All graphs were analyzed by two-way ANOVA with Sidak's multiple comparison test * p≤0.05, ** p≤0.01, *** p≤0.001,**** p≤ 0.0001.

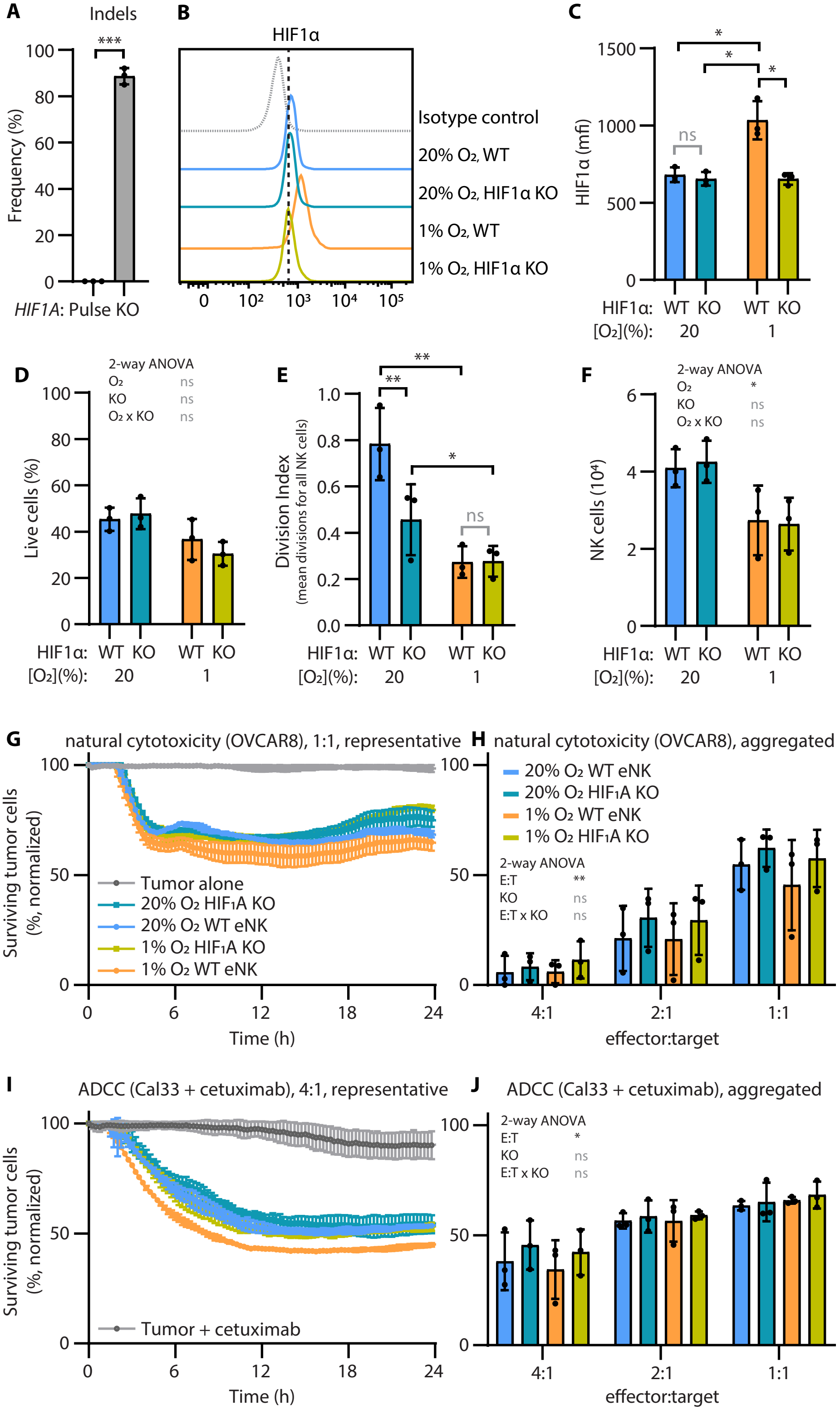

Figure S18. Knocking out HIF1alpha in eNK cells has minimal impact on cytotoxicity in severe hypoxia. NK cells were expanded on irradiated feeder cells for 7 days before being electroporated with or without sgRNA for HIF1α CRISPR KO. After 3 days of recovery, the NK cells were analysed for the frequency of indels by inference of CRISPR edits (ICE) analysis (**A**, analyzed by a paired t-test). These cells were then expanded on irradiated feeder cells for a further 7 days before being frozen. After thaw, eNK cells were labeled with CellTrace Violet and cultured in 20% or 1% O_2 for 7 days. (**B-F**) At the end of 7 days NK cells (CD56+ CD3-) were evaluated by flow cytometry for the abundance of HIF1α (**B-C**), shown as representative histograms for one donor (**B**), where the dotted line highlights the peak staining of the KO under hypoxic conditions, and quantification of median fluorescence intensity (mfi) for three donors (**C**). Flow cytometry was also used to evalute (**D**) viability from annexin V and live/dead staining, (**E**) the division index, a measure of proliferation, calculated using CellTrace dilution in FlowJo software and (**F**) the relative number of NK cells per well at the end of culture. These cells were also evaluated for natural cytotoxicity (**G-H**) and ADCC (**I-J**) by impedance, as previously described. Representative impedance values, normalized to the tumor alone are shown for a single donor and effector:target ratio (**G,I**) alongside quantification of the normalized impedance values for 3 donors, 12 h after NK cells were added to tumor cells (**H,J**). **C-F, H and J** were analyzed by 2-way RM ANOVA. If the effect of the KO was p>0.05 (not significant) this is indicated and no further tests were performed. If the effect of the KO was p≤0.05, horizontal bars show the result of Tukey's multiple comparison tests. In all graphs, dots represent donors and vertical bars show the mean and standard deviation. ns (not significant) p>0.05, * p≤0.05, ** p≤0.01.

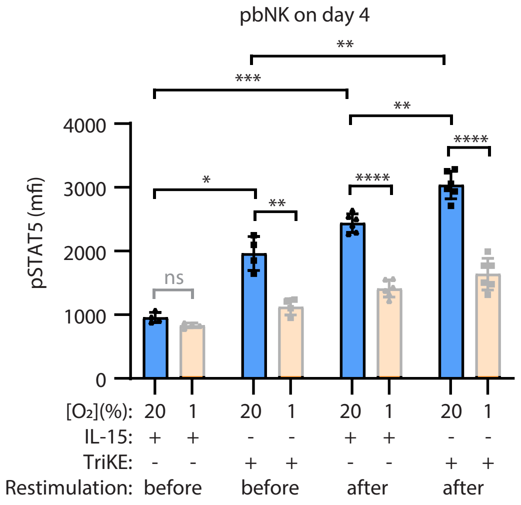

Figure S19. STAT5 signaling is dampened in severe hypoxia compared to normoxia pbNK cells were incubated with 3 nM mesothelin-targeting TriKE or an equifunctional dose of IL-15. Drugs were added on day 0 and they were restimulated on day 4. The median fluorescence intensity of the pSTAT5 signal on day 4, before (n=4) or after (n=6) restimulation with TriKE or IL-15 is quantified for independent donors. Analyzed by mixed effects analysis. Horiztonal bars indicate post-hoc tests with Sidak's multiple comparisons; vertical bars shown the mean and standard deviation. ns (not significant) $p>0.05$, * $p\leq0.05$, ** $p\leq0.01$, *** $p\leq0.001$, **** $p\leq0.0001$. Where hypoxic data already appears in Figure 10, but are included for reference, these data are opaque.

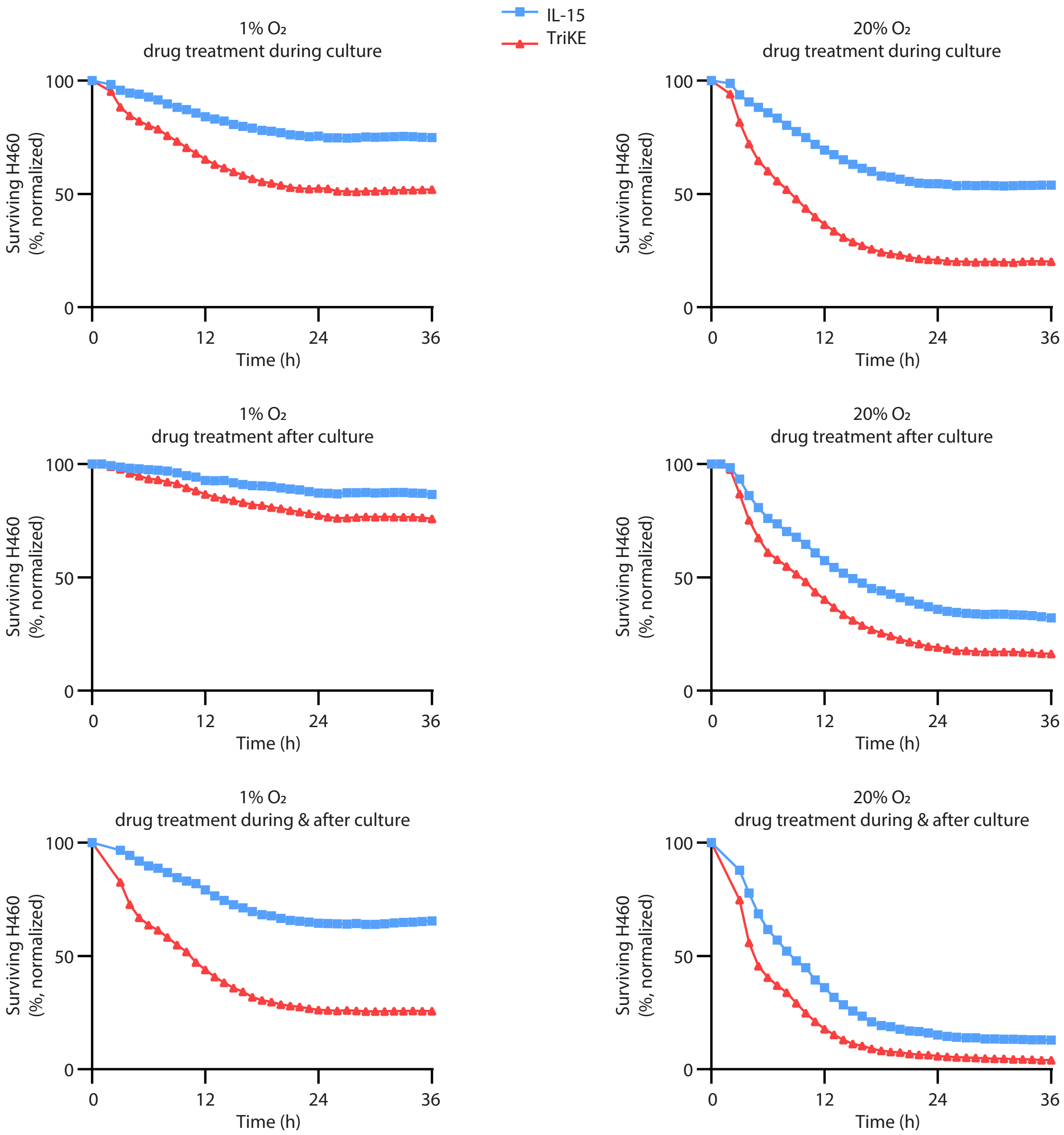

Figure S20. TriKE improves NK cell functionality in severe hypoxia when administered during exposure to hypoxia or after. Representative graphs show NK cell killing of H460 cells after 7 day culture in 1% or 20% O_2 with drugs administered during culture (n=4), following culture during the cytotoxicity assay (n=2) or during and following culture (n=4). Symbols represent the mean, measured in triplicate.

Supplementary Table 1: reagent list for time of flight mass cytometry										
Panel 1 - Cytobank (41 channels) - Figure S9 only						Panel 2 - Cytobank (46 channels) - all other figures				
Usage	Metal tag	Cat.	Company	Cocktail		Usage	Metal tag	Cat.	Company	Cocktail
Cisplatin	Pt195Di	201064	Fluidigm			Cisplatin	Pt195Di	201064	Fluidigm	
Cell-ID™ Intercalator-Ir	Ir191Di, Ir19	201192A	Fluidigm			Cell-ID™ Intercalator-Ir	Ir191Di, Ir193Di	201192A	Fluidigm	
Cell-ID 20-Plex Pd Barcoding Kit	Pd102Di, Pd104Di, Pd105Di, Pd106Di, Pd108Di, Pd110Di	201060	Fluidigm			Cell-ID 20-Plex Pd Barcoding Kit	Pd102Di, Pd104Di, Pd105Di, Pd106Di, Pd108Di, Pd110Di	201060	Fluidigm	
CD11B	Bi209Di	3209003B	Fluidigm	surface		CD11B	Bi209Di	3209003B	Fluidigm	surface
CD127	Nd143Di	3143012B	Fluidigm	surface		CD127	Nd143Di	3143012B	Fluidigm	surface
CD137	Gd158Di	3158013B	Fluidigm	surface		CD137	Gd158Di	3158013B	Fluidigm	surface
CD14	Eu151Di	3151009B	Fluidigm	surface		CD14	Eu151Di	3151009B	Fluidigm	surface
CD16	Nd148Di	3148004B	Fliudigm	surface		CD16	Nd148Di	3148004B	Fliudigm	surface
CD19	Nd142Di	3142001B	Fluidigm	surface		CD19	Nd142Di	3142001B	Fluidigm	surface
CD223	Ho165Di	3165037B	Fluidigm	surface		CD223	Ho165Di	3165037B	Fluidigm	surface
CD226	Yb171Di	3171013B	Fluidigm	surface		CD226	Yb171Di	3171013B	Fluidigm	surface
CD25	Sm149Di	3149010B	Fluidigm	surface		CD25	Sm149Di	3149010B	Fluidigm	surface
CD27	Gd155Di	3155001B	Fluidigm	surface		CD27	Gd155Di	3155001B	Fluidigm	surface
CD274/PDL1	Gd156Di	3156026B	Fluidigm	surface		CD274/PDL1	Gd156Di	3156026B	Fluidigm	surface
CD3	Er170Di	3170001B	Fluidigm	surface		CD3	Er170Di	3170001B	Fluidigm	surface
CD314	Er166Di	3166016B	Fluidigm	surface		CD314	Er166Di	3166016B	Fluidigm	surface
CD33	Dy163Di	3163023B	Fluidigm	surface		CD33	Dy163Di	3163023B	Fluidigm	surface
CD335	Dy162Di	3162021B	Fluidigm	surface		CD335	Dy162Di	3162021B	Fluidigm	surface
CD337	Tb159Di	3159017B	Fluidigm	surface		CD337	Tb159Di	3159017B	Fluidigm	surface
CD4	Nd145Di	3145001B	Fluidigm	surface		CD4	Nd145Di	3145001B	Fluidigm	surface
CD45	Y89Di	3089003B	Fluidigm	surface		CD45	Y89Di	3089003B	Fluidigm	surface
CD56	Yb176Di	3163007B	Fluidigm	surface		CD56	Yb176Di	3163007B	Fluidigm	surface
CD57	Yb172Di	3172009B	Fluidigm	surface		CD57	Yb172Di	3172009B	Fluidigm	surface
CD62L	Eu153Di	3153004B	Fluidigm	surface		CD62L	Eu153Di	3153004B	Fluidigm	surface
CD69	Nd144Di	3144018B	Fluidigm	surface		CD69	Nd144Di	3144018B	Fluidigm	surface
CD8a	Nd146Di	3146001B	Fluidigm	surface		CD8a	Nd146Di	3146001B	Fluidigm	surface
CD95	Sm152Di	3152017B	Fluidigm	surface		CD95	Sm152Di	3152017B	Fluidigm	surface
FcεRL		334602	Biolegend	surface		FcεRL		334602	Biolegend	surface
(tagging kit)	Nd150Di	201150A	Fluidigm			(tagging kit)	Nd150Di	201150A	Fluidigm	
Granzyme B	Yb173Di	3173006B	Fluidigm	intracellular		Granzyme B	Yb173Di	3173006B	Fluidigm	intracellular
HLA-ABC		311402	Biolegend	surface		HLA-ABC		311402	Biolegend	surface
(tagging kit)	Pr141Di	201141A	Fluidigm			(tagging kit)	Pr141Di	201141A	Fluidigm	
Ki-67	Er168Di	3168007B	Fluidigm	intracellular		Ki-67	Er168Di	3168007B	Fluidigm	intracellular
NKG2A	Tm169Di	3169013B	Fluidigm	surface		NKG2A	Tm169Di	3169013B	Fluidigm	surface
PD-1	Yb174Di	3174020B	Fluidigm	surface		PD-1	Yb174Di	3174020B	Fluidigm	surface
Perforin	Lu175Di	3175004B	Fluidigm	intracellular		Perforin	Lu175Di	3175004B	Fluidigm	intracellular
TIGIT	Sm154Di	3209002B	Fluidigm	surface		TIGIT	Sm154Di	3209002B	Fluidigm	surface
						TRAIL	Dy164Di	201164A	Fluidigm	surface
						NKG2C	Gd160Di	201160A	Fluidigm	surface
						NKp44	Er167Di	201167A	Fluidigm	surface
						FoxP3		320102	Biolegend	intracellular
						(tagging kit)	Dy161Di	201161A	Fluidigm	
						FasLigand	Sm147Di	201147A	Fluidigm	surface

REFERENCES AND NOTES

1. S. Liu, V. Galat4, Y. Galat, Y. K. A. Lee, D. Wainwright, J. Wu, NK cell-based cancer immunotherapy: From basic biology to clinical development. *J. Hematol. Oncol.* **14**, 7 (2021).

2. J. S. Miller, L. L. Lanier, Natural killer cells in cancer immunotherapy. *Annu. Rev. Cancer Biol.* **3**, 77–103 (2019).

3. L. Barkholt, E. Alici, R. Conrad, T. Sutlu, M. Gilljam, B. Stellan, B. Christensson, H. Guven, N. K. Björkström, G. Söderdahl, K. Cederlund, E. Kimby, J. Aschan, O. Ringdén, H.-G. Ljunggren, M Sirac Dilber, Safety analysis of ex vivo-expanded NK and NK-like T cells administered to cancer patients: A phase I clinical study. *Immunotherapy* **1**, 753–764 (2009).

4. E. Liu, D. Marin, P. Banerjee, H. A. MacApinlac, P. Thompson, R. Basar, L. N. Kerbauy, B. Overman, P. Thall, M. Kaplan, V. Nandivada, I. Kaur, A. N. Cortes, K. Cao, M. Daher, C. Hosing, E. N. Cohen, P. Kebriaei, R. Mehta, S. Neelapu, Y. Nieto, M. Wang, W. Wierda, M. Keating, R. Champlin, E. J. Shpall, K. Rezvani, Use of CAR-transduced natural killer cells in CD19-positive lymphoid tumors. *N. Engl. J. Med.* **382**, 545–553 (2020).

5. J. A. Olson, D. B. Leveson-Gower, S. Gill, J. Baker, A. Beilhack, R. S. Negrin, NK cells mediate reduction of GVHD by inhibiting activated, alloreactive T cells while retaining GVT effects. *Blood* **115**, 4293–4301 (2010).

6. S. K. Phung, J. S. Miller, M. Felices, Bi-specific and tri-specific NK cell engagers: The new avenue of targeted NK cell immunotherapy. *Mol. Diagn. Ther.* **25**, 577–592 (2021).

7. T. O. Robinson, K. S. Schluns, The potential and promise of IL-15 in immuno-oncogenic therapies. *Immunol. Lett.* **190**, 159–168 (2017).

8. W. Wang, NK cell-mediated antibody-dependent cellular cytotoxicity in cancer immunotherapy. *Front. Immunol.* **6**, 368 (2015).

9. M. M. Berrien-Elliott, J. A. Wagner, T. A. Fehniger, Human cytokine-induced memory-like natural killer cells. *J. Innate Immun.* **7**, 563–571 (2015).

10. A. Biederstädt, K. Rezvani, Engineering the next generation of CAR-NK immunotherapies. *Int. J. Hematol.* **114**, 554–571 (2021).

11. J. S. Miller, Y. Soignier, A. Panoskaltsis-Mortari, S. A. McNearney, G. H. Yun, S. K. Fautsch, D. McKenna, C. Le, T. E. Defor, L. J. Burns, P. J. Orchard, B. R. Blazar, J. E. Wagner, A. Slungaard, D. J. Weisdorf, I. J. Okazaki, P. B. McGlave, Successful adoptive transfer and in vivo expansion of human haploidentical NK cells in patients with cancer. *Blood* **105**, 3051–3057 (2005).

12. L. Tong, C. Jiménez-Cortegana, A. H. M. Tay, S. Wickström, L. Galluzzi, A. Lundqvist, NK cells and solid tumors: Therapeutic potential and persisting obstacles. *Mol. Cancer* **21**, 206 (2022).

13. P. R. Kennedy, M. Felices, J. S. Miller, Challenges to the broad application of allogeneic natural killer cell immunotherapy of cancer. *Stem Cell Res. Ther.* **13**, 165 (2022).

14. A. Sebestyén, L. Kopper, T. Dankó, J. Tímár, Hypoxia signaling in cancer: From basics to clinical practice. *Pathol. Oncol. Res.* **27**, 1609802 (2021).

15. V. Bhandari, C. Hoey, L. Y. Liu, E. Lalonde, J. Ray, J. Livingstone, R. Lesurf, Y.-J. Shiah, T. Vujcic, X. Huang, S. M. G. Espiritu, L. E. Heisler, F. Yousif, V. Huang, T. N. Yamaguchi, C. Q. Yao, V. Y. Sabelnykova, M. Fraser, M. L. K. Chua, T. van der Kwast, S. K. Liu, P. C. Boutros, R. G. Bristow, Molecular landmarks of tumor hypoxia across cancer types. *Nat. Genet.* **51**, 308–318 (2019).

16. B. Muz, P. de la Puente, F. Azab, A. K. Azab, The role of hypoxia in cancer progression, angiogenesis, metastasis, and resistance to therapy. *Hypoxia (Auckl)* **3**, 83–92 (2015).

17. J. Tao, G. Yang, W. Zhou, J. Qiu, G. Chen, W. Luo, F. Zhao, L. You, L. Zheng, T. Zhang, Y. Zhao, Targeting hypoxic tumor microenvironment in pancreatic cancer. *J. Hematol. Oncol.* **14**, 14 (2021).

18. P. Wang, S. Gong, J. Pan, J. Wang, D. Zou, S. Xiong, L. Zhao, Q. Yan, Y. Deng, N. Wu, B. Liao, Hyperbaric oxygen promotes not only glioblastoma proliferation but also chemosensitization by inhibiting HIF1α/HIF2α-Sox2. *Cell Death Discov.* **7**, 103 (2021).

19. E. E. Wicks, G. L. Semenza, Hypoxia-inducible factors: Cancer progression and clinical translation. *J. Clin. Invest.* **132**, e159839 (2022).

20. L. Wu, W. Wu, J. Zhang, Z. Zhao, L. Li, M. Zhu, M. Wu, F. Wu, F. Zhou, Y. Du, R.-C. Chai, W. Zhang, X. Qiu, Q. Liu, Z. Wang, J. Li, K. Li, A. Chen, Y. Jiang, X. Xiao, H. Zou, R. Srivastava, T. Zhang, Y. Cai, Y. Liang, B. Huang, R. Zhang, F. Lin, L. Hu, X. Wang, X. Qian, S. Lv, B. Hu, S. Zheng, Z. Hu, H. Shen, Y. You, R. G. W. Verhaak, T. Jiang, Q. Wang, Natural coevolution of tumor and immunoenvironment in glioblastoma. *Cancer Discov.* **12**, 2820–2837 (2022).

21. P. Lee, N. S. Chandel, M. C. Simon, Cellular adaptation to hypoxia through hypoxia inducible factors and beyond. *Nat. Rev. Mol. Cell Biol.* **21**, 268–283 (2020).

22. V. Bachanova, S. Cooley, T. E. Defor, M. R. Verneris, B. Zhang, D. H. McKenna, J. Curtsinger, A. Panoskaltsis-Mortari, D. Lewis, K. Hippen, P. McGlave, D. J. Weisdorf, B. R. Blazar, J. S. Miller, Clearance of acute myeloid leukemia by haploidentical natural killer cells is improved using IL-2 diphtheria toxin fusion protein. *Blood* **123**, 3855–3863 (2014).

23. S. Sarkar, W. T. V. Germeraad, K. M. A. Rouschop, E. M. P. Steeghs, M. van Gelder, G. M. J. Bos, L. Wieten, Hypoxia induced impairment of NK cell cytotoxicity against multiple myeloma can be overcome by IL-2 activation of the NK cells. *PLoS One* **8**, e64835 (2013).

24. M. Parodi, F. Raggi, D. Cangelosi, C. Manzini, M. Balsamo, F. Blengio, A. Eva, L. Varesio, G. Pietra, L. Moretta, M. C. Mingari, M. Vitale, M. C. Bosco, Hypoxia modifies the transcriptome of human NK cells, modulates their immunoregulatory profile, and influences NK cell subset migration. *Front. Immunol.* **9**, 2358 (2018).

25. S. Y. Velásquez, D. Killian, J. Schulte, C. Sticht, M. Thiel, H. A. Lindner, Short term hypoxia synergizes with interleukin 15 priming in driving glycolytic gene transcription and supports human natural killer cell activities. *J. Biol. Chem.* **291**, 12960–12977 (2016).

26. K. Solocinski, M. R. Padget, K. P. Fabian, B. Wolfson, F. Cecchi, T. Hembrough, S. C. Benz, S. Rabizadeh, P. Soon-Shiong, J. Schlom, J. W. Hodge, Overcoming hypoxia-induced functional suppression of NK cells. *J. Immunother. Cancer* **8**, e000246 (2020).

27. M. Balsamo, C. Manzini, G. Pietra, F. Raggi, F. Blengio, M. C. Mingari, L. Varesio, L. Moretta, M. C. Bosco, M. Vitale, Hypoxia downregulates the expression of activating receptors involved in NK-cell-mediated target cell killing without affecting ADCC. *Eur. J. Immunol.* **43**, 2756–2764 (2013).

28. X. Zheng, Y. Qian, B. Fu, D. Jiao, Y. Jiang, P. Chen, Y. Shen, H. Zhang, R. Sun, Z. Tian, H. Wei, Mitochondrial fragmentation limits NK cell-based tumor immunosurveillance. *Nat. Immunol.* **20**, 1656–1667 (2019).

29. J. Ni, X. Wang, A. Stojanovic, Q. Zhang, M. Wincher, L. Bühler, A. Arnold, M. P. Correia, M. Winkler, P.-S. Koch, V. Sexl, T. Höfer, A. Cerwenka, Single-cell RNA sequencing of tumor-infiltrating NK cells reveals that inhibition of transcription factor HIF-1α unleashes NK cell activity. *Immunity* **52**, 1075–1087.e8 (2020).

30. E. Krzywinska, C. Kantari-Mimoun, Y. Kerdiles, M. Sobecki, T. Isagawa, D. Gotthardt, M. Castells, J. Haubold, C. Millien, T. Viel, B. Tavitian, N. Takeda, J. Fandrey, E. Vivier, V. Sexl, C. Stockmann, Loss of HIF-1α in natural killer cells inhibits tumour growth by stimulating non-productive angiogenesis. *Nat. Commun.* **8**, 1597 (2017).

31. A. Pelletier, E. Nelius, Z. Fan, E. Khatchatourova, A. Alvarado-Diaz, J. He, E. Krzywinska, M. Sobecki, S. Nagarajan, Y. Kerdiles, J. Fandrey, D. Gotthardt, V. Sexl, K. de Bock, C. Stockmann, Resting natural killer cell homeostasis relies on tryptophan/ NAD^+ metabolism and HIF-1α. *EMBO Rep.*, **24**, e56156 (2023).

32. N. Bannoud, T. Dalotto-Moreno, L. Kindgard, P. A. García, A. G. Blidner, K. V. Mariño, G. A. Rabinovich, D. O. Croci, Hypoxia supports differentiation of terminally exhausted CD8 T cells. *Front. Immunol.* **12**, 660944 (2021).

33. Y.-N. Liu, J.-F. Yang, D.-J. Huang, H.-H. Ni, C.-X. Zhang, L. Zhang, J. He, J.-M. Gu, H.-X. Chen, H.-Q. Mai, Q.-Y. Chen, X.-S. Zhang, S. Gao, J. Li, Hypoxia induces mitochondrial defect that promotes T cell exhaustion in tumor microenvironment through MYC-regulated pathways. *Front. Immunol.* **11**, 1906 (2020).

34. N. E. Scharping, D. B. Rivadeneira, A. V. Menk, P. D. A. Vignali, B. R. Ford, N. L. Rittenhouse, R. Peralta, Y. Wang, Y. Wang, K. DePeaux, A. C. Poholek, G. M. Delgoffe, Mitochondrial stress induced by continuous stimulation under hypoxia rapidly drives T cell exhaustion. *Nat. Immunol.* **22**, 205–215 (2021).

35. N. E. Scharping, A. V. Menk, R. S. Moreci, R. D. Whetstone, R. E. Dadey, S. C. Watkins, R. L. Ferris, G. M. Delgoffe, The tumor microenvironment represses T cell mitochondrial biogenesis to drive intratumoral T cell metabolic insufficiency and dysfunction. *Immunity* **45**, 374–388 (2016).

36. Y.-R. Yu, H. Imrichova, H. Wang, T. Chao, Z. Xiao, M. Gao, M. Rincon-Restrepo, F. Franco, R. Genolet, W.-C. Cheng, C. Jandus, G. Coukos, Y.-F. Jiang, J. W. Locasale, A. Zippelius, P.-S. Liu, L. Tang, C. Bock, N. Vannini, P.-C. Ho, Disturbed mitochondrial dynamics in $CD8^+$ TILs reinforce T cell exhaustion. *Nat. Immunol.* **21**, 1540–1551 (2020).

37. S. E. Keating, V. Zaiatz-Bittencourt, R. M. Loftus, C. Keane, K. Brennan, D. K. Finlay, C. M. Gardiner, Metabolic reprogramming supports IFN-γ production by CD56bright NK cells. *J. Immunol.* **196**, 2552–60 (2016).

38. A. Marçais, J. Cherfils-Vicini, C. Viant, S. Degouve, S. Viel, A. Fenis, J. Rabilloud, K. Mayol, A. Tavares, J. Bienvenu, Y. G. Gangloff, E. Gilson, E. Vivier, T. Walzer, The metabolic checkpoint kinase mTOR is essential for IL-15 signaling during the development and activation of NK cells. *Nat. Immunol.* **15**, 749–757 (2014).

39. I. Terrén, A. Orrantia, A. Mosteiro, J. Vitallé, O. Zenarruzabeitia, F. Borrego, Metabolic changes of Interleukin-12/15/18-stimulated human NK cells. *Sci. Rep.* **11**, 6472 (2021).

40. J. R. Schafer, T. C. Salzillo, N. Chakravarti, M. N. Kararoudi, P. Trikha, J. A. Foltz, R. Wang, S. Li, D. A. Lee, Education-dependent activation of glycolysis promotes the cytolytic potency of licensed human natural killer cells. *J. Allergy Clin. Immunol.* **143**, 346–358.e6 (2019).

41. Z. Wang, D. Guan, S. Wang, L. Y. A. Chai, S. Xu, K.-P. Lam, Glycolysis and oxidative phosphorylation play critical roles in natural killer cell receptor-mediated natural killer cell functions. *Front. Immunol.* **11**, 202 (2020).

42. M. P. Keppel, N. Saucier, A. Y. Mah, T. P. Vogel, M. A. Cooper, Activation-specific metabolic requirements for NK Cell IFN-γ production. *J. Immunol.* **194**, 1954–1962 (2015).

43. E. Ortiz-Prado, J. F. Dunn, J. Vasconez, D. Castillo, G. Viscor, Partial pressure of oxygen in the human body: A general review. *Am. J. Blood Res.* **9**, 1–14 (2019).

44. C.-H. Heldin, K. Rubin, K. Pietras, A. Östman, High interstitial fluid pressure—An obstacle in cancer therapy. *Nat. Rev. Cancer* **4**, 806–813 (2004).

45. H. D. Roh, Y. Boucher, S. Kalnicki, R. Buchsbaum, W. D. Bloomer, R. K. Jain, Interstitial hypertension in carcinoma of uterine cervix in patients: Possible correlation with tumor oxygenation and radiation response. *Cancer Res.* **51**, 6695–6698 (1991).

46. T. Yu, K. Liu, Y. Wu, J. Fan, J. Chen, C. Li, G. Zhu, Z. Wang, L. Li, High interstitial fluid pressure promotes tumor cell proliferation and invasion in oral squamous cell carcinoma. *Int. J. Mol. Med.* **32**, 1093–1100 (2013).

47. S. W. Stonier, K. S. Schluns, Trans-presentation: A novel mechanism regulating IL-15 delivery and responses. *Immunol. Lett.* **127**, 85–92 (2010).

48. E. M. Mace, P. Dongre, H.-T. Hsu, P. Sinha, A. M. James, S. S. Mann, L. R. Forbes, L. B. Watkin, J. S. Orange, Cell biological steps and checkpoints in accessing NK cell cytotoxicity. *Immunol. Cell Biol.* **92**, 245–55 (2014).

49. N. Anfossi, P. André, S. Guia, C. S. Falk, S. Roetynck, C. A. Stewart, V. Breso, C. Frassati, D. Reviron, D. Middleton, F. Romagné, S. Ugolini, E. Vivier, Human NK cell education by inhibitory receptors for MHC class I. *Immunity* **25**, 331–42 (2006).

50. N. C. Fernandez, E. Treiner, R. E. Vance, A. M. Jamieson, S. Lemieux, D. H. Raulet, A subset of natural killer cells achieves self-tolerance without expressing inhibitory receptors specific for self-MHC molecules. *Blood* **105**, 4416–4423 (2005).

51. S. Kim, J. Poursine-Laurent, S. Truscott, L. Lybarger, Y.-J. Song, L. Yang, A. R. French, J. B. Sunwoo, S. Lemieux, T. H. Hansen, W. M. Yokoyama, Licensing of natural killer cells by host major histocompatibility complex class I molecules. *Nature* **436**, 709–713 (2005).

52. S. Gill, A. E. Vasey, A. De Souza, J. Baker, A. T. Smith, H. E. Kohrt, M. Florek, K. D. Gibbs, K. Tate, D. S. Ritchie, R. S. Negrin, Rapid development of exhaustion and down-regulation of eomesodermin limit the antitumor activity of adoptively transferred murine natural killer cells. *Blood* **119**, 5758–5768 (2012).

53. S. M. Gordon, J. Chaix, L. J. Rupp, J. Wu, S. Madera, J. C. Sun, T. Lindsten, S. L. Reiner, The transcription factors T-bet and Eomes control key checkpoints of natural killer cell maturation. *Immunity* **36**, 55–67 (2012).

54. L. Kiekens, W. Van Loocke, S. Taveirne, S. Wahlen, E. Persyn, E. Van Ammel, Z. De Vos, P. Matthys, F. Van Nieuwerburgh, T. Taghon, P. Van Vlierberghe, B. Vandekerckhove, G. Leclercq, T-BET and EOMES accelerate and enhance functional differentiation of human natural killer cells. *Front. Immunol.* **12**, 732511 (2021).

55. E. Hegewisch-Solloa, A. P. Nalin, A. G. Freud, E. M. Mace, Deciphering the localization and trajectory of human natural killer cell development. *J. Leukoc. Biol.* **114**, 487–506 (2023).

56. E. Hegewisch-Solloa, S. Seo, B. L. Mundy-Bosse, A. Mishra, E. H. Waldman, S. Maurrasse, E. Grunstein, T. J. Connors, A. G. Freud, E. M. Mace, Differential integrin adhesome expression defines human NK cell residency and developmental stage. *J. Immunol.* **207**, 950–965 (2021).

57. N. K. Björkström, P. Riese, F. Heuts, S. Andersson, C. Fauriat, M. A Ivarsson, A. T. Björklund, M. Flodström-Tullberg, J. Michaëlsson, M. E. Rottenberg, C. A Guzmán, H.-G. Ljunggren, K.-J. Malmberg, Expression patterns of NKG2A, KIR, and CD57 define a process of CD56dim NK cell differentiation uncoupled from NK cell education. *Blood* **116**, 3853–3864 (2010).

58. T. Beppu, K. Kamada, Y. Yoshida, H. Arai, K. Ogasawara, A. Ogawa, Change of oxygen pressure in glioblastoma tissue under various conditions. *J. Neurooncol* **58**, 47–52 (2002).

59. P. Vaupel, M. Höckel, A. Mayer, Detection and characterization of tumor hypoxia using pO2 histography. *Antioxid. Redox Signal.* **9**, 1221–1236 (2007).

60. C. Yang, J. R. Siebert, R. Burns, Z. J. Gerbec, B. Bonacci, A. Rymaszewski, M. Rau, M. J. Riese, S. Rao, K. S. Carlson, J. M. Routes, J. W. Verbsky, M. S. Thakar, S. Malarkannan, Heterogeneity of human bone marrow and blood natural killer cells defined by single-cell transcriptome. *Nat. Commun.* **10**, 3931 (2019).

61. D. B. Zamler, T. Shingu, L. M. Kahn, K. Huntoon, C. Kassab, M. Ott, K. Tomczak, J. Liu, Y. Li, I. Lai, R. Zorilla-Veloz, C. Yee, K. Rai, B. Y. Kim, S. S. Watowich, A. B. Heimberger, G. F. Draetta, J. Hu, Immune landscape of a genetically engineered murine model of glioma compared with human glioma. *JCI Insight* **7**, e148990 (2022).

62. H. Shaim, M. Shanley, R. Basar, M. Daher, J. Gumin, D. B. Zamler, N. Uprety, F. Wang, Y. Huang, K. Gabrusiewicz, Q. Miao, J. Dou, A. Alsuliman, L. N. Kerbauy, S. Acharya, V. Mohanty, M. Mendt, S. Li, J. Lu, J. Wei, N. W. Fowlkes, E. Gokdemir, E. L. Ensley, M. Kaplan, C. Kassab, L. Li, G. Ozcan, P. P. Banerjee, Y. Shen, A. L. Gilbert, C. M. Jones, M. Bdiwi, A. K. Nunez-Cortes, E. Liu, J. Yu, N. Imahashi, L. Muniz-Feliciano, Y. Li, J. Hu, G. Draetta, D. Marin, D. Yu, S. Mielke, M. Eyrich, R. E. Champlin, K. Chen, F. F. Lang, E. J. Shpall, A. B. Heimberger, K. Rezvani, Targeting the αv integrin/TGF-β axis improves natural killer cell function against glioblastoma stem cells. *J. Clin. Invest.* **131**, e142116 (2021).

63. T. E. O'Sullivan, L. R. Johnson, H. H. Kang, J. C. Sun, BNIP3- and BNIP3L-mediated mitophagy promotes the generation of natural killer cell memory. *Immunity* **43**, 331–342 (2015).

64. N. C. Denko, Hypoxia, HIF1 and glucose metabolism in the solid tumour. *Nat. Rev. Cancer* **8**, 705–713 (2008).

65. K. V. Woan, H. Kim, R. Bjordahl, Z. B. Davis, S. Gaidarova, J. Goulding, B. Hancock, S. Mahmood, R. Abujarour, H. Wang, K. Tuininga, B. Zhang, C.-Y. Wu, B. Kodal, M. Khaw, L. Bendzick, P. Rogers, M. Q. Ge, G. Bonello, M. Meza, M. Felices, J. Huffman, T. Dailey, T. T. Lee, B. Walcheck, K. J. Malmberg, B. R. Blazar, Y. T. Bryceson, B. Valamehr, J. S. Miller, F. Cichocki, Harnessing features of adaptive NK cells to generate iPSC-derived NK cells for enhanced immunotherapy. *Cell Stem Cell* **28**, 2062–2075.e5 (2021).

66. L. Li, V. Mohanty, J. Dou, Y. Huang, P. P. Banerjee, Q. Miao, J. G. Lohr, T. Vijaykumar, J. Frede, B. Knoechel, L. Muniz-Feliciano, T. J. Laskowski, S. Liang, J. S. Moyes, V. Nandivada, R. Basar, M. Kaplan, M. Daher, E. Liu, Y. Li, S. Acharya, P. Lin, M. Shanley, H. Rafei, D. Marin, S. Mielke, R. E. Champlin, E. J. Shpall, K. Chen, K. Rezvani, Loss of metabolic fitness drives tumor resistance after CAR-NK cell therapy and can be overcome by cytokine engineering. *Sci. Adv.* **9**, eadd6997 (2023).

67. M. Lachota, K. Zielniok, D. Palacios, M. Kanaya, L. Penna, H. J. Hoel, M. T. Wiiger, L. Kveberg, W. Hautz, R. Zagożdżon, K.-J. Malmberg, Mapping the chemotactic landscape in NK cells reveals subset-specific synergistic migratory responses to dual chemokine receptor ligation. *EBioMedicine* **96**, 104811 (2023).

68. R. D. Berahovich, N. L. Lai, Z. Wei, L. L. Lanier, T. J. Schall, Evidence for NK cell subsets based on chemokine receptor expression. *J. Immunol.* **177**, 7833–7840 (2006).

69. E. Cluff, C. C. Magdaleno, E. Fernandez, T. House, S. Swaminathan, A. Varadaraj, N. Rajasekaran, Hypoxia-inducible factor-1 alpha expression is induced by IL-2 via the PI3K/mTOR pathway in hypoxic NK cells and supports effector functions in NKL cells and ex vivo expanded NK cells. *Cancer Immunol. Immunother.* **71**, 1989–2005 (2022).

70. A. Coulibaly, S. Y. Velásquez, N. Kassner, J. Schulte, M. V. Barbarossa, H. A. Lindner, STAT3 governs the HIF-1α response in IL-15 primed human NK cells. *Sci. Rep.*, **11**, 7023 (2021).

71. G. L. Semenza, Targeting HIF-1 for cancer therapy. *Nat. Rev. Cancer* **3**, 721–732 (2003).

72. M. F. Kaminski, L. Bendzick, R. Hopps, M. Kauffman, B. Kodal, Y. Soignier, P. Hinderlie, J. T. Walker, T. R. Lenvik, M. A. Geller, J. S. Miller, M. Felices, TEM8 tri-specific killer engager binds both tumor and tumor stroma to specifically engage natural killer cell anti-tumor activity. *J. Immunother. Cancer* **10**, e004725 (2022).

73. P. R. Kennedy, D. A. Vallera, B. Ettestad, C. Hallstrom, B. Kodal, D. A. Todhunter, L. Bendzick, P. Hinderlie, J. T. Walker, B. Pulkrabek, I. Pastan, R. A. Kratzke, N. Fujioka, J. S. Miller, M. Felices, A tri-specific killer engager against mesothelin targets NK cells towards lung cancer. *Front. Immunol.* **14**, 1060905 (2023).

74. C. T. Rieger, M. Fiegl, Microenvironmental oxygen partial pressure in acute myeloid leukemia: Is there really a role for hypoxia? *Exp. Hematol.* **44**, 578–582 (2016).

75. R. Romee, M. Rosario, M. M. Berrien-Elliott, J. A. Wagner, B. A. Jewell, T. Schappe, J. W. Leong, S. Abdel-Latif, S. E. Schneider, S. Willey, C. C. Neal, L. Yu, S. T. Oh, Y.-S. S. Lee, A. Mulder, F. Claas, M. A. Cooper, T. A. Fehniger, Cytokine-induced memory-like natural killer cells exhibit enhanced responses against myeloid leukemia. *Sci. Transl. Med.* **8**, 357ra123 (2016).

76. M. M. Berrien-Elliott, M. Becker-Hapak, A. F. Cashen, M. Jacobs, P. Wong, M. Foster, E. McClain, S. Desai, P. Pence, S. Cooley, C. Brunstein, F. Gao, C. N. Abboud, G. L. Uy, P. Westervelt, M. A. Jacoby, I. Pusic, K. E. Stockerl-Goldstein, M. A. Schroeder, J. F. DiPersio, P. Soon-Shiong, J. S. Miller, T. A. Fehniger, Systemic IL-15 promotes allogeneic cell rejection in patients treated with natural killer cell adoptive therapy. *Blood* **139**, 1177–1183 (2022).

77. S. O. Ciurea, J. R. Schafer, R. Bassett, C. J. Denman, K. Cao, D. Willis, G. Rondon, J. Chen, D. Soebbing, I. Kaur, A. Gulbis, S. Ahmed, K. Rezvani, E. J. Shpall, D. A. Lee, R. E. Champlin, Phase 1 clinical trial using mbIL21 ex vivo-expanded donor-derived NK cells after haploidentical transplantation. *Blood* **130**, 1857–1868 (2017).

78. G. A. Gusarova, H. E. Trejo, L. A. Dada, A. Briva, L. C. Welch, R. B. Hamanaka, G. M. Mutlu, N. S. Chandel, M. Prakriya, J. I. Sznajder, Hypoxia leads to Na,K-ATPase downregulation via Ca^{2+} release-activated Ca^{2+} channels and AMPK activation. *Mol. Cell. Biol.* **31**, 3546–3556 (2011).

79. S. Sheppard, E. K. Santosa, C. M. Lau, S. Violante, P. Giovanelli, H. Kim, J. R. Cross, M. O. Li, J. C. Sun, Lactate dehydrogenase A-dependent aerobic glycolysis promotes natural killer cell anti-viral and anti-tumor function. *Cell Rep.* **35**, 109210 (2021).

80. S. Y. Velásquez, B. S. Himmelhan, N. Kassner, A. Coulibaly, J. Schulte, K. Brohm, H. A. Lindner, Innate cytokine induced early release of IFNγ and CC chemokines from hypoxic human NK cells is independent of glucose. *Cells* **9**, 734 (2020).

81. A. Y. Mah-Som, M. P. Keppel, J. M. Tobin, A. Kolicheski, N. Saucier, V. Sexl, A. R. French, J. A. Wagner, T. A. Fehniger, M. A. Cooper, Reliance on Cox10 and oxidative metabolism for antigen-specific NK cell expansion. *Cell Rep.* **35**, 109209 (2021).

82. T. Nakazawa, T. Morimoto, R. Maeoka, K. Yamada, R. Matsuda, M. Nakamura, F. Nishimura, S. Yamada, Y.-S. Park, T. Tsujimura, I. Nakagawa, Characterization of HIF-1α knockout primary human natural killer cells including populations in allogeneic glioblastoma. *Int. J. Mol. Sci.* **25**, 5896 (2024).

83. G. L. Semenza, B.-H. Jiang, S. W. Leung, R. Passantino, J.-P. Concordet, P. Maire, A. Giallongo, Hypoxia response elements in the aldolase A, enolase 1, and lactate dehydrogenase A gene promoters contain essential binding sites for hypoxia-inducible factor 1. *J. Biol. Chem.* **271**, 32529–32537 (1996).

84. W. W. Wheaton, N. S. Chandel, Hypoxia. 2. Hypoxia regulates cellular metabolism. *Am. J. Physiol. Cell Physiol.* **300**, C385–C393 (2011).

85. M. Felices, A. J. Lenvik, R. McElmurry, S. Chu, P. Hinderlie, L. Bendzick, M. A. Geller, J. Tolar, B. R. Blazar, J. S. Miller, Continuous treatment with IL-15 exhausts human NK cells via a metabolic defect. *JCI insight* **3**, e96219 (2018).

86. K. DePeaux, G. M. Delgoffe, Metabolic barriers to cancer immunotherapy. *Nat. Rev. Immunol.* **21**, 785–797 (2021).

87. S. Kundu, M. Gurney, M. O'Dwyer, Generating natural killer cells for adoptive transfer: Expanding horizons. *Cytotherapy* **23**, 559–566 (2021).

88. E. O. Ojo, A. A. Sharma, R. Liu, S. Moreton, M.-A. Checkley-Luttge, K. Gupta, G. Lee, D. A. Lee, F. Otegbeye, R.-P. Sekaly, M. de Lima, D. N. Wald, Membrane bound IL-21 based NK cell feeder cells drive robust expansion and metabolic activation of NK cells. *Sci. Rep.* **9**, 14916 (2019).

89. Y. Yang, S. Badeti, H. Tseng, M. T. Ma, T. Liu, J.-G. Jiang, C. Liu, D. Liu, Superior expansion and cytotoxicity of human primary NK and CAR-NK cells from various sources via enriched metabolic pathways. *Mol. Ther. Methods Clin. Dev.* **18**, 428–445 (2020).

90. D. A. Lee, Cellular therapy: Adoptive immunotherapy with expanded natural killer cells. *Immunol. Rev.* **290**, 85–99 (2019).

91. C. J. Denman, V. V. Senyukov, S. S. Somanchi, P. V. Phatarpekar, L. M. Kopp, J. L. Johnson, H. Singh, L. Hurton, S. N. Maiti, M. H. Huls, R. E. Champlin, L. J. N. Cooper, D. A. Lee, Membrane-bound IL-21 promotes sustained ex vivo proliferation of human natural killer cells. *PLOS ONE* **7**, e30264 (2012).

92. M. Chang, X. Tang, L. Nelson, G. Nyberg, Z. Du, Differential effects on natural killer cell production by membrane-bound cytokine stimulations. *Biotechnol. Bioeng.* **119**, 1820–1838 (2022).

93. A. V. Menk, N. E. Scharping, D. B. Rivadeneira, M. J. Calderon, M. J. Watson, D. Dunstane, S. C. Watkins, G. M. Delgoffe, 4-1BB costimulation induces T cell mitochondrial function and biogenesis enabling cancer immunotherapeutic responses. *J. Exp. Med.* **215**, 1091–1100 (2018).

94. P. F. Fiore, S. Di Matteo, N. Tumino, F. R. Mariotti, G. Pietra, S. Ottonello, S. Negrini, B. Bottazzi, L. Moretta, E. Mortier, B. Azzarone, Interleukin-15 and cancer: Some solved and many unsolved questions. *J. Immunother. Cancer* **8**, e001428 (2020).

95. S. S. Somanchi, V. V. Senyukov, C. J. Denman, D. A. Lee, Expansion, purification, and functional assessment of human peripheral blood NK cells. *J. Vis. Exp.*, **2**, 2540 (2011).

96. H. Zhu, R. H. Blum, R. Bjordahl, S. Gaidarova, P. Rogers, T. T. Lee, R. Abujarour, G. B. Bonello, J. Wu, P. Tsai, J. S. Miller, B. Walcheck, B. Valamehr, D. S. Kaufman, Pluripotent stem cell–derived NK cells with high-affinity noncleavable CD16a mediate improved antitumor activity. *Blood* **135**, 399–410 (2020).

97. A. Pfefferle, B. Jacobs, H. Netskar, E. H. Ask, S. Lorenz, T. Clancy, J. P. Goodridge, E. Sohlberg, K.-J. Malmberg, Intra-lineage plasticity and functional reprogramming maintain natural killer cell repertoire diversity. *Cell Rep.* **29**, 2284–2294.e4 (2019).

98. U. S. Arvindam, "Increasing specificity and overcoming hypoxia in the tumor microenvironment to optimize anti-tumor activity of human natural killer cells," thesis, University of Minnesota (2022).

99. A. C. Shore, Capillaroscopy and the measurement of capillary pressure. *Br. J. Clin. Pharmacol.* **50**, 501–513 (2000).

100. U. A. Gurkan, O. Akkus, The mechanical environment of bone marrow: A review. *Ann. Biomed. Eng.* **36**, 1978–1991 (2008).

101. X. Fu, S. Deja, B. Kucejova, J. A. G. Duarte, J. G. McDonald, S. C. Burgess, Targeted determination of tissue energy status by LC-MS/MS. *Anal. Chem.* **91**, 5881–5887 (2019).

102. D. B. Stagg, J. R. Gillingham, A. B. Nelson, J. E. Lengfeld, D. A. d'Avignon, P. Puchalska, P. A. Crawford, Diminished ketone interconversion, hepatic TCA cycle flux, and glucose production in D-β-hydroxybutyrate dehydrogenase hepatocyte-deficient mice. *Mol. Metab.* **53**, 101269 (2021).
